# Understanding epigenetic regulation in nonmodel plants: transcriptomic responses to seed demethylation in leaves and roots of the annual herb *Erodium cicutarium* (Geraniaceae)

**DOI:** 10.1093/g3journal/jkag129

**Published:** 2026-05-15

**Authors:** Rubén Martín-Blázquez, Mónica Medrano, Conchita Alonso

**Affiliations:** Estación Biológica de Doñana, CSIC, Avenida Américo Vespucio 26, Sevilla 41092, Spain; Estación Biológica de Doñana, CSIC, Avenida Américo Vespucio 26, Sevilla 41092, Spain; Estación Biológica de Doñana, CSIC, Avenida Américo Vespucio 26, Sevilla 41092, Spain

**Keywords:** 5-azacytidine, experimental seed demethylation, DNA methylation, genome assembly, RNA-seq, plant epigenetics

## Abstract

Epigenetic regulation, and particularly modifications in DNA methylation, plays critical roles in plant adaptation. DNA methylation inhibitors have been used to investigate the relationship between DNA methylation and plastic plant phenotypes. However, their effect in gene expression regulation throughout the lifetime remains understudied in nonmodel plants. Here, we analyze the effects of seed exposure to the hypomethylating agent 5-azacytidine (5-azaC) in plant transcriptome. Seeds of *Erodium cicutarium* were soaked in either a solution of 5-azaC in DMSO or water with DMSO (control, hereafter) before sowing. Subsequently, RNA was extracted from juvenile roots, juvenile leaves, and adult leaves, and their transcriptomes were sequenced. Differential gene expression analysis was performed between control and treated samples for all tissues together and separately, presuming that changes between treatments will be substantially weaker than among tissues and developmental stages. Beforehand, a draft genome of *E. cicutarium* was assembled as reference for the transcriptome analysis. We found that 5-azaC upregulated chromomethylase *CMT1* across all treated samples, and domain rearranged DNA methyltransferase *DRM2* in juvenile roots. Furthermore, adult leaves showed more differentially expressed genes between control and 5-azaC-treated samples compared to juvenile leaves. Finally, gene co-expression network analysis revealed a module of co-expressed genes with differential gene expression linked to 5-azaC treatment in juvenile roots, pointing toward activation of genes associated with transposable elements. These results show how experimental treatment with 5-azaC at seed stage has both short and long-term effects in the plant transcriptome, potentially broadening phenotype variation due to nondirectional effects of 5-azaC on gene expression.

## Introduction

Epigenetic regulation comprises various interrelated mechanisms that can contribute to phenotypic variation without modifying the DNA sequence (Henderson and Jacobsen 2007), including histone and chromatin modification (Fransz and De Jong 2002; Du et al. 2015), gene expression regulation mediated by small interfering RNA (siRNA) (Matzke and Mosher 2014), and genomic DNA methylation (Zilberman et al. 2007). Regarding the latter, plants in particular show an extended DNA methylation machinery compared to other organisms: not only cytosines in CpG positions are sensitive to methylation but also those in CHG and CHH positions (H being A, C, T nucleotides) (Law and Jacobsen 2010). In addition to the DNA methyltransferases *DNMTs*, which maintain DNA methylation in CpG positions and are evolutionarily conserved across eukaryotes, plant-specific chromomethylases (*CMTs*), and domain rearranged DNA methyltransferases (*DRMs*) in cross-talk with histone mark modification, are in charge of de novo methylation in CHH and its maintenance in CHG positions as well (Cao and Jacobsen 2002; Bewick et al. 2017). Moreover, de novo cytosine methylation in any sequence context is carried out by *DRM2* through a siRNA-directed DNA methyltransferase pathway (RdDM) unique to plants (Cao et al. 2003; Henderson et al. 2010; Matzke and Mosher 2014). Finally, plants possess a unique mechanism of active DNA demethylation that involves a family of 5-methylcytosine-specific DNA glycosylases, including DEMETER and repressor of silencing (ROS), which play a critical role in modulating the activity of transposable elements (TEs) and gene expression (Law and Jacobsen 2010).

While DNA methylation in CpG can be a stable epigenetic mark in certain genomic regions (eg gene body methylation; Bewick et al. 2017), being suitable even for the reconstruction and dating of shallow phylogenies (Yao et al. 2023), more dynamic changes can be observed in other genomic regions (Yao et al. 2023) and sequence contexts that routinely differed between tissues, organs, and developmental stages (Widman et al. 2014; Bhatia et al. 2018; Gutzat et al. 2020; N'Diaye et al. 2020) or may change in response to stress (Gallusci et al. 2023 and references therein; Yao et al. 2023). Distinguishing between these 2 conditions makes the interpretation of experiments designed to understand the contribution of epigenetic regulation in plant adaptation very challenging in nonmodel plants, which usually lack good-quality genomic resources (Richards et al. 2017). DNA methylation inhibitors have been used for decades to generate phenotypic innovations and investigate the association between DNA methylation and phenotypic plasticity (Dvořák Tomaštíková and Pecinka 2025). In particular, the cytidine analog 5-azacytidine (5-azaC) has been used to experimentally manipulate DNA methylation, providing the opportunity to decouple epigenetic regulation from other experimental factors suspected to be dependent on DNA methylation (Lang et al. 2017; Huang et al. 2019; Jia et al. 2020; Osorio-Montalvo et al. 2020). As a cytidine analog, 5-azaC is incorporated into the genomic DNA during each replication event, binding covalently to DNA methyltransferases (DNMTs), and therefore causing a decline in DNA methylation levels (Christman 2002; Stresemann and Lyko 2008). Several plant transcriptomic studies show how these genes are upregulated by 5-azaC (Xu et al. 2017; Kiselev et al. 2019), whereas others report downregulation (Zheng et al. 2023). Unfortunately, the transcriptomic response to 5-azaC has always been studied in seedlings of model plants with small genomes (Griffin et al. 2016; Xu et al. 2017; Kiselev et al. 2019; Zhang et al. 2020), leaving wild nonmodel plant species in the dark. Still, 5′-azacytidine has been proven to reduce global DNA methylation levels in several plant species (Fieldes et al. 2005; Alonso et al. 2019a; Browne et al. 2020; Osorio-Montalvo et al. 2020; Troyee et al. 2022), as well as produce changes in phenotype (Prakash and Kumar 1997; Fieldes and Amyot 2000; Bossdorf et al. 2010; Munsamy et al. 2013; Alonso et al. 2026). Thus, understanding the consequences of 5-azaC treatment on gene expression and developmental regulation in species with different genome features could be helpful to better understand its mode of action, potential side effects (see Dvořák Tomaštíková and Pecinka 2025 for a recent review), and the utility of 5-azaC exposure experiments for investigating the role of epigenetic regulation in plant adaptation to heterogeneous and stressful environments (Richards et al. 2017).

In this study, we exposed seeds of a single inbred line of the widespread Mediterranean annual herb *Erodium cicutarium* (Geraniaceae) to 5-azaC, following the same protocol that was known to slow individual growth, delay flowering, and reduce global DNA methylation in leaves and roots at adult stage with negative consequences for individual fitness (Alonso et al. 2026). In order to characterize changes in gene expression that could help to understand the phenotypic consequences recorded, we extracted and sequenced polyA-enriched RNA from roots and leaves of vegetative juvenile individuals and leaves of reproductive adult plants and compared the transcriptomes of treated and control plants. By comparing these samples, we aim to assess the variability of the transcriptomic response after seed demethylation treatment in both tissues and developmental stages of the plant. We hypothesized that seed exposure to 5-azaC (i) will affect the transcriptome response in the three tissues analyzed, (ii) the transcriptomic response will be stronger in the juvenile stage compared to the adult reproductive stage, and (iii) the transcriptome response will likely be more variable across individuals at the adult reproductive stage. In order to improve functional characterization of recorded changes, we also de novo assembled the genome sequence of *E. cicutarium*, paying particular attention to DNA methyltransferase (DNMT) genes.

## Methods

### Study species and genome assembly

*E. cicutarium* (L.) L'Hér. is a widespread, herbaceous annual plant with a fast life cycle and a high ability for autonomous selfing that is frequently associated with disturbed and ruderal habitats (Fiz-Palacios et al. 2006). Its genome size has been estimated to range from 1.2 to 2.7 Gbps (Zonneveld 2019). Different ploidy levels have been reported from different geographic regions, ranging from diploid to hexaploid (Fiz-Palacios et al. 2006; Zonneveld 2019). The study material was collected in the Cazorla mountains (Jaén province, SE Spain) where preliminary data suggest specimens have 2n = 40 chromosomes.

DNA was isolated from leaves of a single *E. cicutarium* individual, the F4 generation of a self-pollinated lineage (strain *F0-CH34_F3-Ecic127_F4-01*). Library preparation and sequencing were performed by AllGenetics (www.allgenetics.eu). Illumina paired-end short reads library was constructed using the HiSeq X Reagent Kit v2.5, sequenced in a HiSeq X PE150 (Illumina) with an Illumina patterned flow cell. PacBio long reads library was constructed using SMRTbell Express Template Prep Kit 2.0 (PacBio), sequenced with a Sequel II sequencer (PacBio), with an SMRT Cell 8 M under the long reads mode. The de novo genome assembly of *E. cicutarium* is described thoroughly in Supplementary Methods 1. In brief, short and long reads were assembled de novo into “mega-reads” using the software MaSuRCA v3.4.2 (Zimin et al. 2013) and then polished with POLCA (Zimin and Salzberg 2020). GenomeScope (Vurture et al. 2017) was used on the Illumina paired-end reads to infer genome length and ploidy. The quality and completeness of the genome assembly were evaluated using BUSCO V5.beta.1 (Simão et al. 2015). Further information about quality check, gene prediction, and protein annotation is thoroughly explained in Supplementary Methods 1.

### Characterization of DNA methyltransferases in *E. cicutarium* genome

Gene models annotated as DNA methyltransferases, chromomethylases, and domain rearranged DNA methyltransferases in the *E. cicutarium* genome assembly annotations were retrieved. Amino acid sequences encoding for DNA methyltransferases in *Arabidopsis thaliana* genome were retrieved from UniProtKB (The UniProt Consortium 2007), accessed on 2024 May 28 (accession numbers specified in Table 1). DNA methyltransferase protein sequences were also retrieved from the evolutionary close species *Geranium maculatum* (Geraniaceae) (Blanco and Leebens-Mack, unpub. data) using local BLASTp (Altschul et al. 1990, Camacho et al. 2009), which were curated with another BLASTp search against UniProtKB database (The UniProt Consortium 2007). The amino acid sequences from *A. thaliana*, *G. maculatum*, and *E. cicutarium* were aligned with the CLUSTAL Omega online toolset (Sievers et al. 2011), and a neighbor-joining tree with 500 bootstrap permutations was built with MEGA v12.0.10 to infer the phylogenetic relationships of *E. cicutarium* DNA methyltransferases. A second alignment including exclusively *E. cicutarium DNMT*, *CMT*, and *DRM* protein sequences was used to produce sequence logos of the catalytic conserved motifs shown in Pavlopoulou and Kossida (2007), using WebLogo (Crooks et al. 2004). All the amino acid sequences used for phylogenetic analysis are available in Supplementary Methods 2.

**Table 1.** DNA methyltransferase amino acid sequences retrieved for phylogenetic analyses. Species name, UniProtKB accession number, gene name, and description are shown in columns from first to fourth, respectively. Each row shows a sequence entry.

Species	Accession	Gene	Description
*A. thaliana*	A0A178UE49	MET1	DNA (cytosine-5)-methyltransferase (MET1)
	A0A7G2ELI8	DRM3	(thale cress) hypothetical protein (DRM3)
	A0A5S9Y438	DRM2	Uncharacterized protein (DRM2)
	Q9LXE5	DRM1	DNA (cytosine-5)-methyltransferase (DRM1)
	F4JWT7	DNMT2	tRNA (cytosine(38)-C(5))-methyltransferase 2 (DNMT2)
	O23273	DNMT2	DNA (cytosine-5)-methyltransferase 4 (DNMT2)
	A0A654EMJ3	CMT3	Cytosine-specific methyltransferase (CMT3)
	Q94F87	CMT2	DNA (cytosine-5)-methyltransferase (CMT2)
	O49139	CMT1	Putative DNA (cytosine-5)-methyltransferase (CMT1)

### Experimental exposure to 5-azaC

In this experiment, we used full-siblings to minimize genetic variation in the study sample that could interfere with the interpretation of the transcriptomic effects of the treatment. Twelve seeds obtained by autonomous self-pollination from a single maternal *E. cicutarium* plant were scarified with sandpaper and then soaked in 200 µL of either a control or a 500 µM 5-azaC (Sigma A2385, 100 mg, initially diluted to 100 mM in Sigma DMSO) solution for 2 d (Alonso et al. 2017). For the control solution, the same proportion of DMSO was diluted in water (3:97; v:v). Control and treated seeds were afterwards germinated individually in seedling nursery trays (4 × 4 × 7 cm) and grown in a climatic chamber (Aralab ClimaPus 400; with 12 h light and 22 °C: 18 °C) for 5 weeks until they reached juvenile stage, ie plants that have the cotyledons and the first 4 true leaves fully developed. Another group of 12 plants from a different experimental set (with identical 5-azaC and control exposure) was left to grow up over 12 weeks in the greenhouse (10 h light; 25 °C: 15 °C) to reach an adult stage, ie plants that have started blooming, to assess whether transcriptomic effects after seed treatment persist up to the reproductive adult stage.

### RNA extraction, sequencing, quality check, and alignment to genome

Fresh juvenile leaf (*N* = 12), juvenile root (*N* = 12), and adult leaf (*N* = 12) samples (10 to 100 mg) from each experimental plant were collected, immediately flash frozen in liquid nitrogen, and stored at −80 °C. Although leaf samples were collected from juvenile vegetative individuals and adult reproductive plants, we have always sampled fully developed and undamaged leaves in both life cycle stages. Root samples from adult individuals were not collected to avoid causing additional stress to the individuals, which were part of a longer-term experiment intended to explore phenotypic and reproductive consequences of experimental demethylation. RNA extraction, polyA RNA enrichment, and sequencing were performed by Macrogen Inc. (Seoul, Republic of South Korea). RNA root sample from juvenile individual *ecic05* did not pass the RNA extraction quality check and was discarded from sequencing. Paired-end reads of 151 bp length RNA-seq libraries were generated using TruSeq Stranded mRNA Library Prep kit (20020595, Illumina), and Illumina sequencing was performed using a NovaSeq6000 machine. Although highly interesting for epigenetic regulation, the small noncoding RNAs were not sequenced in our study because of limited funds. Reads are available at the NCBI Sequence Read Archive (SRA), under the BioProject accession numbers PRJNA1128899 (juvenile samples) and PRJNA1129331 (adult samples).

Read quality check was assessed with FastQC (Andrews 2010) for each step of the pipeline. Reads were trimmed out of low-quality positions and Illumina adapters with Trimmomatic v0.39 (Bolger et al. 2014), using the options “*-phred33 ILLUMINACLIP:$adapter_fasta_file:2:30:10 LEADING:28 TRAILING:28 SLIDINGWINDOW:4:15 MINLEN:30*,” and quality checked again with FastQC. Trimmed reads were aligned to the draft genome of *E. cicutarium* with STAR v2.7.10 (Dobin et al. 2012), with options “*–sjdbOverhang 149 –quantMode TranscriptomeSAM GeneCounts –twopassMode Basic –outSAMunmapped Within –outSAMtype BAM SortedByCoordinate*,” and again quality checked with FastQC. FastQC files were scanned with MultiQC v1.13.dev0 (Ewels et al. 2016) to summarize the number of reads and calculate fragment alignment rate to genome. Counts of fragments (paired reads) mapped to gene models were extracted with *featureCounts* v2.0.3 command from the Subread package (Liao et al. 2013), with options “*-p -s 0 -g Parent -t exon*.”

### Differential gene expression analysis

Genes in *E. cicutarium* genome with less than 100 fragment counts across all samples were discarded from subsequent analyses. Remaining raw fragment count per gene values were normalized using the DESeq2 median of ratios method (Love et al. 2014), and differential gene expression analysis was performed using DESeq2 by fitting expression data to the formula *expression ∼ 0 + group*, (*group* consisted 6 levels: control leaves from juvenile, control leaves from adult plants, 5-azaC leaves from juvenile, 5-azaC leaves from adult plants, control roots from juvenile, and 5-azaC roots from juvenile). Differential expression analysis was performed to test the effect of 5-azaC. We compared all control samples with all 5-azaC samples, followed by separate within-tissue and within-age comparisons between control and 5-azaC samples. A gene was considered a differentially expressed gene (DEG) when its false discovery rate (FDR) in any of the comparisons was below 0.05 (Benjamini and Hochberg 1995). The DEGs related to epigenetic regulation processes (including DNA methylation, chromatin modification, and siRNA regulation) are commented in the results. Overlapping DEGs between different DEG sets were tested to check whether the overlap was greater than expected by chance with the hypergeometric test using the *phyper* R function. All statistical analyses were performed in R v4.3.1 (R Core Team 2023).

### Gene co-expression network analysis

In addition to gene expression analysis, weighted gene co-expression networks were analyzed with the R package *WGCNA* (Langfelder and Horvath 2008) to generate groups of co-expressed genes (modules) associated with the control vs 5-azaC treatment. Gene modules were determined using the hybrid method implemented in the *blockwiseModules()* function, using the parameters “*maxBlockSize = 70973, networkType = “unsigned”, power = 6, detectCutHeight = 0.9, deepSplit = 4, minModuleSize = 50*.” To evaluate connectivity of genes within their modules, we calculated Kleinberg's hub centrality scores for all genes with the *hub_score()* function from the *igraph* R package (Csárdi and Nepusz 2006). PCA of module eigengenes (MEs) was performed to explore sample clustering and their relationships with the modules. To test the association between modules and treatments, module gene significances were calculated by transforming each module gene's FDR as −log_10_(FDR) and calculating its mean per module. Given there are 4 comparisons between control vs. 5-azaC treatment (all samples, juvenile roots, juvenile leaves, and adult leaves), each module showed 4 module gene significance values. Gene module memberships from modules with the highest gene significances were calculated as the resulting Pearson's correlation coefficient from correlations between gene expression and its relevant module eigengenes (see Supplementary Methods 3 for details on the selection of the most representative module).

### Gene ontology term enrichment analysis

Gene ontology (GO) terms associated with DEGs and WGCNA modules were retrieved from the *E. cicutarium* draft genome annotation files. GO term enrichment analysis was performed with the *topGO* (Alexa and Rahnenführer 2025) R package by comparing the GO terms from all expressed genes with those from the DEG sets, using the weighted Fisher's exact test (FET) statistic. A GO term was considered enriched when the FDR-adjusted value of the weighted FET was less than 0.05. When enriched GO term sets were large enough (hundreds), GO terms and their FDR values were analyzed with REVIGO to improve visualization (Supek et al. 2011), using 0.5 set and UniProtKB as reference protein database.

## Results

### *E. cicutarium* draft genome features

**Fig. 1.**
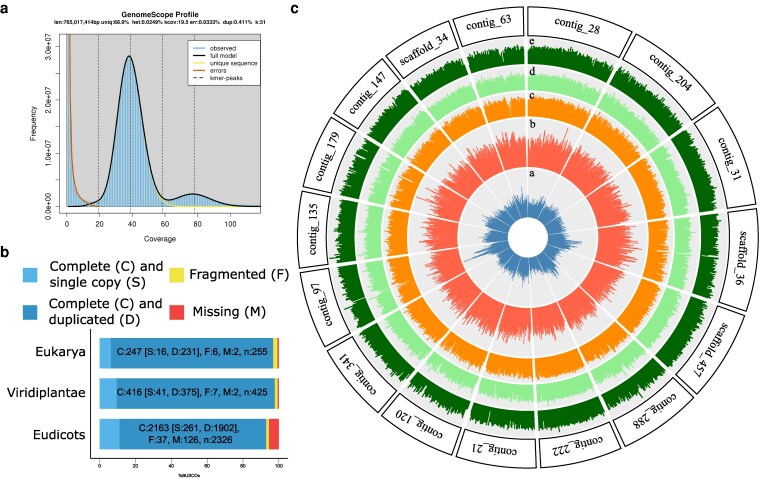
*E. cicutarium* genome assembly quality check. a) GenomeScope plot from filtered Illumina reads for k-mer 31. The *x* axis shows coverage values, and the *y* axis shows coverage distribution frequency for the observed (blue bars) and expected (black line) genome models. Estimated coverage by Illumina reads was 130×. The 2 peaks, the first being higher than the second, indicate polyploidy. The header shows the estimated genome length (len), percentage of unique k-mers (uniq), estimated percentage of heterozygosity (het), mean k-mer coverage (kcov), sequencing error rate (err), and length of the analyzed k-mers (k). b) BUSCO completeness analysis of the genome. The *x* axis shows percentage values, and the *y* axis shows BUSCOs found in the Eukarya, Viridiplantae, and Eudicots databases. Colored bars show the number of BUSCOs found in each search: single copy complete (light blue), duplicated complete (dark blue), fragmented (yellow), and missing (red). c) Circos graph of the genome. The outer ring shows the contigs with a length of 6 Mbps or more (*n* = 16), inner rings show repeated element (a) and gene (b) density in blue and red, respectively, and RNA-seq fragment coverage of juvenile roots (c), juvenile leaves (d), and adult leaves (e) in orange, light green and dark green, respectively.

Illumina sequencing yielded 100 Gbp from 333.20 million paired-end 150 bp reads, and PacBio sequencing yielded a total of 131.55 Gbp from 6.75 million long reads. Both Illumina and PacBio reads can be found in the NCBI's sequence read archive (SRA), under BioProject accession PRJNA984161. The assembly was built with an estimated sequencing coverage of 163.68×. GenomeScope inferred a genome sequence length of 765.02 Mbp (Fig. 1a) and produced a profile compatible with diploid and allotetraploid genomes (Ranallo-Benavidez et al. 2020). The reference genome assembly for *E. cicutarium* ended up with a total length of 801.44 Mbp (∼5% longer than predicted by GenomeScope), distributed in 1,206 contigs, with a contig maximum length of 9.13 Mbp and a N50 of 1.95 Mbp. Genome assembly's average GC content was 41%, and the repeated elements (REs) content in the assembly was 41.97%. Most contigs and scaffolds show a high frequency of genic regions, showing evenly distributed RNA-seq coverage along the contigs, together with a lower frequency of repetitive elements. The raw reads are hosted in the sequence read archive (SRA) from NCBI, under accession number SRR28842229. The genome sequence is hosted in the genome repository of NCBI, under accession number JBDFRG000000000.

The total number of inferred gene models was 186,428, with 172,514 showing an open reading frame. Genome completion assessed through BUSCO score casted a 92.99% of complete sequences against the *eudicot_obd10* database (accessed on 2023 February 2, Fig. 1b), with a proportion of duplicated complete BUSCOS of 87.93%; a similar trend was found when comparing against Viridiplantae and Eukarya databases. Figure 1c shows contigs longer than 6 Mbp with repeated elements and gene densities and RNA-seq expression of the samples. Genome annotation predicted a total of 22 DNA methyltransferase genes involved in the establishment and maintenance of DNA methylation and 10 genes involved in active DNA demethylation (all of them annotated as DEMETER-like protein 1). Note that none of the genes involved in active DNA demethylation were actually found to be differentially expressed in any of the comparisons (results not shown). Thus, in the following sections, we will present results exclusively on DNA methyltransferases.

### DNA methyltransferase sequence analysis

**Fig. 2.**
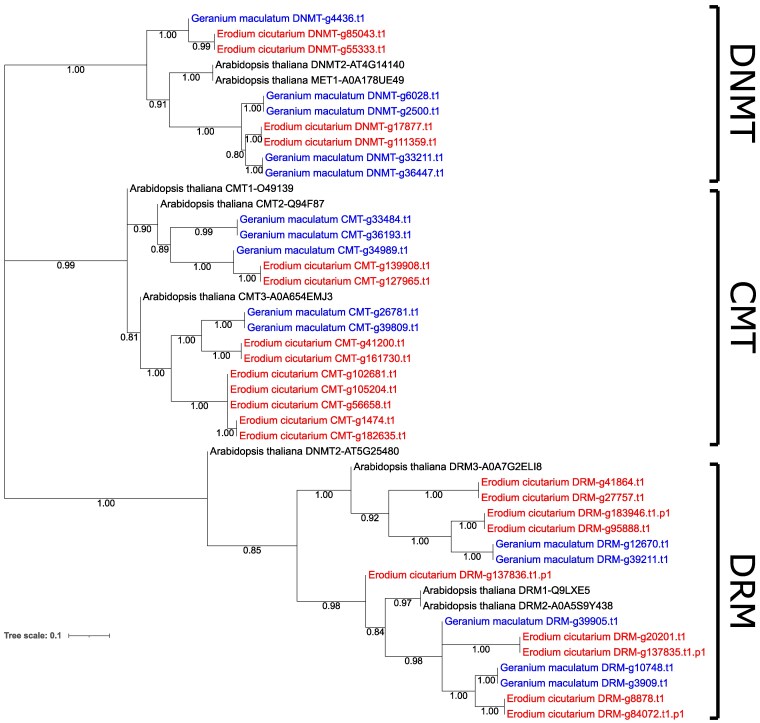
Phylogenetic tree of DNA methyltransferases from the *E. cicutarium* genome. The consensus neighbor-joining tree was generated with MEGA v12.0.10 from an alignment between the amino acid sequences of the 22 DNA methyltransferases annotated in the *E. cicutarium* (red) draft genome, 15 from the *G. maculatum* genome protein set (blue), and those retrieved from UniProtKB for *A. thaliana* (black) DNA methyltransferases. The scale bar denotes a length of 0.1 substitutions per nucleotide site. Bootstrap branch supports are shown in each node. The 3 main DNA methyltransferase clades (DNA methyltransferases *DNMT*, chromomethylases *CMT*, and domains rearranged methyltransferases *DRM*) are marked with delimitation bars in the right part of the tree.

Of the total of 22 DNA methyltransferase genes predicted, 9 were annotated as chromomethylases (*CMT*), 4 DNA methyltransferase 2 (*DNMT2*), and 9 domain rearranged DNA methyltransferases (*DRMs*). BLASTp search retrieved 15 homologous sequences (5 *CMTs*, 5 *DNMTs*, and 5 *DRMs*) from the *G. maculatum* genome's protein set. Although no *MET1* homologous sequence was found in *E. cicutarium* nor *G. maculatum* genomes, analysis of both Geraniaceae and *A. thaliana* DNA methyltransferase sequences revealed that a clade including four *E. cicutarium* sequences annotated as *DNMTs* and five *G. maculatum DNMT* sequences was as closely related to *A. thaliana MET1* as to the *DNMT2* with accession AT4G14140 (Fig. 2). The phylogeny also shows a loss of the *CMT1* clade and a diversification of clade CMT3 in both Geraniaceae. Alignment of amino acid sequences also confirmed the presence of the 6 conserved catalytic domains described so far for plants (Pavlopoulou and Kossida 2007) in the *E. cicutarium* DNA methyltransferase gene set (Supplementary Fig. 1).

### RNA sequencing and mapping to genome

A total of 1,356 million pair-end reads of 151 bps length were produced by the Illumina RNA sequencing experiment (*N* = 35 samples; Table 2). After trimming, 1,332.6 million pair-end reads remained, with lengths ranging from 32 to 151 bps. Mean read mapping rate to the *E. cicutarium* draft genome was 83.8%, ranging from 73.3% to 87.0%. After fragment count summarizing and expression value filtering, 70,973 gene models were considered for differential gene expression analysis. Table 2 shows all the sequencing statistics per library.

**Table 2.** *E. cicutarium* RNA-seq library statistics. Columns show plant ID, sample ID (labeled after the individual), Sequencing Read Archive (SRA) accession number, seed treatment, tissue, developmental stage (age), million sequences in the raw library, million sequences after trimming, and the percentage of reads aligned to the *E. cicutarium* genome.

Plant ID	Sample ID	Accession	Treatment	Tissue	Age	M seq (raw)	M seq (trim)	% Aligned
ECI1	ecic01_C_L_Y	SRR29751979	Control	Leaf	Juvenile	39.8	38.6	77.20
ECI1	ecic01_C_R_Y	SRR29751978	Control	Root	Juvenile	42	41.2	84.95
ECI2	ecic02_A_L_Y	SRR29751967	5-azaC	Leaf	Juvenile	35.8	35	85.71
ECI2	ecic02_A_R_Y	SRR29751963	5-azaC	Root	Juvenile	26.8	26.2	84.73
ECI3	ecic03_C_L_Y	SRR29751962	Control	Leaf	Juvenile	39.4	38.2	73.30
ECI3	ecic03_C_R_Y	SRR29751961	Control	Root	Juvenile	31.8	30.8	82.47
ECI4	ecic04_A_L_Y	SRR29751960	5-azaC	Leaf	Juvenile	31.4	30.8	85.71
ECI4	ecic04_A_R_Y	SRR29751959	5-azaC	Root	Juvenile	34.6	34	84.71
ECI5	ecic05_C_L_Y	SRR29751958	Control	Leaf	Juvenile	34.8	34.2	84.21
ECI6	ecic06_A_L_Y	SRR29751957	5-azaC	Leaf	Juvenile	28	27.6	84.78
ECI6	ecic06_A_R_Y	SRR29751977	5-azaC	Root	Juvenile	37.4	36.6	84.15
ECI7	ecic07_C_L_Y	SRR29751976	Control	Leaf	Juvenile	40.2	39.4	82.23
ECI7	ecic07_C_R_Y	SRR29751975	Control	Root	Juvenile	39	38.6	86.01
ECI8	ecic08_A_L_Y	SRR29751974	5-azaC	Leaf	Juvenile	40.6	39.8	86.43
ECI8	ecic08_A_R_Y	SRR29751973	5-azaC	Root	Juvenile	42	41.2	85.44
ECI9	ecic09_C_L_Y	SRR29751972	Control	Leaf	Juvenile	41.8	41.4	84.54
ECI9	ecic09_C_R_Y	SRR29751971	Control	Root	Juvenile	41.8	41	81.46
ECI10	ecic10_A_L_Y	SRR29751970	5-azaC	Leaf	Juvenile	38	37	80.54
ECI10	ecic10_A_R_Y	SRR29751969	5-azaC	Root	Juvenile	42.2	41.2	83.01
ECI11	ecic11_C_L_Y	SRR29751968	Control	Leaf	Juvenile	34	33	82.42
ECI11	ecic11_C_R_Y	SRR29751966	Control	Root	Juvenile	34.8	33.8	84.62
ECI12	ecic12_A_L_Y	SRR29751965	5-azaC	Leaf	Juvenile	39	38	85.79
ECI12	ecic12_A_R_Y	SRR29751964	5-azaC	Root	Juvenile	36.8	36	85.56
E1	ecic1_A_L_O	SRR29751797	5-azaC	Leaf	Adult	42	41.6	82.21
E12	ecic12_C_L_O	SRR29751828	Control	Leaf	Adult	41.8	41.4	86.96
E13	ecic13_A_L_O	SRR29751827	5-azaC	Leaf	Adult	41.8	41.4	86.96
E14	ecic14_C_L_O	SRR29751816	Control	Leaf	Adult	41.8	41.4	83.57
E17	ecic17_A_L_O	SRR29751805	5-azaC	Leaf	Adult	42.2	41.8	83.73
E18	ecic18_C_L_O	SRR29751798	Control	Leaf	Adult	42	41.6	84.13
E2	ecic2_C_L_O	SRR29751793	Control	Leaf	Adult	42	41.6	83.65
E21	ecic21_A_L_O	SRR29751796	5-azaC	Leaf	Adult	42	41.6	86.06
E24	ecic24_C_L_O	SRR29751795	Control	Leaf	Adult	42.2	41.8	84.69
E29	ecic29_A_L_O	SRR29751794	5-azaC	Leaf	Adult	42	41.6	83.65
E5	ecic5_A_L_O	SRR29751813	5-azaC	Leaf	Adult	41.8	41.4	83.57
E8	ecic8_C_L_O	SRR29751799	Control	Leaf	Adult	42.2	41.8	85.65

### Differential gene expression analysis

DESeq2 normalization managed to standardize the median of the fragment counts (Supplementary Fig. 2a). Principal component analysis (PCA) of the top 500 most expressed genes across samples predicted strong effects of tissue and age in gene expression, and not so outstanding divergence for the comparison between control and 5-azaC-treated plants that was the main aim of our experiment (Supplementary Fig. 2b). As expected, there were tens of thousands of DEGs between juvenile roots and juvenile leaves (54,424, Supplementary Table 1) and juvenile and adult leaves (37,102, Supplementary Table 2). For brevity, here, we will not comment on more details on the vast differences in gene expression between the 2 tissues (leaves vs roots) or developmental stages (juvenile vs adult leaves) in *E. cicutarium* (but see also Supplementary Fig. 3 and Supplementary Results 1), and in the following paragraphs, we will focus principally on gene expression divergences associated with 5-azaC seed treatment.

When all samples and tissues were compared together, DESeq2 detected 164 DEGs for the control and 5-azaC comparison (147 upregulated and 17 downregulated by 5-azaC; Supplementary Table 3). Among the 31 DEGs that were annotated, a chromomethylase *CMT1* homologous gene (involved in non-CpG methylation maintenance) and 3 *MYB* transcription factors (involved in stomatal development, jasmonate and cytokinins signaling pathways, and other processes) were upregulated by 5-azaC, as well as some genes related to the anthocyanin synthesis pathway (*B-MYB*, UDP-glycosyltransferase 84B1). The hypergeometric test showed that the overlap of the control vs 5-azaC DEG set was not greater than expected by chance in any case (*P* > 0.05).

**Fig. 3.**
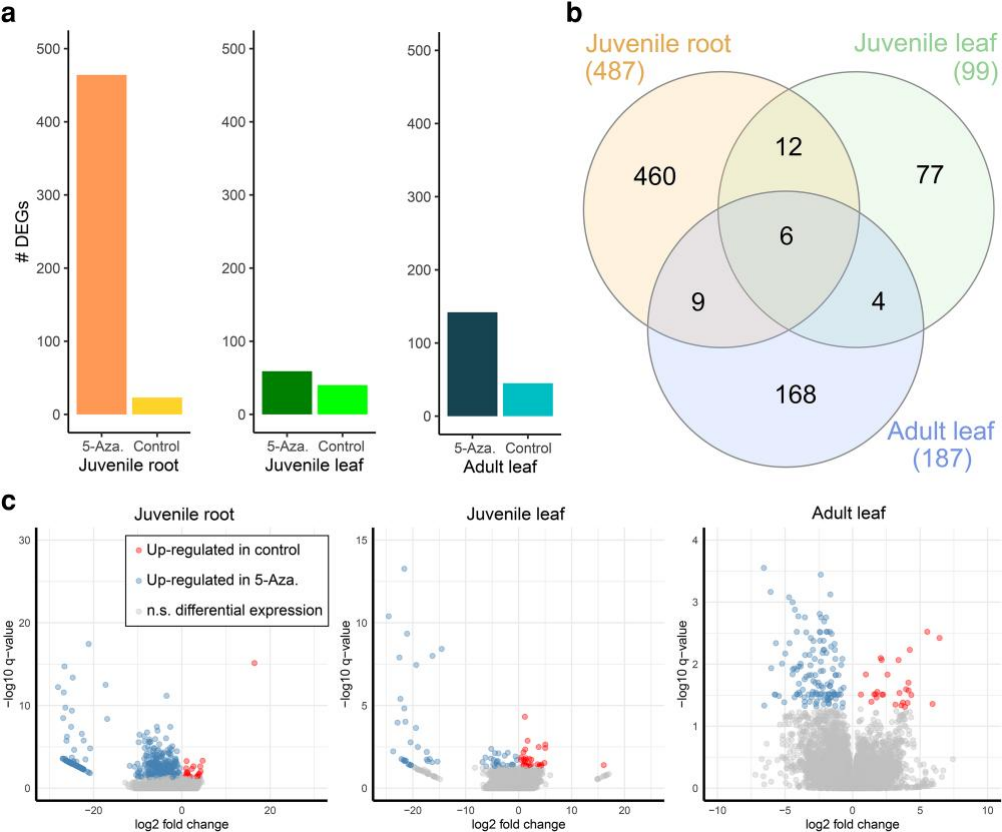
Differential gene expression analysis of 5-azaC treatment within tissue and age samples of *E. cicutarium*. a) number of differentially expressed genes (DEGs) between control and 5-azaC within young leaves, young roots, and adult leaves. The *x* axis shows all tested pairs of groups, and the *y* axis shows the number of DEGs upregulated in each group. b) Venn diagram showing the number of overlapping DEGs per group comparison. Each circle represents a comparison between 2 groups, and numbers inside the circles show how many DEGs are in each overlapping subset. Numbers in parentheses show the total number of DEGs found in the comparison. c) Volcano plots of each tested comparison (specified in the header of each plot). The *x* axis shows the logarithm to the base 2 of the fold change, and the *y* axis shows the negative logarithm to the base 10 of the false discovery rate. Blue and red dots represent genes downregulated (blue) or upregulated (red) by 5-azaC in each comparison, while gray dots are genes not differentially expressed in the comparison.

Differential gene expression analyses of 5-azaC effect within tissue datasets detected 99 and 487 DEGs for juvenile leaves and juvenile roots, respectively (Fig. 3, Supplementary Tables 4 and 5). In juvenile leaves, 59 DEGs were upregulated in the 5-azaC-treated samples and 40 downregulated (including a *histone H3.2* protein, involved in chromatin remodeling). In juvenile roots, 464 DEGs were upregulated in the 5-azaC-treated samples (including a domain rearranged methyltransferase *DRM2* homolog, involved in non-CpG methylation maintenance, and a DNA-directed RNA polymerase 23kD subunit, involved in siRNA synthesis and subsequent RNA-directed DNA methylation) and 23 downregulated. Comparison in adult leaves detected 187 DEGs, 142 upregulated and 45 downregulated by 5-azaC (Fig. 3, Supplementary Table 6). The number of overlapping DEGs between these datasets was still not greater than expected by chance in any case (*P*-value > 0.05), suggesting rather distinct effects of 5-azaC in the 3 sample types here analyzed.

**Fig. 4.**
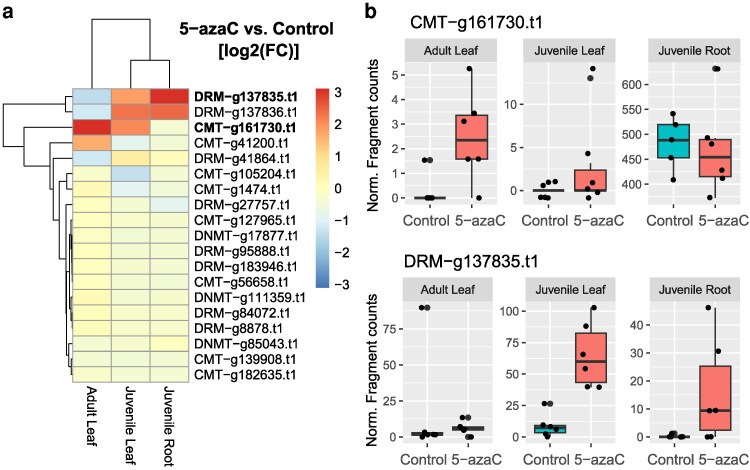
DNA methyltransferase gene expression profiles. a): Heatmap of the DNA methyltransferases with confirmed expression in our data set. Logarithm of the fold change between 5-azaC and control samples within adult leaves, juvenile leaves, and juvenile roots is represented in the tiles of the heatmap, with upregulation in 5-azaC samples taking red tones and downregulation taking blue tones. Hierarchical clustering of the expression is represented with dendrograms. Differentially expressed genes are marked in bold. b) Boxplot showing the variation of gene expression of the 2 differentially expressed genes from the DNA methyltransferase family in all the *E. cicutarium* individuals sampled. The *x* axis shows control (in blue) and 5-azaC (in red) samples, and the *y* axis shows expression levels represented as the normalized fragment counts for both genes in adult leaves, juvenile leaves, and juvenile roots. In both figures each dot denotes an individual sample, the lower and upper boundaries of the boxplot indicate the 25th and 75th percentiles, the horizontal line within the box marks the median, and the whiskers indicate data range.

As regards the DNA methyltransferases, only 2 out of the 19 genes that passed the coverage threshold showed significant differences in expression between control and 5-azaC plants, being overexpressed when treated with 5-azaC (Fig. 4a). First, gene 161730.t1 (homologous to *CMT1*), which was mainly expressed in roots, was detected as upregulated across all 5-azaC-treated samples (FDR = 0.004; Fig. 4b), in part due to the control samples having more expression values equal to zero in the 2 leaf types. Second, gene 137835.t1 (homologous to *DRM2*) was significantly upregulated in 5-azaC-treated roots (FDR = 0.006; Fig. 4b) and showed also higher expression values in juvenile leaves treated with 5-azaC compared to controls, although the test was not statistically significant for this comparison in juvenile leaves (FDR = 0.373). Noticeably, both genes showed a higher variation in expression across samples treated with 5-azaC compared to controls (Fig. 4b).

### Gene ontology term enrichment analysis

A total of 16,072 GO terms were associated with ca. 42,000 annotated genes (out of ca. 71,000 gene models) included in the differential gene expression analysis. The DEG set from the 5-azaC general effect comparison did not show significantly enriched GO terms. In contrast, the DEG sets from the within-tissue and developmental stage comparisons showed more variable outputs, with an overall trend for enrichment in the sets from upregulated genes in 5-azaC-treated samples. In particular, in the control vs 5-azaC comparison for juvenile leaves, no GO terms were enriched. In juvenile roots, DNA integration (GO:0015074) and another 4 GO terms linked to retrotransposon activity were enriched in the DEG set from roots treated with 5-azaC (Supplementary Fig. 4a, Supplementary Table 7). In adult leaves, the DEG set from samples treated with 5-azaC showed nine enriched GO terms, including 2 related to release from seed dormancy (GO:0048838, GO:1902039), positive regulation of the gibberellic acid-mediated signaling pathway (GO:0009939), response to osmotic stress (GO:0006970), and other functions associated with membrane transport and photo inhibition (Supplementary Fig. 4b, Supplementary Table 7). DEG sets from control upregulated in juvenile root and adult leaf samples did not show enriched GO terms.

As a reference for comparison, we explored the functional categories differentially represented between tissues and developmental stages categories. We found a total of 1,072 GO terms enriched between leaves and roots at the juvenile stage (Supplementary Table 8) whereas 1,295 GO terms were enriched in the comparison between leaves collected at juvenile vs adult reproductive stage (Supplementary Table 9).

### Gene co-expression network analysis

**Fig. 5.**
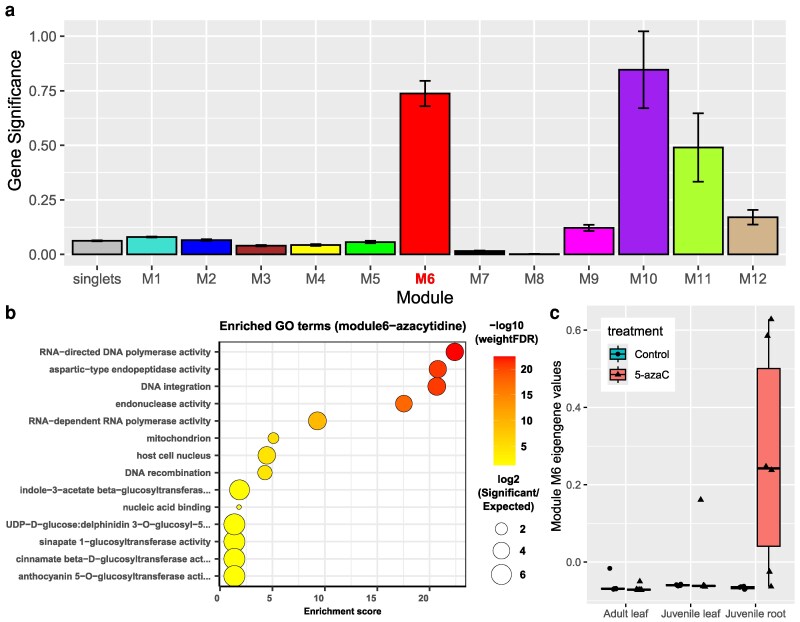
Gene co-expression network analysis and 5-azaC-associated module M6 description. a) gene significances by module. The *x* axis shows the module names, and the *y* axis shows the gene significance (negative logarithm to the base 10 of the false discovery rate) values. Bars show the mean gene significance value per module, with error bars showing the standard errors. b) Enriched GO terms in genes from module M6. The *x* axis shows the enrichment score calculated by *topGO*, and the *y* axis shows the names of the enriched GO terms. Circles show the logarithm to the base 2 of the ratio between the observed and expected GO term counts (circle size) and the negative logarithm to the base 10 of the false discovery rate value (circle color). c) Eigengene values from module M6. The *x* axis shows each sample type, and the *y* axis shows the MEs. Each box shows percentiles 25, 50, and 75 of its respective group (with 5-azaC-treated samples in red and control samples in blue).

A total of 57,411 genes were included in 1 of the 12 generated gene co-expressed modules (Supplementary Fig. 5a, Supplementary Table 10). PCA of module eigengenes (MEs, Supplementary Table 11) showed a similar clustering to the PCA with expression data, samples being separated by age and tissue rather than discriminating those differing in 5-azaC treatment (Supplementary Fig. 5b). Module 10 was excluded from this analysis due to excess of genes with a fragment count of zero (see Supplementary Methods 3 for the details on this decision). Thus, module M6 showed the highest gene significance values when considering control vs 5-azaC adjusted *P*-values (Fig. 5a), being particularly associated with increased expression in samples treated with 5-azaC. It was enriched with GO terms related to retrotransposon activity and anthocyanin metabolism (Fig. 5b, Supplementary Table 12). For the most specific control vs 5-azaC comparisons conducted for each sample type, we found that the highest gene significance values for the juvenile roots was also held by module M6, whereas juvenile leaves and adult leaves comparisons were most associated with modules M9 and M12, respectively (Supplementary Fig. 6). Finally, the 2 differentially expressed DNA methyltransferase genes, *CMT1* and *DRM2*, were included in modules M1 and M2, respectively; both modules showed MEs upregulated in roots, independently of their 5-azaC treatment.

## Discussion

Cytidine analogs such as 5-azaC have been established as relevant experimental tools for understanding DNA demethylation in model and nonmodel plant species (see Dvořák Tomaštíková and Pecinka 2025 for a recent review). Combined with another environmental factor of interest including biotic interactions or abiotic conditions, they provide a tractable strategy to detect direct links between epigenetic regulation and functional phenotypic responses in plant species with contrasting life histories or ecological features, contributing, thus, to the rising interest in ecological epigenetics (Balao et al. 2018; Alonso et al. 2019b; Williams et al. 2025). Thus, understanding how 5-azaC affects gene expression and gene regulatory networks in different plant species is key for an accurate interpretation of its multiple phenotypic effects and potential contribution to uncover the epigenetic contribution to plant adaptation in heterogeneous and stressful environments. In the following paragraphs, we will discuss the mRNA transcription effects recorded in this study aimed to understand developmental and tissue-specific changes in the response to seed demethylation in a single inbred line of *E. cicutarium*. Noteworthy, heterogeneous phenotypic and methylation responses after foliar application of 5-azaC have been previously described for *Quercus lobata* seedlings with different provenances (Browne et al. 2020) and also after seed treatment in two ecotypes of *Thlaspi arvense* (Troyee et al. 2022) and 2 populations of *E. cicutarium* (Alonso et al. 2026). Thus, our study illustrates a baseline for transcriptomic changes after seed demethylation with 5-azaC within a homogenous genetic background.

Exposure to 5-azaC can be toxic for plants (Qiu et al. 2010; Grzybkowska et al. 2018; Osorio-Montalvo et al. 2020); thus, adjusting the treatment concentration is critical to prevent increased mortality or dwarfism (Alonso et al. 2017 and references therein). Our study uses a 500 µM concentration, which is in the range of other studies that did not intend to generate toxic effects on treated plants but were proven to significantly reduce DNA methylation (Troyee et al. 2022; Alonso et al. 2026). In fact, gene expression differences related to tissue and developmental stage overwhelm those related to 5-azaC, which increased individual variation in gene expression, rather than affecting it in a directional or unimodal way. Signs of stress response in the transcriptome did show only in adult leaves treated with 5-azaC, where GO terms from photoinhibition and osmotic stress were enriched. This trend may be a direct consequence of the 5-azaC toxicity or an indirect effect due to trade-offs induced by phenotype changes, but since the juvenile stage did not show such stress response in this study, the latter seems more plausible.

Previous studies showed that 5-azaC treatment at seed stage successfully dropped global DNA methylation in *E. cicutarium* roots of adult plants but did affect more strongly to leaves of juvenile than adult individuals (Alonso et al. 2026). Still, many DNA methylation changes were observed in adult leaves, which recorded a higher reduction of methylation in CG sequences and similar numbers of methylation gains and losses in cytosines at other sequence contexts (Balao et al. 2024).

Several plant studies have shown how DNA methyltransferase genes are upregulated by 5-azaC. In 8-week-old leaves of *A. thaliana*, resulting hypomethylation from exposure to 5-azaC induced the upregulation of DNA methyltransferases *MET1* (a member of the *DNMT* group), *CMT3*, *DRM1*, and *DRM2*, together with other genes involved in the DNA repair pathway (Kiselev et al. 2019). Calluses of *Citrus paradisi*, when exposed to 5-azaC, showed increased levels of *MET1* and *CMT1* transcripts (Xu et al. 2017). On the other hand, DNA methyltransferases *CMT3*, *MET1*, and *DRM2* were downregulated when exposed to 5-azaC in *Paeonia suffruticosa* (Zhang et al. 2020). In our study, we found *CMT1* upregulated by 5-azaC, which might point toward alternative compensation mechanisms when DNA methylation in CG positions is inhibited. In addition, *DRM2* (involved in de novo cytosine methylation through the small RNA-dependent DNA methylation pathway) and a *RdDM* polymerase component were also upregulated in roots treated with 5-azaC, suggesting that the plant-specific *RdDM* pathway may be enhanced after 5-azaC treatment. *CMT1* and *DRM2* promote and maintain non-CpG methylation (Cao et al. 2003) and are more sensitive to environmental cues (Du et al. 2012; Bouyer et al. 2017), which may allow the treated plants some environmentally dependent responsivity for DNA methylation regulation. Our finding that expression levels of *DNMT* genes were unaffected by 5-azaC treatment could be related to the mechanism of action of 5-azaC, which primarily binds covalently to *DNMT* proteins, inactivating them, without affecting *DNMT* transcript levels (Christman 2002; Stresemann and Lyko 2008).

Roots of nonreproductive juvenile plants were particularly susceptible regarding their transcriptome response to 5-azaC exposure: they not only showed more upregulated DEGs by 5-azaC compared to leaves coming from either juveniles or reproductive adults, but they also showed a strong association with module M6 of the gene co-expression network. Both DEGs in roots treated with 5-azaC and genes in module M6 contained gene sets enriched with transposable elements. It is well established that 5-azaC treatment upregulates transposon expression in other plants (Hudson et al. 2011, Griffin et al. 2016; Xu et al. 2017). Module M6 was also enriched in genes related to the metabolic pathway of anthocyanins, compounds responsible for red colors in plants, but also related to plant stress response (Gould 2004, Landi et al. 2015). Anthocyanins seem to be regulated by DNA methylation in several plants, mostly due to overlap between a transposable element and the promoter of *MYB* family transcription factors (Wang et al. 2020, Zhu et al. 2020, Li et al. 2022). Given that transposable elements are heavily methylated in plants to repress their expression while sometimes overlapping with promoter regions (Ito et al. 2011, Matzke and Mosher 2014), finding co-expression between transposons and anthocyanin signaling pathway genes may indicate that DNA methylated transposable elements play a potential role in anthocyanin gene expression regulation. Our results suggest that those changes would be relevant not only above ground (eg fruit maturation, leaf abiotic stress, and herbivory responses) but also in roots, where they could respond to defense-growth trade-offs. Since the sampled adult developmental stage individuals were part of a longer experiment intended to explore phenotypic and reproductive consequences of experimental demethylation, we could not collect root tissues of adult plants to avoid exposing them to further stress, and consequently we were unable to fully understand the temporal dynamics of 5-azaC-induced gene expression changes of this tissue.

Given *E. cicutarium*'s estimated genome size (765 Mbps, 6-fold bigger than *A. thaliana*) and its possible polyploidy, each replication event should have incorporated higher than expected amounts of 5-azaC during the 48 h exposure; thus, we expected a fast depletion of 5-azaC within cells and a consequential dissipation of its effects during life time. In contrast, adult leaves showed more DEGs induced by 5-azaC compared to juvenile leaves. Moreover, the overlap between DEGs affected by 5-azaC in juvenile and adult leaves was not greater than expected by chance, suggesting that the long-term response to DNA demethylation by 5-azaC affects different pathways than the short-term response, which is a new finding that should be investigated in other species to better understand long-term responses to stress. Among the differentially expressed genes from adult leaves, we found that 5-azaC induced upregulation of genes related to seed dormancy inhibition and the gibberellic acid signaling pathway, the latter being also a contributor to seed dormancy release (Nonogaki 2014). At least another study found that the transcriptome of *P. suffruticosa* plants exposed to 5-azacytidine also shows upregulation of seed dormancy release genes (Zhang et al. 2020). Further, changes in flowering timing in plants exposed to 5-azaC are associated with the DNA demethylation of regulatory elements from genes belonging to the gibberellic acid biosynthetic pathway (Burn et al. 1993; Kondo et al. 2007; Yari et al. 2021). In *E. cicutarium*, 5-azaC delayed flowering and growth (Alonso et al. 2026), a response that could be associated with positive regulation of the gibberellic acid biosynthesis pathway by 5-azaC in leaves collected when plants were blooming, but not at the earliest juvenile stage.

In conclusion, by characterizing both the transcriptomic response to 5-azaC and the DNA methyltransferase gene set from *E. cicutarium*, this study expands the available genomic resources for nonmodel species, a need to move forward current understanding of the epigenetic contribution for adaptation in wild plant populations. In particular, we found a significant transcriptomic response to 5-azaC treatment in all study tissues and developmental stages, which differed in strength between the 3 sample types analyzed and with very few DEGs shared among them. Contrary to our expectations, the response in leaves was stronger at adult than juvenile stage, supporting the use of seed demethylation in studies aimed to investigate epigenetic transgenerational effects in plant adaptation. Finally, the analysis of expression of DNA methyltransferases, with an upregulation of *CMT1* and *DRM2* by 5-azaC, pointed toward activation of alternative compensation mechanisms when DNA methylation in CG positions is inhibited that could also involve genes associated with transposable elements. In future studies, tracking down plant phenotype changes after 5-azaC treatment can be extended by the study of whole-transcriptome RNA-seq (including noncoding RNAs), chromatin accessibility profiling (eg ATAC-seq), and methylome, which allows to view the integrative response of plants to 5-azaC, potentially enhancing the identification of which phenotype changes are caused by changes in DNA methylation or solely by gene expression changes. Given the impact of 5-azaC seed treatment in adult reproductive plants, such experiments could be performed at a longer-term range.

## Supplementary Material

jkag129_Supplementary_Data

## Data Availability

The *E. cicutarium* genomic data are publicly available at the NCBI genome repository under genome project accession JBDFRG000000000, with the raw reads hosted at the NCBI Sequence Read Archive (SRA) repository under accession number SRR28842229. Protein sequences used in the phylogenetic analysis are available at Supplementary Methods 2 in this journal. RNA-seq raw reads are available at the NCBI SRA repository under accession numbers SRR29751957-SRR29751979. Scripts to analyze the data are publicly available at GitHub (https://github.com/rmblazquez/erodium-cicutarium-Transcriptomics-ebd). All Supplementary Figures 1 to 6, Supplementary Methods 1 to 3, Supplementary Results 1 and Supplementary Tables 1 to 12 are available at https://doi.org/10.25387/g3.30621302.
