# Supplementary material for: Understanding epigenetic regulation in nonmodel plants: transcriptomic responses to seed demethylation in leaves and roots of the annual herb Erodium cicutarium (Geraniaceae)
Source: G3 (Bethesda). 2026 May 15;16(8):jkag129. doi: 10.1093/g3journal/jkag129 (PMC13439943; doi:10.1093/g3journal/jkag129)
Supplement: Supplementary file 1 [file jkag129_supplementary_data.zip › Supplemental_Material_G3-2026-406718.pdf]

Supplementary material from "Understanding epigenetic regulation in non-model plants: Transcriptomic responses to seed demethylation in leaves and roots of the annual herb *Erodium cicutarium* (Geraniaceae)"

Martín-Blázquez, Rubén; Medrano, Mónica; Alonso, Conchita

Estación Biológica de Doñana, CSIC, Avenida Américo Vespucio 26, Sevilla 41092, Spain.

This document contains all **Supplementary Figures** (**S1-S6**, p2-p7) referred within the main text and in depth description of methodological details regarding the *Erodium cicutarium* genome assembly (**Supplementary Methods S1**, p8), the sequences of all DNA methyl transferase proteins used for phylogenetic analysis (**Supplementary Methods S2**, p11), the criteria used for representative gene module selection (**Supplementary Methods S3**, p26), further information on the differences in gene expression between the two tissues (leaves *vs*. roots) and developmental stages (juvenile *vs*. adult leaves) investigated in *E. cicutarium* (**Supplementary Results S1**, p.28), and the **List of references** cited here (p29). Finally, all **Supplementary Tables** (Table S1 to Table S12) quoted here or in the main text are provided in another Excel File whose link is available in the Supplementary Data section of this article, in the G3 Journal's web site.

Index

Supplementary Figures S1-S6 page 2

Supplementary Methods S1 page 8

Supplementary Methods S2 page 11

Supplementary Methods S3 page 26

Supplementary Results S1 page 28

List of references page 29

Supplementary Figures

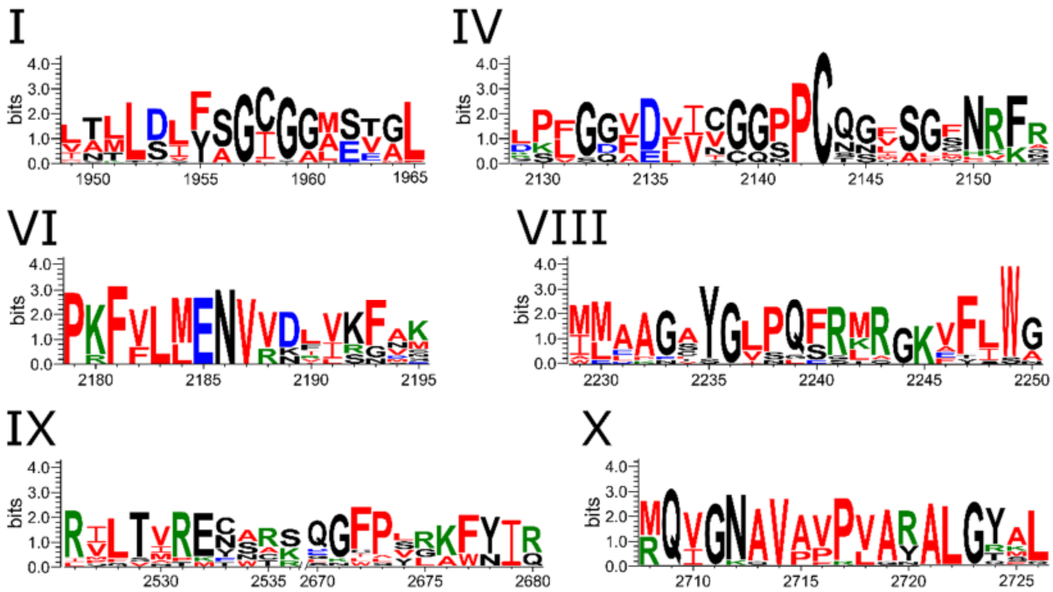

Supplementary Fig. S1. Sequence logo of the catalytic sites of the 22 *E. cicutarium* DNA methyltransferases. The six logos are based on the alignment of the DNA methyltransferase amino acid sequences exclusively from *E. cicutarium*, and represent conserved patterns (in roman numerals) from the catalytic site of the enzyme, based on Pavlopoulou and Kossida 2007. X-axis shows the position of each residue in the alignment, Y-axis shows the bit-score of each position, black letters show the amino acid residues found per alignment position, with their size being proportional to its frequency, and colors being used to highlight each aminoacid chemical properties (black: polar, blue: acidic, green: basic, red: hydrophobic).

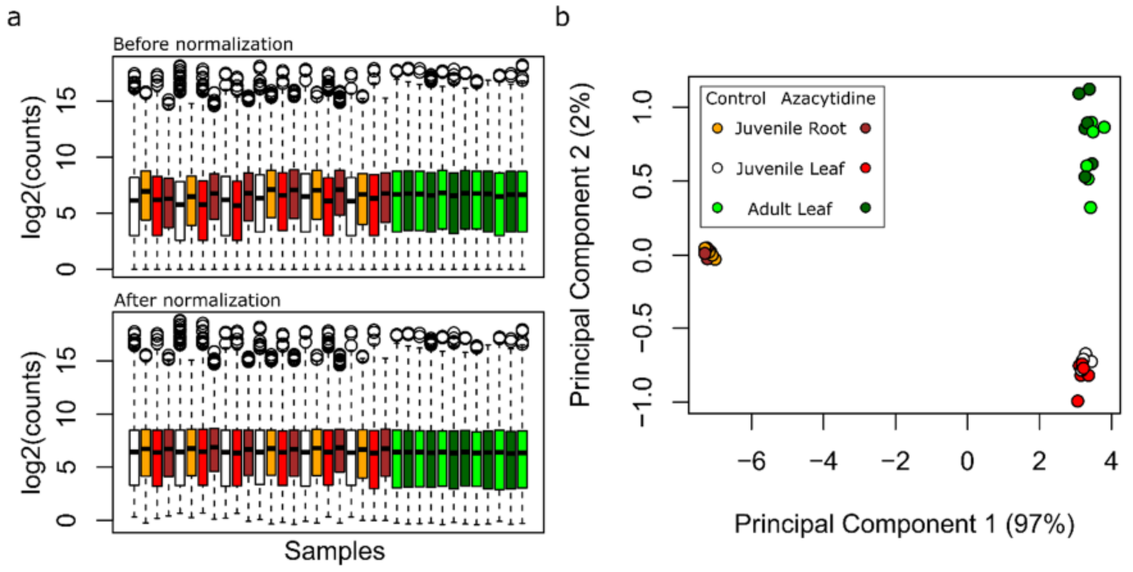

Supplementary Fig. S2. RNA-seq normalization and sample grouping. **a**: comparison of fragment counts per RNA-seq library before (top) and after (bottom) DESeq2 normalization method. X-axis shows the sample (color coded), Y-axis shows the logarithm to the base two of the fragment counts. **b**: Biplot of the principal components PC1 (X-axis) and PC2 (Y-axis) of the top 500 most expressed genes. Samples are color-coded. Percentage of explained variance per PC is shown in parentheses.

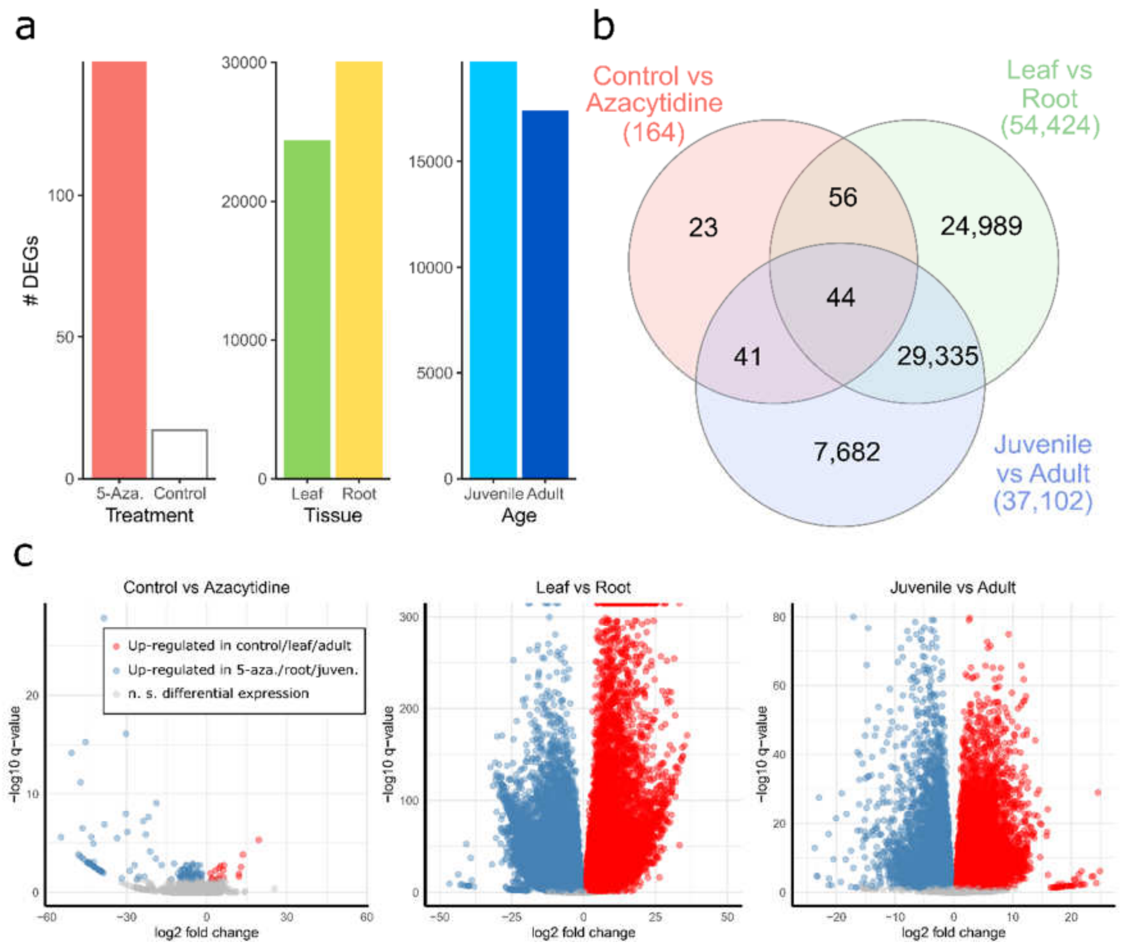

Supplementary Fig. S3. Differential gene expression analysis between treatment, tissue and age groups. **a**: number of differentially expressed genes (DEGs) in treatment (control vs. 5-azacytidine), tissue (leaf vs. root), and age (juvenile vs. adult) comparisons. X-axis shows all tested pairs of groups, Y-axis show the number of DEGs up-regulated in each group, note that Y-axis scale is different for each comparison. **b**: Venn diagram showing the number of overlapping DEGs per group comparison. Each circle represents a comparison between two groups, numbers inside circles show how many DEGs are in each overlapping subset. Numbers in parentheses show the total number of DEGs found in the comparison. **c**: Volcano plots of each tested comparison (specified in the header of each plot). X-axis shows the logarithm to the base 2 of the fold change, Y-axis shows the negative logarithm to the base 10 of the false discovery rate, blue and red dots represent genes whose are up-regulated in the specified groups (legend), while grey dots are genes not differentially expressed in the comparison.

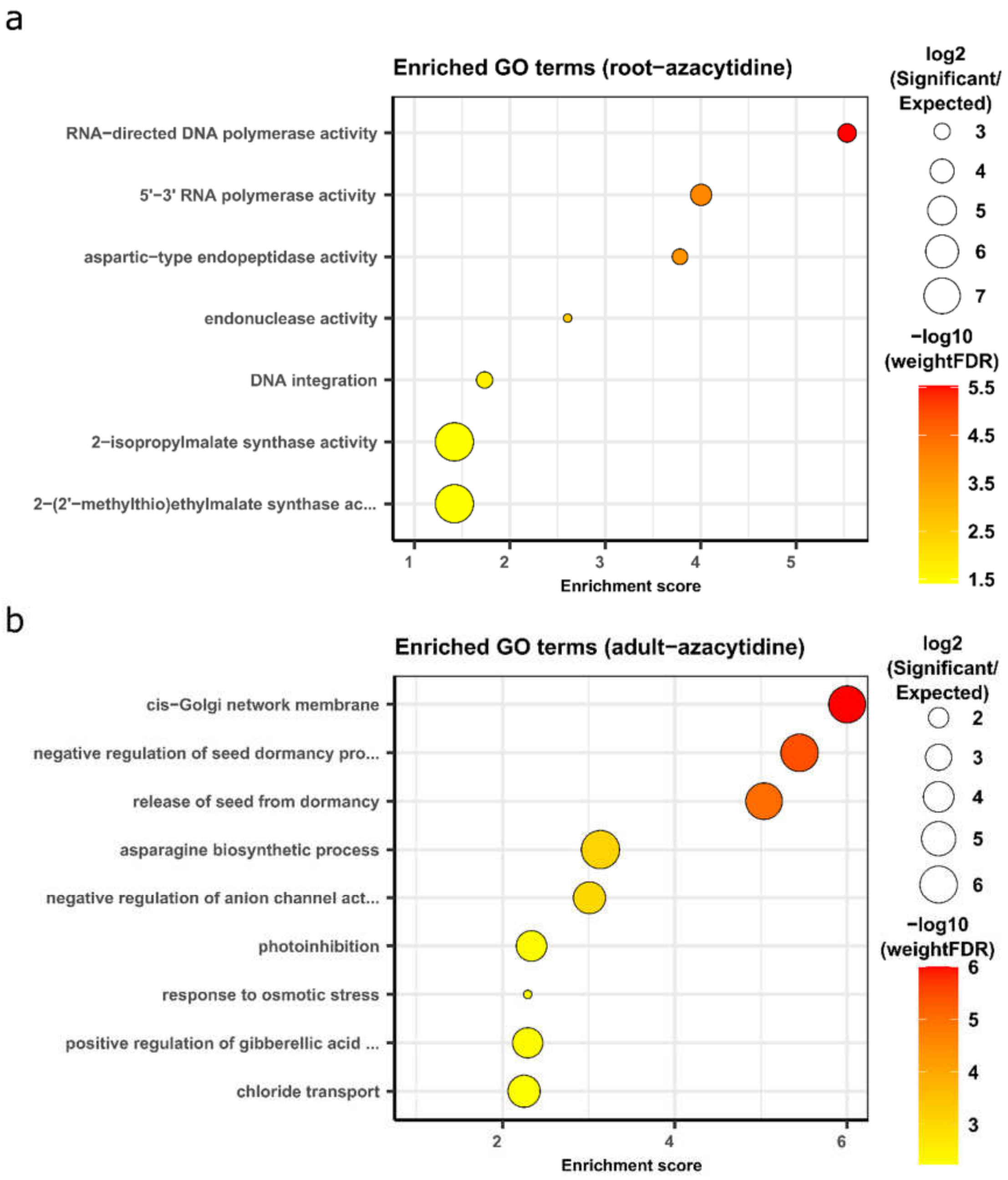

Supplementary Fig. S4. Enriched GO terms in genes up-regulated by 5-azacytidine. The plots show statistically significant enriched GO terms found in DEG sets up-regulated by 5-azacytidine in juvenile roots (**a**) and adult leaves (**b**). X-axis shows the enrichment score calculated by topGO, Y-axis shows the names of the enriched GO terms. Circles show the logarithm to the base two of the ratio between the observed and expected GO term counts (circle size) and the negative logarithm to the base 10 of the false discovery rate value (circle color).

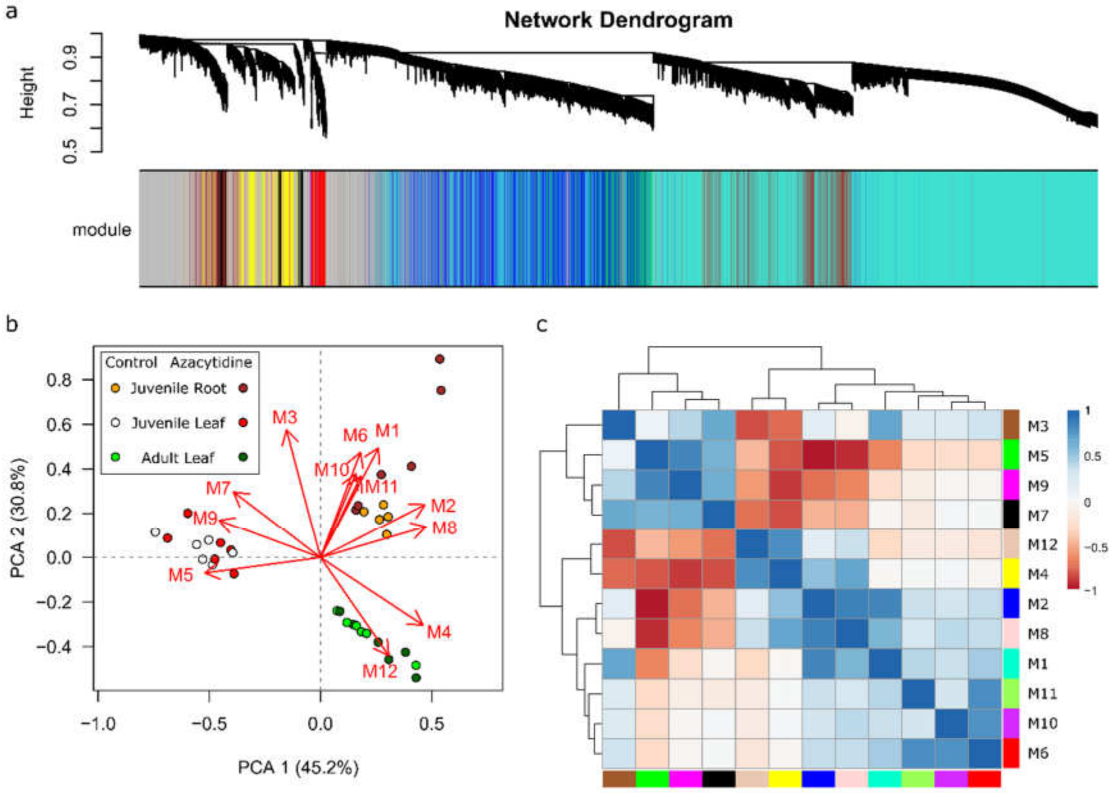

Supplementary Fig. S5. Gene co-expression network. **a**: Dendrogram of the network produced by WGCNA. Y-axis show the node “height” (distance values), branches represent Euclidean distances between genes, which are the nodes at the tip of each branch. The bar box in X-axis shows the module assignation of each gene (color coded, grey bars indicate no module assignation). **b**: Triplot of PCs one and two from the principal component analysis of the module eigengenes (MEs). The triplot shows samples (color coded dots) and module associations (red arrows). Percentages of explained variance are in parentheses. **c**: Correlogram between modules. Each tile of the heatmap shows Pearson’s correlation coefficient *r* between the MEs of two modules (blue: positive *r*, red: negative *r*). Dendrograms represent module grouping through hierarchical clustering according to their *r* pair-wise values.

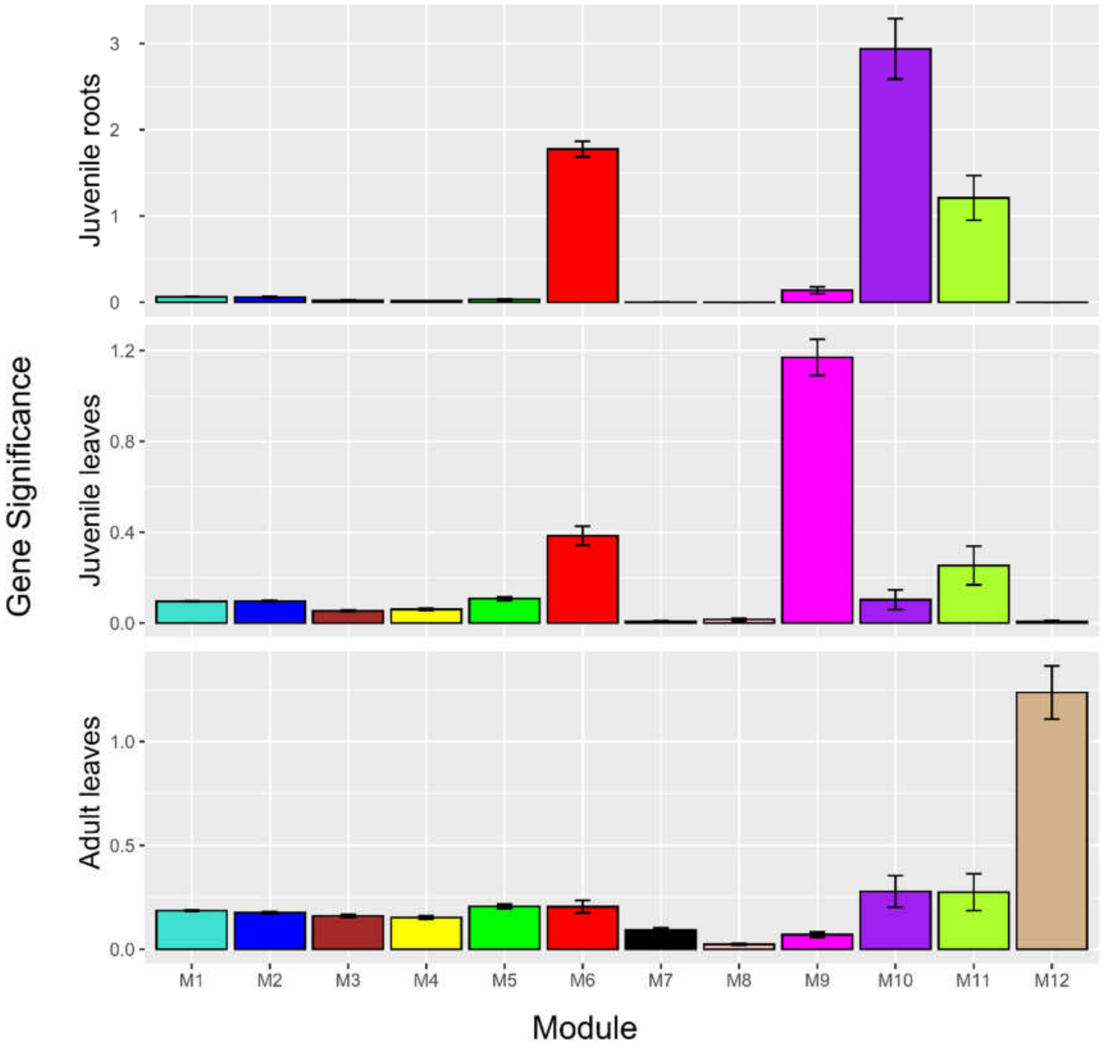

Supplementary Fig. S6. Gene significance values per module in control vs. 5-azacytidine comparisons. Each plot shows the gene significance values for each module, when considering control and 5-azacytidine comparisons for either juvenile roots, juvenile leaves, and adult leaves. X-axis show the module name. Y-axis show the gene significance value range. Bars represent gene significance mean values per module (colored), with error bars representing standard errors.

Supplementary Methods S1. *Erodium cicutarium* genome assembly.

DNA was isolated from leaves of a single *E. cicutarium* individual, the F4 generation of a self-pollinated lineage (strain *F0-CH34_F3-Ecic127_F4-01*). Library preparation and sequencing were performed by AllGenetics (www.allgenetics.eu). Illumina paired-end short reads library were constructed using the HiSeq X Reagent Kit v2.5, sequenced in a HiSeq X PE150 (Illumina) with an Illumina patterned flow cell, and then quality-checked using the software FastQC v0.11.5 (Andrews 2010). PacBio long reads library was constructed using SMRTbell Express Template Prep Kit 2.0 (PacBio), sequenced with a Sequel II sequencer (PacBio), with an SMRT Cell 8M under the Long-reads mode, and quality-checked with SequelTools (Hufnagel et al. 2020).

Short and long reads were assembled *de novo* into "mega-reads" using the software MaSuRCA v3.4.2 (Zimin et al. 2013) using Flye v2.5 algorythm (Kolmogorov et al. 2019), with the short reads being error-corrected using QuorUM (Marcais et al. 2015). The generated assembly was polished using POLCA (Zimin and Salzberg 2020). Quality check of the assembly was performed with QUAST 5.0.2 (Gurevich et al. 2013). The program Jellyfish (Marcais and Kingsford 2011) was used on the quality checked Illumina paired-end reads (333,195,590 reads of length 150 x 2 bp) to count the occurrences of k-mers in the corrected reads setting the k-mer size parameter to k = 31. GenomeScope (Vurture et al. 2017) was used to plot the k-mer profile.

BlobTools v1.1.1 (Laetsch and Blaxter 2017) was used to detect potential contaminant sequences in the assembly. The quality and completeness of the genome assembly was evaluated using BUSCO V5.beta.1 (Simão et al. 2015, Manni et al. 2021), setting the "*genome*" mode, using *eudicots_odb10, viridiplantae_odb10, and eukarya_odb10* (last updated 2020-09-10) as reference databases.

RepeatModeler v2.0.1 (Flynn et al. 2020) was used to *de novo* identify repetitive elements, and RepeatMasker v4.1.2-p1 (Smit et al. 2021) was used to mask repeated elements from the assembly, using the Repbase-20170127 library as reference database.

AUGUSTUS v3.2.3 (Stanke et al. 2006) was used for gene prediction in the resulting assembly, using the options 'species = *Arabidopsis thaliana*' and 'gff = on'. Softwares gffread v0.12.6 (Pertea and Pertea 2020) and TransDecoder v5.5.0 (Haas et al. 2013) were used to consolidate transcript sequences and to identify candidate open reading frames within the transcripts.

The predicted protein-coding genes were subsequently functionally annotated using InterProScan v5.50-84.0 (Jones et al. 2014), using several reference databases summarized in **List A** (see below). An additional annotation step was performed using the software Sma3s v2 (Munoz-Merida et al. 2014), using Swiss-Prot database from UniProtKB as reference (Consortium 2007).

The Pac-Bio long reads are hosted in the sequence read archive (SRA) from NCBI, under accession number SRR28842229. The genome sequence is hosted in the genome repository of NCBI, under accession number JBDFRG000000000.

List A. Databases used for the annotation of *E. cicutarium* genome.

Database name	Reference
Coils	Lupas A, Van Dyke M, Stock J. 1991. Predicting Coiled Coils from Protein Sequences. *Science.* 252(5009): 1162-1164.
Conserved Domain Database (CDD)	Lu S et al. 2020. CDD/SPARCLE: the conserved domain database in 2020. *Nucleic Acids Res.* 48(D1): D265-D268.
Gene3D	Lewis TE, Sillitoe I, Dawson N, Lam SD, Clarke T, Lee D, Orengo C, Lees J. 2018. Gene3D: Extensive prediction of globular domains in proteins. *Nucleic Acids Res.* 46(D1): D435-D439.
HAMAP	Pedruzzi I et al. 2015. HAMAP in 2015: updates to the protein family classification and annotation system. *Nucleic Acids Res.* 43(Database issue): D1064-1070.

MobiDBLite	Necci M, Piovesan D, Dosztanyi Z, Tosatto SCE. 2017. MobiDB-lite: fast and highly specific consensus prediction of intrinsic disorder in proteins. *Bioinformatics.* 33(9): 1402-1404.
Protein Analysis THrough Evolutionary Relationships (PANTHER)	Mi H, Muruganujan A, Thomas PD. 2013. PANTHER in 2013: modeling the evolution of gene function, and other gene attributes, in the context of phylogenetic trees. *Nucleic Acids Res.* 41(Database issue): D377-386.
Protein domains, families and functional sites (ProSiteFamilies and ProSiteProfiles)	Sigrist CJ, de Castro E, Cerutti L, Cuche BA, Hulo N, Bridge A, Bougueleret L, Xenarios I. 2013. New and continuing developments at PROSITE. *Nucleic Acids Res.* 41(Database issue): D344-347.
Protein families database (PFAM)	Finn RD et al. 2014. Pfam: the protein families database. *Nucleic Acids Res.* 42(Database issue): D222-230.
Protein information resource and superfamily (PIRSF)	Wu CH et al. 2004. PIRSF: family classification system at the Protein Information Resource. *Nucleic Acids Res.* 32(Database issue): D112-114.
Protein motifs fingerprints (PRINTS)	Attwood TK, Beck ME, Bleasby AJ, Parry-Smith DJ. 1994. PRINTS a database of protein motif fingerprints. *Nucleic Acids Res.* 22(17): 3590-3596.
Simpler modular architecture research tools (SMART)	Letunic I, Bork P. 2018. 20 years of the SMART protein domain annotation resource. *Nucleic Acids Res.* 46(D1): D493-D496.
Structure function linkage (SFLD)	Akiva E et al. 2014. The Structure-Function Linkage Database. *Nucleic Acids Res.* 42(Database issue): D521-530.
SUPERFAMILY	Gough J, Karplus K, Hughey R, Chothia C. 2001. Assignment of homology to genome sequences using a library of hidden Markov models that represent all proteins of known structure. *J Mol Biol.* 313(4): 903-919.
TIGRFAM	Haft DH, Selengut JD, Richter RA, Harkins D, Basu MK, Beck E. 2013. TIGRFAMs and Genome Properties in 2013. *Nucleic Acids Res.* 41(Database issue): D387-395.

Supplementary Methods S2. DNA methyl transferase protein sequences.

The following DNA methyltransferases, chromomethylases, and domain rearranged DNA methyltransferases protein sequences were used in phylogenetic analysis:

```
>Arabidopsis_thaliana_MET1-A0A178UE49
MVENGAKAAKRKKRPLPEIQEVEDVPRTRRPRRAAACTSFKEKSIRVCEKSATIEVKKQQIVEEEFLALR
LTALETDVEDRPTRRLNDFVLFDSDGVPQPLEMLEIHDIFVSGAILPSDVCTDKEKEKGVRCTSFGRVEH
WSISGYEDGSPVIWISTELADYDCRKPAASYRKVYDYFYEKARASVAVYKKLSKSSGGDPDIGLEELLAA
VVRSMSSGSKYFSSGAAIIDFVISQGDFIYNQLAGLDETAKKHESSYVEIPVLVALREKSSKIDKPLQRE
RNPSNGVRIKEVSQVAESEALTSDQLVDGTDDDRRYAILLQDEENRKSMQQPRKNSSSGSASNMFYIKIN
EDEIANDYPLPSYYKTSEEETDELILYDASYEVQSEHLPHRMLHNWALYNSDLRFISLELLPMKQCDDID
VNIFGSGVVTDDNGSWISLNDPDSGSQSHDPDGMCIFLSQIKEWMIEFGSDDIISISIRTDVAWYRLGKP
SKLYAPWWKPVLKTARVGISILTFLRVESRVARLSFADVTKRLSGLQANDKAYISSDPLAVERYLVVHGQ
IILQLFAVYPDDNVKRCPFVVGLASKLEDRHHTKWIIKKKKISLKELNLNPRAGMAPVASKRKAMQATTT
RLVNRIWGEFYSNYSPEDPLQATAAENGEDEVEEEGGNGEEEVEEEGENGLTEDTVPEPVEVQKPHTPKK
IRGSSGKREIKWDGESLGKTSAGEPLYQQALVGGEMVAVGGAVTLEVDDPDEMPAIYFVEYMFESTDHCK
MLHGRFLQRGSMTVLGNAANERELFLTNECMTTQLKDIKGVASFEIRSRPWGHQYRKKNITADKLDWARA
LERKVKDLPTEYYCKSLYSPERGGFFSLPLSDIGRSSGFCTSCKIREDEEKRSTIKLNVSKTGFFINGIE
YSVEDFVYVNPDSIGGLKEGSKTSFKSGRNIGLRAYVVCQLLEIVPKESRKADLGSFDVKVRRFYRPEDV
SAEKAYASDIQELYFSQDTVVLPPGALEGKCEVRKKSDMPLSREYPISDHIFFCDLFFDTSKGSLKQLPA
NMKPKFSTIKDDTLLRKKKGKGVESEIESEIVKPVEPPKEIRLATLDIFAGCGGLSHGLKKAGVSDAKWA
IEYEEPAGQAFKQNHPESTVFVDNCNVILRAIMEKGGDQDDCVSTTEANELAAKLTEEQKSTLPLPGQVD
FINGGPPCQGFSGMNRFNQSSWSKVQCEMILAFLSFADYFRPRYFLLENVRTFVSFNKGQTFQLTLASLL
EMGYQVRFGILEAGAYGVSQSRKRAFIWAAAPEEVLPEWPEPMHVFGVPKLKISLSQGLHYAAVRSTALG
APFRPITVRDTIGDLPSVENGDSRTNKEYKEVAVSWFQKEIRGNTIALTDHICKAMNELNLIRCKLIPTR
PGADWHDLPKRKVTLSDGRVEEMIPFCLPNTAERHNGWKGLYGRLDWQGNFPTSVTDPQPMGKVGMCFHP
EQHRILTVRECARSQGFPDSYEFAGNINHKHRQIGNAVPPPLAFALGRKLKEALHLKKSPQHQP

>Arabidopsis_thaliana_DNMT2-AT4G14140
MEMETKAGKQKKRSVDSDDDVSKERRPKRAAACTNFKEKSLRISDKSETVEAKKEQILAEEIVAIQLTSS
LESNDDPRPNRRLTDFVLHDSEGVPQPVEMLELGDIFIEGVVLPLGDEKKEEKGVRFQSFGRVENWNISG
YEDGSPVIWISTALADYDCRKPSKKYKKLYDYFFEKACACVEVFKSLSKNPDTSLDELLAAVSRSMSGSK
IFSSGGAIQEFVISQGEFIYNQLAGLDETAKNHETCFVENRVLVSLRDHESNKIHKALSNVALRIDESKV
VTSDHLVDGAEDEDVKYAKLIQEEEYRKSMERSRNKRSSTTSGGSSRFYIKISEDEIADDYPLPSYYKNT
KEETDELVLFEAGYEVDTRDLPCRTLHNWTLYNSDSRMISLEVLPMRPCAEIDVTVFGSGVVAEDDGSGF
CLDDSESSTSTQSNDHDGMNIFLSQIKEWMIEFGAEMIFVTLRTDMAWYRLGKPSKQYAPWFGTVMKTVR
VGISIFNMLMRESRVAKLSYANVIKRLCGLEENDKAYISSKLLDVERYVVVHGQIILQLFEEYPDKDIKR
CPFVTSLASKMQDIHHTKWIIKKKKKILQKGKNLNPRAGIAPVVSRMKAMQATTTRLVNRIWGEFYSIYS
PEVPSEAINAENVEEEELEEVEEEDENEEDDPEENELEAVEIQNSPTPKKIKGISEDMEIKWDGEILGKT
SAGEPLYGRAFVGGDVVVVGSAVILEVDDQDDTQLICFVEFMFESSNHSKMLHGKLLQRGSETVLGMAAN
ERELFLTNECLTVQLKDIKGTVSLEIRSRLWGHQYRKENIDVDKLDRARAEERKTNGLPTDYYCKSLYSP
ERGGFFSLPRNDMGLGSGFCSSCKIRENEEERSKTKLNDSKTGFLSNGIEYHNGDFVYVLPNYITKDGLK
KGSRRTTLKCGRNVGLKAFVVCQLLDVIVLEESRKASKASFQVKLTRFYRPEDISEEKAYASDIQELYYS
QDTYILPPEAIQGKCEVRKKSDMPLCREYPILDHIFFCEVFYDSSTGYLKQFPANMKLKFSTIKDETLLR
EKKGKGVETGTSSGMLMKPDEVPKEKPLATLDIFAGCGGLSHGLENAGMYLYSHVMHILLSSKHLKTFIK
MHVLCNKVYLLQSGRSSMKSQLVMRLNKTILKQRFLLTTAMAIMEKCGDVDDCVSTVEAAELAAKLDENQ
KSTLPLPGQVDFINGGPPCQGFSGMNRFSHGSWSKVQCEMILAFLSFADYFRPKYFLLENVKKFVTYNKG
RTFQLTMASLLEMGYQVRFGILEAGTYGVSQPRKRVIIWAASPEEVLPEWPEPMHVFDNPGSKISLPRGL
RYDAGCNTKFGAPFRSITVRDTIGDLPPVENGESKINKEYGTTPASWFQKKIRGNMSVLTDHICKGLNEL
```

NLIRCKKIPKRPGADWRDLPDENVTLSNGLVEKLRPLALSKTAKNHNEWKGLYGRLDWQGNLPISITDPQ
PMGKVGMCFHPEQDRIITVRECARSQGFPDSYEFSGTTKHKHRQIGNAVPPPLAFALGRKLKEALYLKSS
LQHQS

>Arabidopsis_thaliana_DNMT2-AT5G25480
MAEQELQRINEKKPWQVLEFYSGIGGMRYSLMASGIVSEVVEAFEINDSANDVYQHNFKHRPYQGNIQSL
TAADLDKYNADAWLLSPPCQPYTRQGLQKHSGDARAFSFLRILELIPHTTKPPQMLFVENVVGFETSDTH
MEMIGTLTKLDYVTQEFILSPLQFGVPYSRPRYFCLAKRKPLPFKSQHSNNKLLWSPDPLYGRDDQVEFG
KCQAEEGLDKLLEFCKPVEKFLELAAHVDGEPSSVDDSENGSKDCCGQEGDSVPDSVHQYLVPVSLIERW
GNAMDIVYPDSKRCCCFTKSYYRYVKGTGSLLATVQPKIKGKESCLKEQRLRYFTPREVANFHSFPEDFE
FPKHISLRQRYAMLGNSLSVAVVAPLLRYLFDS

>Arabidopsis_thaliana_CMT1-O49139
MAARNKQKKRAEPESDLCFAGKPMSVVESTIRWPHRYQSKKTKLQAPTKKPANKGGKKEDEEIIKQAKCH
FDKALVDGVLINLNDDVYVTGLPGKLKFIAKVIELFEADDGVPYCRFRWYYRPEDTLIERFSHLVQPKRV
FLSNDENDNPLTCIWSKVNIAKVPLPKITSRIEQRVIPPCDYYYDMKYEVPYLNFTSADDGSDASSSLSS
DSALNCFENLHKDEKFLLDLYSGCGAMSTGFCMGASISGVKLITKWSVDINKFACDSLKLNHPETEVRNE
AAEDFLALLKEWKRLCEKFSLVSSTEPVESISELEDEEVEENDDIDEASTGAELEPGEFEVEKFLGIMFG
DPQGTGEKTLQLMVRWKGYNSSYDTWEPYSGLGNCKEKLKEYVIDGFKSHLLPLPGTVYTVCGGPPCQGI
SGYNRYRNNEAPLEDQKNQQLLVFLDIIDFLKPNYVLMENVVDLLRFSKGFLARHAVASFVAMNYQTRLG
MMAAGSYGLPQLRNRVFLWAAQPSEKLPPYPLPTHEVAKKFNTPKEFKDLQVGRIQMEFLKLDNALTLAD
AISDLPPVTNYVANDVMDYNDAAPKTEFENFISLKRSETLLPAFGGDPTRRLFDHQPLVLGDDDLERVSY
IPKQKGANYRDMPGVLVHNNKAEINPRFRAKLKSGKNVVPAYAISFIKGKSKKPFGRLWGDEIVNTVVTR
AEPHNQCVIHPMQNRVLSVRENARLQGFPDCYKLCGTIKEKYIQVGNAVAVPVGVALGYAFGMASQGLTD
DEPVIKLPFKYPECMQAKDQI

>Arabidopsis_thaliana_CMT2-Q94F87
MLSPAKCESEEAQAPLDLHSSSRSEPECLSLVLWCPNPEEAAPSSTRELIKLPDNGEMSLRRSTTLNCNS
PEENGGEGRVSQRKSSRGKSQPLLMLTNGCQLRRSPRFRALHANFDNVCSVPVTKGGVSQRKFSRGKSQP
LLTLTNGCQLRRSPRFRAVDGNFDSVCSVPVTGKFGSRKRKSNSALDKKESSDSEGLTFKDIAVIAKSLE
MEIISECQYKNNVAEGRSRLQDPAKRKVDSDTLLYSSINSSKQSLGSNKRMRRSQRFMKGTENEGEENLG
KSKGKGMSLASCSFRRSTRLSGTVETGNTETLNRRKDCGPALCGAEQVRGTERLVQISKKDHCCEAMKKC
EGDGLVSSKQELLVFPSGCIKKTVNGCRDRTLGKPRSSGLNTDDIHTSSLKISKNDTSNGLTMTTALVEQ
DAMESLLQGKTSACGAADKGKTREMHVNSTVIYLSDSDEPSSIEYLNGDNLTQVESGSALSSGGNEGIVS
LDLNNPTKSTKRKGKRVTRTAVQEQNKRSICFFIGEPLSCEEAQERWRWRYELKERKSKSRGQQSEDDED
KIVANVECHYSQAKVDGHTFSLGDFAYIKGEEEETHVGQIVEFFKTTDGESYFRVQWFYRATDTIMERQA
TNHDKRRLFYSTVMNDNPVDCLISKVTVLQVSPRVGLKPNSIKSDYYFDMEYCVEYSTFQTLRNPKTSEN
KLECCADVVPTESTESILKKKSFSGELPVLDLYSGCGGMSTGLSLGAKISGVDVVTKWAVDQNTAACKSL
KLNHPNTQVRNDAAGDFLQLLKEWDKLCKRYVFNNDQRTDTLRSVNSTKETSGSSSSSDDDSDSEEYEVE
KLVDICFGDHDKTGKNGLKFKVHWKGYRSDEDTWELAEELSNCQDAIREFVTSGFKSKILPLPGRVGVIC
GGPPCQGISGYNRHRNVDSPLNDERNQQIIVFMDIVEYLKPSYVLMENVVDILRMDKGSLGRYALSRLVN
MRYQARLGIMTAGCYGLSQFRSRVFMWGAVPNKNLPPFPLPTHDVIVRYGLPLEFERNVVAYAEGQPRKL
EKALVLKDAISDLPHVSNDEDREKLPYESLPKTDFQRYIRSTKRDLTGSAIDNCNKRTMLLHDHRPFHIN
EDDYARVCQIPKRKGANFRDLPGLIVRNNTVCRDPSMEPVILPSGKPLVPGYVFTFQQGKSKRPFARLWW
DETVPTVLTVPTCHSQALLHPEQDRVLTIRESARLQGFPDYFQFCGTIKERYCQIGNAVAVSVSRALGYS
LGMAFRGLARDEHLIKLPQNFSHSTYPQLQETIPH

>Arabidopsis_thaliana_CMT3-A0A654EMJ3
MAPKRKRPATKDDTTKSIPKPKKRAPKRAKTVKEEPVTVVEEGEKHVARFLDEPIPESEAKSTWPDRYKP
IEVQPPKASSRKKTKDDEKVEIIRARCHYRRAIVDERQIYELNDDAYVQSGEGKDPFICKIIEMFEGANG
KLYFTARWFYRPSDTVMKEFEILIKKKRVFFSEIQDTNELGLLEKKLNILMIPLNENTKETIPATENCDF
FCDMNYFLPYDTFEAIQQETMMAISESSTISSDTDIREGAAAISEIGECSQETEGHKKATLLDLYSGCGA
MSTGLCMGAQLSGLNLVTKWAVDMNAHACKSLQHNHPETNVRNMTAEDFLFLLKEWEKLCIHFSLRNSPN
SEEYANLHGLNNVEDNEDVSEESENEDDGEVFTVDKIVGISFGVPKKLLKRGLYLKVRWLNYDDSHDTWE

PIEGLSNCRGKIEEFVKLGYKSGILPLPGGVDVVCGGPPCQGISGHNRFRNLLDPLEDQKNKQLLVYMNI
VEYLKPKFVLMENVVDMLKMAKGYLARFAVGRLLQMNYQVRNGMMAAGAYGLAQFRLRFFLWGALPSEII
PQFPLPTHDLVHRGNIVKEFQGNIVAYDEGHTVKLADKLLLKDVISDLPAVANSEKRDEITYDKDPTTPF
QKFIRLRKDEASGSQSKSKSKKHVLYDHHPLNLNINDYERVCQVPKRKGANFRDFPGVIVGPGNVVKLEE
GKERVKLESGKTLVPDYALTYVDGKSCKPFGRLWWDEIVPTVVTRAEPHNQVIIHPEQNRVLSIRENARL
QGFPDDYKLFGPPKQKYIQVGNAVAVPVAKALGYALGTAFQGLAVGKDPLLTLPEGFAFMKPTLPSELA

>Arabidopsis_thaliana_DRM1-Q9LXE5
MVMSHIFLISQIQEVEHGDSDDVNWNTDDDELAIDNFQFSPSPVHISATSPNSIQNRISDETVASFVEMG
FSTQMIARAIEETAGANMEPMMILETLFNYSASTEASSSKSKVINHFIAMGFPEEHVIKAMQEHGDEDVG
EITNALLTYAEVDKLRESEDMNININDDDDDNLYSLSSDDEEDELNNSSNEDRILQALIKMGYLREDAAI
AIERCGEDASMEEVVDFICAAQMARQFDEIYAEPDKKELMNNNKKRRTYTETPRKPNTDQLISLPKEMIG
FGVPNHPGLMMHRPVPIPDIARGPPFFYYENVAMTPKGVWAKISSHLYDIVPEFVDSKHFCAAARKRGYI
HNLPIQNRFQIQPPQHNTIQEAFPLTKRWWPSWDGRTKLNCLLTCIASSRLTEKIREALERYDGETPLDV
QKWVMYECKKWNLVWVGKNKLAPLDADEMEKLLGFPRDHTRGGGISTTDRYKSLGNSFQVDTVAYHLSVL
KPLFPNGINVLSLFTGIGGGEVALHRLQIKMNVVVSVEISDANRNILRSFWEQTNQKGILREFKDVQKLD
DNTIERLMDEYGGFDLVIGGSPCNNLAGGNRHHRVGLGGEHSSLFFDYCRILEAVRRKARHMRR

>Arabidopsis_thaliana_DRM2-A0A5S9Y438
MVIWNNDDDDFLEIDNFQSSPRSSPIHAMQCRVENLAGVAVTTSSLSSPTETTDLVQMGFSDEVFATLFD
MGFPVEMISRAIKETGPNVETSVIIDTISKYSSDCEAGSSKSKAIDHFLAMGFDEEKVVKAIQEHGEDNM
EAIANALLSCPEAKKLPAAVEEEDGIDWSSSDDDTNYTDMLNSDDEKDPNSNENGSKIRSLVKMGFSELE
ASLAVERCGENVDIAELTDFLCAAQMAREFSEFYTEHEEQKPRHNIKKRRFESKGEPRSSVDDEPIRLPN
PMIGFGVPNEPGLITHRSLPELARGPPFFYYENVALTPKGVWETISRHLFEIPPEFVDSKYFCVAARKRG
YIHNLPINNRFQIQPPPKYTIHDAFPLSKRWWPEWDKRTKLNCILTCTGSAQLTNRIRVALEPYNEEPEP
PKHVQRYVIDQCKKWNLVWVGKNKAAPLEPDEMESILGFPKNHTRGGGMSRTERFKSLGNSFQVDTVAYH
LSVLKPIFPHGINVLSLFTGIGGGEVALHRLQIKMKLVVSVEISKVNRNILKDFWEQTNQTGELIEFSDI
QHLTNDTIEGLMEKYGGFDLVIGGSPCNNLAGGNRVSRVGLEGDQSSLFFEYCRILEVVRARMRGS

>Arabidopsis_thaliana_DRM3-A0A7G2ELI8
MADMRRRNGSGGSSNHERNEQILFPKPETLDFDLPCDTSFPQQIGDNAASSSGSNVKSLLIEMGFCPTLV
QKAIDENGQDDFELLLEILTKSTETEPPGPSFHGLMEPKPEPDIEYETDRIRIALLTMKFPENLVDFALD
RLGKDTPIDEMVDFIVAAQLAEKYAEESEDSLDGAEINEEDEDVTPVTARGPEVPNEQLFETMDKTLRLL
EMGFSNDEISMAIEKIGTKGQISVLAESIVTGEFPAECHDDLEDIEKKVSAAAPAVNRTCLSKSWRFVGV
GAQKEDGGGGSSSGTANIKPDPGIESFPFPATDNVGETSRGKRPKDEDENAYPEEYTGYDDRGKRLRPED
MGDSSSFMETPWMQDEWKDNTYEFPSVMQPRLSQSLGPKVARRPYFFYGQLGELSPSWWSKISGFLFGIH
PEHVDTRLCSALRRTEGYLHNLPTVNRFNTLPNPRLTIQDAMPHMRSWWPQWDIRKHFNSGTCSNMKDAT
LLCERIGRRIAECKGKPTQQDQTLILRHCHTSNLIWIAPNILSPLEPEHLECIMGYPMNHTNIGGGRLAE
RLKLFDYCFQTDTLGYHLSVLKSMFPQGLTVLSLFSGIGGAEIALDRLGIHLKGVVSVESCGLSRNILKR
WWQTSGQTGELVQIEEIKSLTAKRLETLMQRFGGFDFVICQNPSTPLDLSKEISNSEACEFDYTLFNEFA
RVTKRVRDMM

>Erodium_cicutarium_DRM3-g27757.t1
EQDKRLMCQACQEVPRAQHASERDPLLLLGDNRPCHIGRTLSAVSITMLFLTRFFTFAAAVSHAPPPGLD
RLSDQAYWPVPIVLVFLGTEAHFLLKTSVVLFLQNDGTKQSLVLGARGQFSFLQMGFTGNQVYAAFDKLG
PNVPISELADSILTGRIADDYIHKEKDCGMGHSPYGLARVKPEDSFQDDVSLSRDMNRGSGREGKTPKQE
CYNLTRKRPKQEYLDDSNSFLRNTWPGENNLDNKPTSYELSTPFKLSASKSIDRVVAKPPYFLYGNVANV
PNESWVRISQFLYAVAPEFVNSKFFSALNRKEGYVHNLPAENRFHIVPKPPLTIEDAIPHTKKWWPSWDT
RKQLSGISSETSGVSQLCDRLGKILNDSQGLLSLLNDSQELLSLEKQKDIVHHCQRLNLVWVGHNKLSPI
EPEHLERILGYPRNHTQYDGSSLKERLHALKHCFQTDTLGYHLSVLKGMYPDGLAMLSVFSGIGGAEVAL
HRLGIRLKGVVSVEMCLRKRRIMKRWWENTEQRGELVQMEDVRELTTNKLKSLMEKLGGFDLVVCQNPCT
SFSLSHVKGADSDGLTGFNLSLFYEFVRVLQRVRNVKESKS

>Erodium_cicutarium_DRM3-g41864.t1

MCQACQGVPRAQHATEPDPLLLLRDNRPCHIGRTLSAVSITVVFFTRFFTLAAAVSHAPPPGLERLPDQA
CTCTDGTKQSLGHGVRGQSSLQQMGFTENQVYAAFDKLGPNVPISELADSILTGRIADDYIHKDKDCGMG
MGDGLIHSPYGLARVKPEDSFQDDVSLSRDMNRGSDLEGKRPKQECYNLTRKRPKQEYLDDFSSFLRNTW
PGENNLDNKPTSYELSTPFNLSPSKSIDRVVAKPPYFLYGNVGNVPNESWVKISQFLYAVAPEFVNAKFF
SALNRKEGYVHNLPAENRFHIVPKPPMTIEDAIPHTKKWWQSWDTRKQLSGISSETRGVSQLCDRLGKIL
NDSQGLLSLLNDSQELLSLEKQKDILHHCQRLNLVWVGHHKLSPIEPEHLERILGYPRNHTHDDGSSLTE
RLQALKHCFQTDTLGYHLSVLKGMYPDGLTMLSVFSGIGGAEVALHRLGVRLKGVVSVEMCLRKRRIMKR
WWENTEQRGELVQMEDVRELTTNKLKSLMDKLGGFDLVVCQNPCTSFSLGHVKGGDSDGLTGFNLSLFYE
FVRVLQRVRNVKESKS

>Erodium_cicutarium_DRM3-g95888.t1
MQCVVLCKLNLLGFTCFNMGEIPGSKSLLDHEVERAIAPKAEPEILDFVLPSQSMWSRGVGDNAASSSGS
NLRSSLIGMGFLPLHVDKAIEEKGEDDADLLVDTLVAYSDLHKSQAESSDSLDGLFDNKGASSPPEVSTV
EPKEEPDVFDDISEDKRASLLMMNFSVNDVDFAIDKLGEDTPIDEMVDFIVAAQMAKKYEKEVEDSTRGN
EDVDEKLYGTMDKTLRLLQMGFTENQVSAAIEKFGSDVPISELADSIFAGQIADDYLDKDKNCGSTGDGI
GHHPYGSVRVKTEDLPRDNNEGLNRKGKIPKQESLDDSSDASSLFRVSDFDYTRKGKRPQQEYLDDSSSF
RKTTCVGEKVSRKSTRFEIPPPHKSGPCKSLDRMGGKTTHFLYGNVANISSDSWAKVSQFLYTTEPEFVK
TQFWSALSRKEGYVHNLPTENRFHIDPKPPMTIQDAIPHTKRWWPSWDTRKQLSCINTEISGISQLCDRL
GKILSDSQGLLSTEHQRDILHHCQTLNLVWVGRYKLGLVKPEHLECILGYPLNHTQFPGISVMERLQSLK
HCFQTDTLGYHLSVLKNIYPDGLTMLSIYSGIGGAEVALHHLGIRLKGVVSVETCALKRKILKRWWESTQ
TGELVQFEDIEKLTGSMLRSLSDKLGGFDFVICQSPCSSSSPGPKKAATEIDGIPGFDFSLFHEYVRVLK
LVRGMSVRK

>Erodium_cicutarium_DRM2-g137835.t1.p1
MAARPKKVAGDGLPPEVWTEIQIGVPIKSILRCRSVCKSWYSLISCPNFTAKYSTRNLNKNGNRFLLRLR
DDITKVTSFSSHDKSFMESREHDDLPDTFLDRSCRFTFIEIVGSCNGLICLSKREPMLGNDRDVGRITFL
IWNPTIRKVVELPSRTYSNEHVSCYGFGFDSKTSDYKVVFMAGANVGAVFDLRKGVWRRISARVHRWFVT
ARQKFYLHGAVHWSGSSDADVDDRILLSFNVSDECFREIKMPDCAEFTDNGDDGESYVTTRCVVFDGLIC
AVVWKSGFDDKIDMSEGCSVWAMKEYGVTESWTKMFDIDLQSAQVLPSTHLSPERFCVWENGVLMVHDLE
VEEDDDFREKNDLVSYNAESRKCKRLVSGARTLVEEHDQQQLIVMGGGALLIPNVTLPRNCLIGPGEHAQ
QTSPDSVEVQKRQQPDYKEEETHKQSPGDRGGIRALVPTALLEARRFGSESDKIVGLIVDLELIPIFENL
HLGKTEAYSDMDGKFSDVEDDIDWNTDDELEIANYHVSSSSTLSFPGEQATAGFGEASCVTGPSSSSSSS
SSKSMIHNFLKMGFKEKVITKAMEENGDADPESMLNTLLTYQAIEDSPEEPELIDLDLPDKQHNVYSDYC
SSDYGGLTNEVSYIDIGSEDEISPYLSSKRRKCHNEMFKRKKPVGLETNILSKDDEILHLPNPMIGFGVP
GKLCSRVDRKIPEAAIGPPYFYYENVAFAPKGVWQKISRFLYDIEPEFVDSIHFCAAARKRGYVHNLPIT
NRFPLVPIPHLTIQDALPMTKKWWATWDPRTKLNCIQTCIGSATLTQRIRKALEECGGEPPKHVKKYILD
ECRRWNLVWVGRNKVAPLEPDEIEMLLGFPKNHTRGGASRTMRYKSLGNSFQVLFFLIYEVLALLKHLNL
SAMTKTGSFLYGQEIMFYVSDS

>Erodium_cicutarium_DRM2-g137836.t1.p1
MIIHKVKGSNMILVDTVAYHLSVLKKLYPRGINLLSLFSGIGGAEVALHKLGIPLNNVVSVEICEENRNI
VRSWWEQTNQRGNLIDIADVQTLNAGWLEELMDSFGGFDLVVGGSPCNNLAGSNRVSRDGLEGEHSSLFF
HYFRILDTVKFAMSSRRR

>Erodium_cicutarium_DRM2-g183946.t1.p1
MGEIPGSKSLLDHEVERAIAPKAEPEILDFVLPSESMWSRGVGDNAASSSGSNLRSSLIGMGFSPSLVDK
AIEEKGEDDVDLLVDTLVGYSDLHKSQAESSDSLDGLFDNKGASSPPEVSTVEPKEEPDVFDDISEDKRA
SLLMMNFSVNDVDFAIDKLGEDTPIDEMVDFIVAAQMAKKYEKEVEDSTHGNEDVDETLYGTMDKTLRLL
QMGFTENQVSAAIEKFGSDVPISELADSIFAGQIADDYLDKDKNCGSTGDGIGHHPYGSVRVKTEVLPRD
NNEGLNRKGKIPKQECLDDSSDASSLFRVSDFDYTRKGKRPQQEYLDDSSSFRKTTCVGEKVSRKSTRFE
VPPPHKSGPCKSLDRMGGTYFLYGNVANISSDSWAKVSQFLNAIEPEFVKTQFLSALSRKEGYVHNLPRE
NRFHIDPKPPMTIQDAIPHTKRWWPSWDTRKQLSCINTEISGISQLCDRLGKILSDSQGLLSTEHQRDIL
HHCQTLNLVWVGRYKLGLVEPHYLECILGYPLNHTQFPEISSMERLQSLKHCFQTDTLGYHLSVLKNIYP
DGLTMLSIFSGIGGAEVALHHLGIRLKGVVSVETCALKRKILKRWWESTQTGELVQFEDIEKLTSSRLQS

LSDKLGGFDFVICQSPCSSSSPGLKKAATEIDGIPGFDFSLFYEFVRVLRLVRGMSPRALTLEKTWFCET
DLGFIMVSVSESVPSTEDTALLREVYGVPLEGLAGKETSSGKEPTEEIAVATEDRGELVGSPMCFAATRG
ASVQEARGLATFRRQQDLLPAYRRGPLRWSPTIGPRSRKNRDAGARGQDGEAFQWGTELSGHMEAAAAER
FEFTPDPNTARLFPPEDGDTVVSREILQEHRLSLSEEELGPPVVQHCLPVEKDAVTWKRRPGKEPARATQ
VERSPNGLGLTHDARRAWTGHQPGPKYGVGPMSGPCEPISGWRSGLGPNLRVGSGLPLVNRETSARIGSS
GPGSGGRGSNFRFALAAKVLTPRTFYWKVMADRMEDLWGMRKIEAKPLEDGVVAFFFKSAKERARVLAGE
PWAFWDSLIIFKYLRLHMRSHDNMTFTHFSVWARVSNLPVGAWNKQVAFRVGNALGQFQGVDYDAAGRCW
GDFMRIRIRMDIRVPLQREMWFRLSESQATCYRHARWEGSDVILLFGPRLTLDRPSRRRNMGKLLRGLIG
HIGGCVRDSISGQRSNDGQREGWEGSGHGRRDFEPWNHLEEVGVDRQQETGEETDDGVPGEERESHGNLP
AQSGGLKRQIGEMKESEGSHSDGASYAAVGGKHPRLEK

>Erodium_cicutarium_DRM2-g20201.t1
MKMSSQIVSVLVRDGNFSDVEDDIDWNTDDELEIANYHVPSSSTLSFPGEQATAGFGEASCVTGPSSSSS
SSKSMIHDFLKMGFKEKVITKAMEENGDADPESMLNTLLTYQAIEDSPEEQELIDLDLPDKQKTVYSDYC
SSDYGGLTNEVSYIDIGSEDEISPYLSSKRRKCHNEMFKRKRPMGLETNILSKDDEILHLPNPMIGFGVP
GKLCSRVNRKIPEAAIGPPYFYYENVAFAPKGVWQKISRFLYDIEPEFVDSIHFCAAARKRGYVHNLPIT
NRFPLVPIPHLTIQDALPMTKKWWATWDPRTKLNCIQTGIGSATLTQRIRKALEECDGEPPKHVKKYVLD
ECRRWNLVWVGRNKVAPLEPDEIEMLLGFPKNHTRGGASRTVRYKSLGNSFQVDTVAYHLSVLKKLYPRG
INLLSLFSGIGGAEVALHKLGIPLNNVVSVEICEENRNIVRSWWEQTNQKGNLIDIADVQTLNAGWLEEL
MDSFSGFDLVVGGSPCNNLAGSNSVSRDGLEGEHSALFFHYVRILDIVKFAMSSRRR

>Erodium_cicutarium_DRM2-g84072.t1.p1
MKQYLTVHQDCGIMGIGKGCYTRIGTSEYSPEWRRGRVYESLLFRSLSYSDDAFSSHLFARFLPRFHLHV
ADCILLFEEAVEREVFEGGTLSVEEKIWKITRYLVYYQLTEDGNVPSGENDIDWNTDDELEIANYAVSST
SSLSLPGAEETGEASCTAGPSNSMVRDFLEMGFSEKMIARAIEENGEGDGESILNTLLTYQAIENSPERE
QPIDTDIPTEQQTVGSDRCSSDYGSFEDSSDIDSFSENEEFHASSTDIQKIKEEIIDMSSETNHNLLFLV
DMGFTMVEATIAIQRCGPDSSVDELADFIGAARLAKAADANMVDLPEVKPTSYLGSKRRKHLNGDSLKRK
KIRGLNEDEEAIRLPNPMIGFGIPGELCPVINRSLPEAAIGPPFFYYENVALAPRGVWNTISRFLYDVEP
EFVDSKYFCAAARKRGYVHNLPVVNRYPLVPLPPRTIHDALPLTRKWWPSWDTRTKLNCLQTCIASAKLT
DRIRMALEDWEGDPPLHVQKYVLNECRKWNLVWVGRNKLAPLEPDEVEMLLGFPKNHTRGGGISRTDRYK
SLGNSFQVDTVAYHLSVLKEIFPGGINMLSLFSGIGGAEVALHRLGIPLKNVVSVDISKQNRNIVRDWWE
QTNQKGNLIDIADVQTLNADRLEQLMSSFGGFDLVVGGSPCNNLAGSNRHHRDGLEGKQSSLFYDYFRIL
DVVKGVMTKGM

>Erodium_cicutarium_DRM1-g8878.t1
MDGNVPSGEDDIDWNTDDELEIANYAVSSTSSLSLPGAEETGEASCTAGPSNSMVHDFLEMGFSEKMIAR
AMEENGEGDGESILNTLLTYQAIENSPEQEQPIDTDIPTEQQTVGSDRCSSDYGSFEDSSDIDSFSENEE
FHASSTDIQKIKEEIIDMSSETNHNLLFLVDMGFTMVEATIAIQRCGPDSSVDELADFIGAARLAKAADA
HMVDLPEVKPTSYLGSKRRKHLNGDLLKRKKIRGLNEDEEAIRLPNPMIGFGIPGELCPVINRSLPEAAI
GPPFFYYENVALAPRGVWNTISRFLYDVQPEFVDSKYFCAAARKRGYVHNLPVVNRYPLVPLPPRTIHDA
LPLTRKWWPSWDTRTKLNCLQTCIASAKLTDRIRMALEDWEGDPPLNVQKYVLNECRKWNLVWVGRNKLA
PLEPDEVEMLLGFPKNHTRGGGISRTDRYKSLGNSFQVDTVAYHLSVLKEIFPGGINMLSLFSGIGGAEV
ALHRLGIPLKNVVSVDISKQNRNIVRDWWEQTNQKGNLIDIADVQTLNADRLEQLMSSFGGFDLVVGGSP
CNNLAGSNRHHRDGLEGKQSSLFYDYFRILDVVKGVMTKGM

>Erodium_cicutarium_DNMT2-g111359.t1
MGKKTAAKSKPTTGDSKADSNSGEGTKANQKRNRPESNQNVTASRKMPKRAAACSDFKDKTVRLSEKSSM
VETKREVIVEDEVAALSLTSGVNDPRPNRRLTDFIFHDADGKAQSVEMLEVDDLFITGVVLSLEESSDKE
KGKGIRCEGFGRIESWDISGYEEGSPVIWISTDFADYDCVKPASGYKKLYDRFFEKARLCIEVYKKLSSK
TGGNHDLSVDELISHVVRSVSGSKNFPHGTSVKDFIVSQGEFIYNQLTGLDETSTNSDLIFRDLPVLAAL
REESRKHTNFAPPKAMTTGGTLKIGEREKVDQSHSPASGAEEDEDMKLARLLQEEENWRSMKPKKGQRSV
ASSKKFYIKINEDEIADDYPLPAYYKTTTQETDEYIIFDNDISDPNELPRSMLHNWSLYNSDSRLISLEL
LPMKTCADIDVTIFGSGVMTADDGSGFCLDSDPNQSSSGSGGAQNIDGIPIYLSAIKEWMIEFGSSMIFI
SIRTDMAWYRLGKPSKQYAPWYEPVLKTARLAIGIITLLKQQSRVSRLSFGDVIKRVSEFDKNHPAHISS

NPAAVERYAVVHGQIMLQQFAEYPDDAIRKSAFVTGLYENMERKHHTKWVVKKKMVLTKEMNMNPRAAAA
PLLSKRKAMPATTTKLVNRIWGQYYSNYLPEDNKEADINEVKEDEDADAEADADEEQEENVEEDTDETDQ
KAMIEQKTEAPRASSKQTKSRRSSSDVKWDGDSVGKTSSGELLYKQAMVHGDMVVVGGSVSVETADTDEV
APIYYVEYMFEKSDGTKMVHGRLMLRGSQTVLDNTANEREVFLTNDCLEFELGDVKQVVTVEIRLMPWGY
QHRKANALKEKIDREKAEERKRLGLPMEYYCKSLYWPERGAFFCLPTDSMGLGSGLCQSCKINEAETEKE
ICKVNSDRSGFTYQGTEYSIHDFVYIAPHHFETDGKDQGTHKSGRNIGLKAYTVCQLLDISAPKVSKKAN
PESMEVKVRRFFRPEDISTDRPYCSDIREVYYSELSWMVTLAAIEGKCDVRRKHDLPPSDGPVIFEHIFF
CERLFDPDSGAIKQLPAQVKLSSSKGKLPDDAAYRKKKGKSKAVENEFDDADRQPDALHKNRLATLDIFA
GCGGLSEGLEKAGVSLTKWAIEYEAPAGEAFTLNHPEALMFVNNCNVILRAIMSACGDEDDCIATPEATE
LAAKLDEKEISSLPKPGQVEFINGGPPCQGFSGMNRFNHSTWSKVQCEMILAFLSFAEYFRPKFFLLENV
RNFVSFNKGQTFRLTIASLLEMGYQVRFGILEAGSYGVSQSRKRAFIWAASPEEILPEWPEPMHVFASAE
LKIALSKNTHYAAARSTANGAPFRAITVRDTIGDLPPVGNGASLTTMEYKNEPVSWFQKKIRGDMMVLND
HISKEMNELNVIRCQRIPKKPGSDWRDLPDEKVRLSSGQLVDLIPWCLPNTAKRHNQWKGLFGRLDWEGN
FPTSITDPQPMGKVGMCFHPDQDRILTVRECARSQGFPDSYKFAGNIQHKHRQIGNAVPPPLAYALGTKL
KEAVEKKRKELEVEVRETHYIRLLPPSPSLPATAMALAMVFVGHRQSELPPPCSSPALQRTGSTTFSPAF
RLRSDRGTRFPTFAGEDRCRSGFFGAEVGVGSWLLLFLDVLVGLTPALLVISIRGTLFFVVADKDCGGLG
LVRRC

>Erodium_cicutarium_DNMT2-g17877.t1
MGSAKVLSEPASDEVETQSSGMGKKTAAKSKPTTGASKADSNSGEGTKANQKRNRPESNQNVTASRKMPK
RAAACSDFKDKTVRLSEKSSMVETKREVIVEDEVAALSLTSGVNDPRPNRRLTDFIFHDADGKAQSVEML
EVDDLFITGVVLSLEESSDKEKGKGIRCEGFGRIESWDISGYEEGSPVIWISTDFADYDCVKPASGYKKL
YDRFFEKARLCIEVYKKLSSKTGGNHDLSVDELISHVVRSVSGSKNFPHGTSVKDFIVSQGEFIYNQLTG
LDETSTNSDLIFRDLPVLAALRDESRKHTNFAPPKAMTTGGTLKIGEREKVDQSHSPASGAEEDEDMKLA
RLLQEEENWRSMKPKKGQRSVASSKKFYIKINEDEIADDYPLPAYYKTTTQETDEYIIFDNDISDPNELP
RSMLHNWSLYNSDSRLISLELLPMKTCADIDVTIFGSGVMTADDGSGFCLDSDPNQSSSGSGGAQNIDGI
PIYLSAIKEWMIEFGSSMIFISIRTDMAWYRLGKPSKQYAPWYEPVLKTARLAIGIITLLKQQSRVSRLS
FGDVIKRVSEFDKNHPAHISSNPAAVERYAVVHGQIMLQQFAEYPDDAIRKSAFVTGLYENMERKHHTKW
VVKKKMVLTKEMNMNPRAAAAPLLSKRKAMPATTTKLVNRIWGQYYSNYLPEDTKEADINEVKEDEDAEA
DAEADADDEQEENVEEDTDETDQKATIEQKIEAPRASSKQTKSRRSSSDVKWGGDSVGKTSSGELLYKQA
MVLGDMVVVGGSVSVETTDTDEVAPIYYVEYMFEKSDATKMVHGRLMLRGSQTVLDNTANEREVFLTNDC
LEFELGDVKQVVAVEIRLMPWGYQHRKANALKEKIDREKAEERKRLGLPMEYYCKSLYWPERGAFFCLPT
DSMGLGSGLCQSCKINEAETEKEICKVNSDRSGFTYQGTEYSIHDFVYIAPHHFETDGKDQGTHKSGRNI
GLKAYTVCQLLDINAPKVSKKANPESIEVKVRRFFRPEDISTDRPYCSDIREVYYSELSWMVTLAAIEGK
CDVRRKHDLPPSDGPVIFDHIFFCERLFDPDSGAIKQLPAQVKLSSSKGKLADDAAYRKKKGKSKAGENE
FDDADRQPDALHKNRLATLDIFAGCGGLSEGLEKAGVSLTKWAIEYEAPAGEAFTLNHPEALMFVNNCNV
ILSAIMSACGDEDDCIATPEATELAAKLDEKEIGSLPKPGQVEFINGGPPCQGFSGMNRFNHSTWSKVQC
EMILAFLSFAEYFRPKFFLLENVRNFVSFNKGQTFRLTIASLLEMGYQVRFGILEAGTYGVSQSRKRAFI
WAASPEEILPEWPEPMHVFASAELKIALSKNTHYAAARSTANGAPFRAITVRDTIGDLPPVGNGASLTTM
EYKNEPVSWFQKKIRGDMMVLNDHISKEMNELNVIRCQRIPKQPGSDWRDLPDEKVRLSSGQLVDLIPWC
LPNTAKRHNQWKGLFGRLDWEGNFPTSITDPQPMGKVGMCFHPDQDRILTVRECARSQGFPDSYKFAGNI
QHKHRQIGNAVPPPLAYALGTKLKEAVEKKRKELEGSVLRLSSGQETVLDRSGVKGRALRSPKPLRPVRV
DRLPVTADGHKEVVGGGSDNDVDGRGRGLSVDRDLGRGIEKRAVADWVMLGDFKEELVLHDISIQGYHLA
QWERESHEDGGPIGPCFCVNTNTSPEAGAEGTKLLSIHELAISSFYLGVPTRNEVLVKSVLQSIPNYLLY
ECDAPPVGSGGANQQGPGKILVIIPEDVCSGSTCGLQPELCVDEYTIYVGIDQGQTSHWTGDLHVYPLST
MVNRDVRESFAVPIILSVPMESSRYPVRHLSQIWNCRLGVSKIGPQPSPPGPPGPSGLVPMGYGDGPGFR
IIKPGVSGPGNGSVT

>Erodium_cicutarium_DNMT2-g55333.t1
MHGGGSLVLGRPKYNSTLSYLRLGVDGNLRVFMLYPKVDYGAWAATSTLFSAQSNWGDECQLPERCGNFG
LCENNQCVACPSPTCLLGWAKDCKPKLLSGCEPSKFKYYKIGGVEHYSSRLSQKLTLQATWLMSNEFSED
SVEVYVHWEGESVGSTSSGNTLHKRAIVCGHTVTAGGFVSVDSGDTPIYFIEYMFENSDLRKMFHGRLML
RGFETVLGNAAKEREVFFTNECREFELCDVKETISIEIQIMPWGYQYRKCNADKARVAEERAKERKWQRL
AREYYCKSLYWPERGAFFSLQPDTMGLGNGLCRSCVMKEAQREKEIFKVYYSEQVLSVPVTVIEGKCEVR

MKHDLPSLYGPFEHIFFCEHLYDPHNGNVEELPSHITLGSSKETNSESTDTSHKNPFSTLDIFAGCGGLS
EGLQRAGASLTKWAIEYEEPAGEAFSLNHPEAKVFVNNCNVILRAVMSACGDADDCFSTSEASELAAKLD
EEVIRNLPRPGQVEFINGGPPCQGFSGLNRFSQSRWSKVQCEMILAYLSFADYFRPKFFLLENVRKFVSF
NKGRTFRLTLALLLEMGYQGVLGWELPNFSNGSSTGEYIWNVLPPGSGSDTYCSRMFSIPKCNTKISSGH
FCAINKEEGFPDSYKFSGNIQNKHRQVGNAVPPPLAYALGRMLKEAVEKKHSHYST

>Erodium_cicutarium_DNMT2-g85043.t1
MERDNQAEPKFAISKPRPKRARSTSKMPKQALTSEVEYEFEAIRLTHGLDDHRPNRRLTDFIFHDVDKEP
QPIKTLELKSLFISGSILPLEESSDKKKERGVRCDCFGRVESWAISGYDEGTPIVWVSTAVADYECVHPG
KEYEEMYYVFFEKARACVEIYKELLSCGRNPVMTVDELLARVVRSMSDSKSFGCEAEIKDFIVSKGEFIY
NELIGLDGTSETDDLVFRDLLVLAALRDEAKRHSHFVPPEVTLLGVLSPRQGVEEREQINKYASHASGAE
EHDDEALARLLQEQEYRRSSRQKKGKCRDIVLIKLDIIVYVSSGALMYYRGNFTQLSLRIAKVSEGMCNF
SDPQPNQKRPRPTSTERTCNSPKRPKKRQVRVHHEVKAVTMTSEQDEPRPNRRLTNFTFHDAEGNTQSLE
MLEAKVLFTSGLVLPLEESSQKPKETGVRCKNFGHIQSWAISGYEEGLPVIWVSTEACDYECVSPAKGYE
KKYNVFFEKARACIEVYMKLSSSCGGNPNLSIDELLVRVGQSMSYGKNFTCEDEVKRFVCSQGEFIYKEL
IRLEEAFRALPVLVALRDDTKNHEHFVPSILKLGDGEQVNELSSSVLGADEDGELKLARLLLEEENWRAT
IQKRVEHEVEAVNLTYGQEDSPQPNRRLTSFIFHDADGKPQQLEKLQIDVLFASGLILPLEESSKNEKQN
CVRCEGFGRIESWAISGYEEGSPVIWISTGVADYECVSPAKGYETIHNILLDKAHACIEVYMKLSMACGG
SPDLSLNDLLARVVQSMSSAKTCTNEDEVRYFVVSQGEFIYNELIGLDDTSNDDQMFRGLPVLVSLANEA
KKHRHSMPRRARRHKVGSSGCGDDEEEIKTCGTGELPRRMVIHNWSLCDSYSRLVSLELLPMKACCDDNH
TIFGLGVMAVDDGSGLSLDDDSNQLCSDPVGVQNVDGIPIYLSAIREWKLEFSSSMVFVSIRTDTCWYRL
GKPSKQYAPLHEQVLKTTKLGLSIILLLKEQSRVARLSFTDVINRLSKFRKDHPTYISHDPAEVERHVVI
HGQILLQQFADYPEDVINRCAFITTLRDRIKWLEKKRMYLNNKVSSDPSTETEASSSPSLSPSSSLALET
KAMPATKTRMVNRVWREYYSNEFSEDSDEIDIHWDGESVGSTSSGNALYKRAIVCGHTVTVGGFVSVNSG
DNPIYFIEYMFENSYLRKMFHGRLMLRGFETVLGNAANEREVFFTNECREFELCDVKETISVEIQTMPWG
YQYRKGNADKAQVAEERAKERKWQRLAREYYCKSLYWPERGAFFSLQPDTMGLGNGLCRSCVMKEAQREN
EIFKVNPSLSSFMFKGTEYCVNDFVYVVPRHFAIDEKDHAKFKGSRNVGLKAYVICHLLEIEVMEGAEKA
CPKSTHVKVRRFFRPEDISASKAYCSDIREVYYSEQVLSVPVMVIEGKCEVRMKHDLPSLYGPFEHIFFC
EHLYNPHNGNVEELPSHITLRSSKETNSESTDTSQKNPLATLDIFAGCGGLSEGLQRAGASITKWAIEYE
EPAGEAFSLNHPEAKVFVNNCNVILRAVMSACGDADDCFSTSEASELAAKLDEEVIRNLPRPGQVEFISG
GPPCQGFSGLNRFSQSRWSKVQCEMILAYLSFADYFRPKFFLLENVRKFVSFNKGQTFRLTLALLLEMGY
QVRFGILDAGAYGIPQSRKRVFIWAASPEETLPEWPEPMHVFAGPELKVTLSESTQYAAVRSTAHGAPFR
SITVRDAIGDLPVVGNGASAVTMQYGNEPVSWFQKQIRGDGKFLNDHISKPMNELNLIRCQRIPKRPGAD
WRDLPTEEVELSTGVRANLVPSWVLNTAHRHSNWKGVLGRLCWDGNFPTSVTDPQPEGTVGMCFHPDQDR
ILTVRECSRSQVSPSPSQFLVKGGFSDSYKFSGNIQNKHRQVGNAVPPPLAYALGRMLKEAVEKKHSTTQ
PK

>Erodium_cicutarium_CMT1-g102681.t1
SASRSLSNFHFPYLLPLPKSPIFTCSFLFLSPIAAALRPISTMPRKRSSESNKASSKKRKLPKEEEEDPE
LIEELESQFDEETLRIIEQGQSSSAAPKREISRDPDEDVEEKPKDLAFSTSPRKLRADEDDVTPDSRFLG
EPVEDEEARRRWPHRYQYKMKKRGADSEGLKEKDDSEEPMQARRHFLKAEVDGRVIFDLCDNAYVEAEEG
KDSYICKIIEMFEGVDRKPYFTAQWYYRAKDTVMKDHGNLIDAKRVFFSEIKDDNPLDCLIQKLQIVKVE
LETKKVAIPACDYYYDTLYLLPYSTLIKAPSDGRTSSEMSTVSSDVNMSPKNDRKSDVVEDCQIEDHKPE
VTLLDLYSGCGAMSTGLCLGASLSGLNLVTKWAVDMNEYACQSLKLNHPETEVRLFTSFVHLAVVVGLIH
FANRNESQPFIDHFAKEEEKEDSEDDDDTENAVDDPEVFEVESLLGICFGDPNGIKERKLHFKVRWKGYG
SDEDTWEPLEGMGDCRERVKEFVTKGFTSKILPLPGGADVICGGPPCQGMSGFNRFRNKDAPLADIRNKQ
LLVFMETVGYLKPKFVLMENVVDLIKFAKGYLGRYALARLVQMNYQARIGMMAAGAYGLPQFRMRVFLWG
ALPTQKLPQYPLPTHDVVVRGGTPVEFEKNAVAYEEGDQVQLENRLYLKDAISDLPPVENSENRDEMFYD
RDPETEFQKLIRLSKEEMMGEILQSQSPIKLYDHRPLNLNTDDYQRSCQIPKRKGANFRHLPGVLVGSDN
KVEWDPNVKRVLLESGKPLVPDYAMTFVSGTSSKPFARLWWDETVPTVVTRAEPHNQKILHPEQDRVLTI
RENARLQGFPDYYKLCGPVKQRYMQVGNAVAVPVARALGYALGLAFQGSASDEPLLKLPQGFPNLVERAS
SSSSEEVA

>Erodium_cicutarium_CMT1-g127965.t1

MESPTRSGSASGSRSKCWVLPVSEPQGVPDQPVRFDPNSSGDVDSQSILKSLLTAHPISDIRSGDGQGSM
RSSPAENGDKEAYSHGGTSHAESLRKSPRLDPAENGNLKKLHQKLSPRSKMSDEDRNGNGVSSNLFDTWE
EGLPQRLAFPDGGESGSLEGVLAQSQGSNGLHLMQLSDIKELHLNVESSADKCSGRSRRLSSAQSGSKSN
RSSATLQGMSLRNARSSDKDCFGSVSPSKYNLKNISQAEFKTKISSLSRHTEADGESVALKYNNQKNSKQ
LRRSPRLILESSNSGTNVSFLRSFDIVLLDKKKIRRSPRLCSPLSGVKTIELKSSDEQPPKKTKTSVASP
GSRALDEKQLKRAPRSESDEPPSKKIKTSSGKFDPCMMEGKLFRRSPRNATSPSGNKFDEMKCDFKSAEM
RCRTNNTTSASSENKQKNHKNTSFFVGDPIPSEGTPTTDKKFLRQPSRIASCPVGEFEKTKCSSVKLQES
TEKRSLTNTKTTISSENEQNYKVVCSFVGDPIPTSEAQERWHWRYELKSRRSKHRRMTEENDDEDMVISN
VECHYNQAEVNNCTYSLGDCAYIKGDGGEKHIGRIIEFFKTTDGENYFRVQWFYKVEDTVIKEANFHDKK
RIFYSTVMNDNLIDCILSKVSTVQILPKTGLKSNFVTSADFYFDMEYCVEYSTFRNMLPDDSLKSCDKSP
TNHIEMIPTTTLLNDKPSCGTSEGELALLDLYAGCGGMSTGLSLGVKNSGVNLVTRWALDSDKAACKSLK
LNHPETHVRNEAAEDFLELLKAWEKLYKRYDTYDVNSIRHSRSKDSQAVDDAVHSLSEGEDSADEFEVSS
VVDICYGDPDKTGKHGLKLKVNWRGYDSSEDTWEPIEGLSNCQERIQDFVRTGLKSKILPLPGDVDVICG
GPPCQGISGYNRFRNTDSPLDDERNRQIIVFMDIVKFLKPKFVLMENVVDILKFDNASLGRYALSRLVSM
RYQARLGIMAAGCYGLPQFRLRFFLWGAHPSEKLPQFPLPTHDVIVRYWPPAEFERNTVAYDEDQPRDHL
EKALVLQDAISDLPEVKNDEIREEMPYEKLPQTEFQRYIRSITVFAPPFCDCLEMTGSGFPGKPEEVLLY
DHRPHLLNEDNYARVCQIPKRKGANFRDLPGVVVGADNVVRRDTTKEQILLPSGKPLVPDFAFTFEQGRS
KRPYGRLWWDETVPTVVTNPNYRSQAALHPEQDRILTMREYARLQGFPDYYRFYGNISERHRQIGNAVAV
PVARALGYALGMAFQKLGDNKPLMTLPPKFAYSNNIQCTENDTKKPSGDEPLNTLSNKIHCTETNTQKPS
GDELLNTLSLTTSDTNNTQSTECQNTEYKPPLMRELPEFSLSNNSQFGNDGIQKTKEEASFGPSEFSHPS
SLNLHCDHNCYVSSECFILISWELVQREWALLTSATPDNPCDPSPKNRLLRMTTSSHWPWAHTSILHQPW
LMGNNPGIQTVSPVLAEAKVVNLKLLDKHGCHSTRTGYRLFREIITKMRTRVIPLWVDLWQILAQLRLCI
FLWRVLTGCLQIVVRLHQRLVPVSSICSICDRHQETTNHLLIYYGFATTCWIKFGPMPAVFPNISFSAWQ
GLMIVGATPDRRGRSTLSGYRPIGMGFLRTLFYRPGCWSNPDPLVPDDD

>Erodium_cicutarium_CMT1-g139908.t1
MWRGTPLFDLPGLISSQICPYASSLCLVWPGSLSRLVRLSEVRIRRFVGVGLWRVVRSLCLWMRTSYFDF
RSSEFWWVSPTRSVSAICGVDRRLVSVRPTLVKGSMALISQGKSQSLAETFLYWFDPCRIGCCIGLIRVG
SVVVLFDFVELKKNTSQLFSSSSMESPTRSGSASGSRSKCWVSEPQGVSFDPKSSGEDEDVDSQSILKSS
PSLLTAHPISDIRSGDGQGSSPAENGAKEAYSEASHGGASHAESLRKSPRLDPAENGNGSPNLKKLSPRS
GEDRNGNGVSSNLFDSWEEGPPQRLAFTDGGQSVSLEQCQGSNGLHLMLLSDKKELHLNVESSAEKCFGR
SRRLSSAQSGSKSNRSSATLQGMSLRNARSSGGDCFGAVSPSTYNLKNRSPAEFKTKISSLSQHTEADGE
SVALKYNNQKNSEHLRRSPRLILESSNSGTNVSFLRNFDIVLLDKKKIRRSPRLCSPLSGAKTIELKSSD
EQPPKKTKTSVASPGPRALDEKQLKRAPRSASDEPPSKKIKTSSGKFDPSMMDGKLLRRFPRNATSPSGN
KFDEMKCDFESTEMRCRTNNTTSASSENKQKNHKNTSFFVGDPIPSEGTPTTDKKFLRQSSRIASCPEGE
FEKTKCSSVKLQESTEKRSLTNTKTSISSENEQNYKVVCFFVGDPIPTSEAQERWHWRYELKSRRSKHQR
MTEENDDEDMVISNVECHYNQAEVNNCTYSLGDCAYIKGDGGEKHIGRIIEFFKTTDGENYFRVQWFYKV
EDTVIKEANFHDKKRIFYSTVMNDNLIDCILSKVSIVQILPKTGLKSNFVTSADFYFDMEYCVEYSTFRN
MLPDDSLKSCDKSPTNHIEMIPATTLLNDKPSCGTSEGELALLDLYAGCGGMSTGLSLGVKNSGVDLVTR
WALDSDKAACESLKLNHPETHVRNEAAEDFLELLKAWEKLYKRYGTYDVNSIRHSRSKDSQAVDDAVHSL
SEGEDSADEFEVSSVVDICYGDPDKTGKHGLKLKVNWRGYDSSEDTWEPIEGLSNCQERIQDFVRTGLKS
KILPLPGDVDVICGGPPCQGISGYNRFRNTDSPLDDERNRQIIVFMDIVKFLKPKFVLMENVVDILKFDN
ASLGRYALSRLVSMRYQARLGIMAAGCYGLPQFRLRFFLWGAHPSEKLPQFPLPTHDVIVRYWPPAEFER
NTVAYDEDQPRDHLEKALVLQDAISDLPEVKNDEIREEMPYEKLPQTEFQRYIRSITVFAPPFCDCLEMT
GSGFPGKPEEVLLYDHRPHLLNEDNYARVCQIPKRKGANFRDLPGVVVGADNVVRRDTTKEQILLPSGKP
LVPDFAFTFEQGRSKRPYGRLWWDETVPTVVTSPNYRSQAALHPEQDRILTMREYARLQGFPDYYRFYGN
ISERHRQIGNAVAVPVARALGYALGMAFQKLGDNKPLMTLPPKFAYSNNIQCTENDTKKPSGDEPLNTLS
NNIQCTETSTQKPSGDELLNTLLLTTSDTNNTQSTEIECQNTEYKSPLMRELPEFSLSNNSQLGNDGIQK
TKEEASFGTSEFSKPGNLNLHCDHNCYVSSGCFILISWALVKRVRIFDPSPHNGRTTSSRGHGTDTSILH
QSWLMGSSPMIQTVSPVLAEAKVVNLKLPGTRTWDTGLCPIRSHKLRTNGIGRKTNMAAIPLERARGPRS
SLLCGWITGSAPNLHISIEGPHGLSRDCCPLTPTLLRTSVDGALFPDTGQSGQGFLGRSSTGQPAGPIMT
LWSPTMIEAHVIDRDGPTSRKLVTTPSNPSSPGGLGTCLSEAGAIQNLLPNQEPFLFGTRNLQASMATTT

APAVHQSRKGTTTFSLPDARMTASPPAQKKRSMDAPTPAKETAAPEHIETSSCQDQWNSDNMVPPYPDVL
PGCPLSPQAPPIFDYGHFHIEAGNDHRMLLSHT

>Erodium_cicutarium_CMT1-g1474.t1
MPRKRSSDSHKACSKKRKLPKEEEDPELIEELESQFDEEALRIIEQGKSTSAAPKREISRDPDEDVEEKP
KDLAFSGTAVRSPRKLRVDDEDDTPDSRFLGETVEDEEARRRWPHRYQYKQKKKGAGSEELKEKDDSEEL
MQARKHFIKAEVDGRVIFDLYDNAYVKAEEGEDSYICKIIEMFEGLDKKPYFTAQWYYRAKDTVMKDHGN
LIDPKRVFFSEIKDDNPLDCLVQKLQIVKVELDMKKVAIPACDYYFDTLYLLPYSTLIKAPSDGRTSSEV
STVSSDVDMGPRNDVKSDVVEDCQVEDHKPEVTLLDLYSGCGAMSTGLCLGASLSGLNLVTKWAVDMNEY
ACQSLKLNHPETEVRNESAEDFLSLLKEWESLCMSFSLVSRNESQPFIDHFAKEEEDEYSEDNDDTENAV
DDPEVFEVESLLGICFGDPNGIKERKLHFKVRWKGYGPDEDTWEPLEGMGDCRERVKEFVTKGFSSKILP
LPGGADVICGGPPCQGVSGFNRFRNKDAPLADTKNKQLLVFMETVEYLKPKFVLMENVVDLIKFAKGYLG
RYALARLVQMNYQARIGMMAAGAYGLPQFRMRVFLWGALPTQKLPQYPLPTHDVVVRGGTPVEFEKNAVA
YEEGDQVQLENRLYLKDAISDLPPVENSENRDEMLYDRDPETEFQKLIRLRKEEMMGAILQSQLPIKLYD
HRPLNLNTDDYQRSCQIPKRKGANFRHLPGVLVRSDNKVEWDPNVERVLLESGKPLVPDYAMTFVNGTSS
KPFARLWWDETVPTVVTRAEPHNQDSLPISKDISQTATMEWCKREAQALKTRSGGCLVLRGVDRYHDPFR
DKPACTGKPGRIDRTWAIGFPFPIRPHLLRVRGKPRVSSSQTVPSENNGRVVGVWVIEHDHLSLLGEICC
VKLYVGVPETKSGEGGDRSRHGEPSLAVIFSVQTRTDGWAYLKFYIRSKIESLPFERMQDSKDFQIIINS
AGLYMQVGNAVAVPVARALGYALGLAFQGSASDEPLLKLPQGFPNLGERASSSSSEEVA

>Erodium_cicutarium_CMT1-g161730.t1
MPGKRSSGSAAACSKKKKAASSKKRKLAEEPEISEELEGQFDQEAVQAIEEGNVSSGAANAGCSSGARKD
LGQPDEELELKPKARVVSAGSPRKSRAHTDEDEPDSRFLGEPVPEAEARQQWPHRYLDKKQKKLDGLKYL
NMDDSEELIQAQRHYKQAEVDGHIIFNLYDDAYVKAEDGEDNYICKIIEMFEATDGALYFTAQWFYRAKD
TVIKDHGSLIDSKRVFFSEIKNDNPLDCLLEKLNIVKINLEAKQNSIPPCDFYYDTLYLLPYSTFIKAPP
ADNKTASEPSSSISSEVVVNETKEKCDSMEGCLVGQSHKPELTLLDLYSGCGGMSTGLCIGANLSGVNLI
TKWAVDLNKYACESLKFNHPETEVRNEAAEDFLSLLKEWKKLCVSFSLVSRDDEQPYLDPYAMNDEIEDS
GDDDDDAEDESDPEVFEVEKILQACFGDPNEKGTRQMHFKVRWKGYGPDEDTWEPLEGLGGCRDRLKEFV
TEGFNSKTLPLPGDVDVVCGGPPCQGVSGFNRFRNKENPLADEKNKQLIVYMEIVEYLKPRFVLMENVVD
LIKFAGGYLGRYALGRLVHMNYQARMGMMAAGSYGLPQFRMRVFLWGARPTEKLPQYPLPTHDVVVRGVI
PLEFEQIVVANDEKDKVDLEKKLFLKDAISDLPAVENFEQRDEMPYGKDPETEFQQTIRLSKNELLGIST
QQDSSTKLLYDHRPLQLNTDDYQRSCRIPKEKGANFRHLPGVLVGDDNKVKWDPDVPRVLLDSGKPLVPD
YAMSFVKGSSSKPFARLWWDEIVPTVVTRAEPHNQAILHPLQDRVLTVRENARIQGFPDYYKLCGTVKER
YMQVGNAVAVPVARALGYSLGLAFQGSISDEPLMKLPPKFPNIIEQVCESSEGVV

>Erodium_cicutarium_CMT1-g182635.t1
MPRKRSSDSHKACSKKRKLPKEEEDPELIEELESQFDEEALRIIEQGKSSSAAPKSEISTDPDEDVEEKP
KDLAVSGTSVRSPRKLRADDEDDTPDSKFLGEPVEDEEARRRWPHRYQYKQKKKGADSVELKEKDDSEEL
MQARKHFIKAEVDGRVIFDLYDNGYVKAEEGEDSYICKIIEMFEGLDKKPYFTAQWYYRAKDTVMKDHGN
LIDPKRVFFSEIKNDNPLDCLIQKLQIVKVELQTNKVAIPACDYYFDTLYLLPYSTLIKAPSDGRTSSEM
STVSSDVDMCQKNDRKSDVVEDCQVEDHKPEVTLLDLYSGCGAMSTGLCLGASLSGLNLVTKWAVDMNEY
ACESLKLNHPETEVRNESAEDFLSLLKEWESLCMSFSLISRNESQPFIDHFAKEEEKEDSEDDDDTENAL
DDPEVFEVESLLGICFGDPNGIKERKLHFKVRWKGYGPDEDTWEPLEGMGDCRERVKEFVTKGFSSKILP
LPGGADVICGGPPCQGVSGFNRFRNKDAPLADTKNKQLLVFMETVEYLKPKFVLMENVVDLIKFAKGYLG
RYALARLVQMNYQARIGMMAAGAYGLPQFRMRVFLWGALPTQKLPQYPLPTHDVVVRGGTPVEFEKNAVA
YEEGDQVQLENRLYLKDAISDLPPVENSENRDEMLYDRDPETEFQKLIRLRKEEMMGAILQPQPPIRLYD
HRPLNLNTDDYQRSCQIPKRKGANFRHLPGVLVRSDNKVEWDPNVERVLLESGKPLVPDYAMTFVNGTSS
KPFARLWWDETVPTVVTRAEPHNQASLTATYTRRVLKCPLRKFYIRSKIESLPFERMQDSKDFQIIINSA
GLYMQVGNAVAVPVARALGYALGLAFQGSASDEPLLKLPQGFPNLVEQASSSSSEEVYVLAADTLAEQPD
RRRPRNRAGRGHCGGEGKGDAAGVKGVADHERELSPDKLGEGLDTPSSPCPYGLYLVVSTLVLAVF

>Erodium_cicutarium_CMT1-g56658.t1
MKKRGADSEGLKEKDDSPMQARRNFLKAEVDGCVTFDLCDNAYVYAEEGKDSYICKIIEMFEGVDRKPYF
TAQWYYRAKDTVMKDHGNLIDPKRVFFSEIKDDNPLDCLIRKLQIVKVELETKKVAIPDCDYYYDTLYLL

PYSTLIKAPSDGRTSSEMSTVSSDVDMSPKNDRKSDVVEDCRVEDHKPEVTLLDLYSGCGAMSTGLCLGA
SLSGLNLVTKWAVDMNEYACQSLKLNHPETEVRNESAEDFLSLLKEWESLCMSFSLVSRNESQPFIDHFA
EEEEKEDSEDDDDTENAVDDPEVFEVESLLGICFGDPNGIKERKLHFKVRWKGYGSDEDTWEPLEGMGDC
RERVKEFVTKGFTSKILPLPGGADVICGGPPCQGMSGFNRFRNKNAPLADIRNKQLLVFMETVEYLKPKF
VLMENVVDLIKFAKGYLGRYALARLVQMNYQARIGMMAAGAYGLPQFRMRVFLWGALPTQKLPQYPLPTH
DVVVRGGTPVEFEKNAVAYEEGDQVQLENRLYLKDAISDLPPVENSENRDEMLYDRDPETEFQKLIRLSK
EEMMGEILQSQSPIELHDHRPLNLNTDDYQRSCQIPKRKGANFRDLPGVLVGSDNKVEWDPNVKRVLLAS
GKPLVPDYAMTFVRGTSSKPFARLWWDETVPTVVTRAEPHNQSLSLQSICSGKNGEILSRKFYIQSKIES
LPFERMQDSKDFQTIINFAGLYMQVGNAVAVPVARALGYALGLAFQGSASDEPLLKLPQGFPNLGEQPSS
SSSEEIA

>Erodium_cicutarium_CMT3-g105204.t1
RTKINMDGSLLAGFGGGGGAVIRDEAGSVQHARVFNFPDVTEPEIVEVLVVREVLLWLRQLGWELGDLET
DCLSVVYAINSSEVIYSPFGGLVEDCKRVLRTLLGCRVSHVRREANQVAHTLAKHAVVWASLELDCNNLP
EPIPGMEKCPDLEGTQRPCPVRDGLGVEQAILSPFGCLFLSPIAALRPIFTMPRKRSSGSHKACSKKRKL
PKEEEPELIEELENQFDEEAVRIIEQGNSSSAAPKSEISRDPDQDVEEKPNDLAFSGTAVRSPRKLRADD
EDDTPDSRFLGEPVEDEEARRRWPHRYQYKKKKQGADSEELKEKDDSEELMRARKHFVKAEVDGCVFFDL
YDNAYVKAEEGKDSYICKIIEMFEGLDKKPYFTAQWYYRAKDTVMKDHGNLIDPKRVFFSEIKDDNPLDC
LLQKLQIVKVELQTKKVAIPACNYYFDTLYLLPYSTLIKAPSDGRTSSEISTVSSDVDMGPSNDGKSDVE
LVEDCQVEDHKPEVTLLDLYSGCGAMSTGLCLGASLSGLNLVTKWAVDMNEYACQSLKLNHPETEVRNEE
AEDFLSLLKEWESLCMSFSLVNRNESQPFIDHFAKEEEKEDSEDDDTENAVDDPEVFEVESLLGICFGDP
NGIKERKLHFKVRWKGYGSDEDTWEPLEGMGDCRERVKEFVTKGFSSKILPLPVSGVDVICGGPPCQGVS
GFNRFRNIKAPLADIRNKQLLVFMETVEYLKPKFVLMENVVDLIKFSEGYLGRYALARLVQMNYQARIGM
MAAGAYGLPQFRMRVFLWGALPTQKLPQYPLPTHDVVVRGGTPLEFEKNAVAYEEGVQVQLENRLYLKDA
ISDLPPVENSENRDEMLYDRDPQTEFQKLIRLRKEEMMGVILQSQSPIKLYDHRPLNLNTDDYQRSCQIP
KRKGANFRNLPGVLVRSDNKVEWDPNVERVLLESGKPLVPDYAMTFVKGRSSKPFARLWWDETVPTVVTR
AEPHNQRILHPEQDRVLTVRENARLQGFPDYYKLCGPVKQRYMQVGNAVAVPVARALGYALGLAFQGSSS
DEPLLKLPQGFPNLGERESSSSSEEVA

>Erodium_cicutarium_CMT3-g41200.t1
MISWRVSRADVKPESNIVHTPPIIPVPAAFSKSSLLFSSLNRTNLVSLYSPSPSSFSLPSPPSAAAAMPG
KRSSGSAAACSKKKKAASSKKRKLAEEPEISEELEGQFDEEAVQAIEEGKVSSGAANAVGSSRARKDLSQ
PDEELELKPKARVVSAGSPRKSRAHTGEDEPDSRFLGEPVPEAEARQQWPHRYLDKKQKKLDGPKYLNMD
DSEELIQAQRHYKQAEVDGLIIFNLYDDAYVKAGDGEDNYICKIIEMFEATDGELYFTAQWFYRAKDTVI
KDHGTLIDSKRVFFSEIKNDNPLDCLLEKLNIVKIDLEAKQNSIPPCDFYYDTLYLLPYSTFVKAPPDNK
TASEPSSSISSEIVVNETKENCDSMEGCHVGQSHKPELTLLDLYSGCGGMSTGLCIGANLSGVNLITKWA
VDLNKYACESLKFNHPETEVRNEAAEDFLSLLKEWKKLCVSFSLVSRDDEQPYLDPYAMNDEIEDSGDDD
DDAEDESDPEVFEVEKILQACFGDPNEKGTRQMHFKVRWKGYGPDEDTWEPLEGLGGCRDRLKEFVTEGF
NSKTLPLPGDVDVVCGGPPCQGVSGFNRFRNKENPLADEKNKQLLVYMEIVEYLKPRFVLMENVVDLIKF
AGGYLGRYALGRLVHMNYQARMGMMAAGSYGLPQFRMRVFLWGARPTEKLPQYPLPTHDVVVRGVIPLEF
EQIVVANDEKDKVDLEKKLFLKDAISDLPAVENFEQRDEMPYGIDPETEFQQTIRLSKNELLGISTQQDS
STKLLYDHRPLQLNTDDYQRSCRIPKKKGANFRDLPGVLVGHDNKVKWDPDVPRVLLDSGKPLVPDYAMS
FVKGSSSKPFARLWWDEIVPTVVTRAEPHNQAILHPLQDRVLTVRENARIQGFPDYYKLCGTVKERYMQV
GNAVAVPVARALGYSLGLAFQGSISDEPLMKLPRASKASAYSHPSSMGGSRASGHVRSEQQWVLVTAATL
VCGFVCYLVYDAVMATAAELLQRLLVISPLLLVITVHWLSAGTRLSLPMPGSEPGAIHTAGGSPWGVAFV
LLLLLFLISYQPSFSDLVGL

>Geranium_maculatum_DNMT-g2500.t1
MGSAKVVGKSASGGVDNQSSGMGKKKATNSKSTASDDKKAKPNQKRSRLESNQNIPGSRKMPKRAAACSD
FKDKTVRLSEKSSMVETKRDIVVEDEVAAISLTSGINDPRPNRRLTDFIFHDADGKAQSVEMLEVDDLFI
TGGILSLEENSDKEKGKGVRCEGFGRIESWDISGYEEGSPIIWISTEFADYDCVKPATSYKKLYDLFFEK
ACLCIEIYKKLSKTSGGNPDLSVDELISQVVRPMSGSKNFPHGTSVKDFIVSQGEFVYNQLTGLDETSGK
NDLIFRDLPVLAALKDESKKHTGFAPPKAMSSGGSLKIGEREKANHSNSPASGVVDDEDMKLARLLQEEE
NWRSMKQKKGQRSAASSNKFYIKINEDEIADDYPLPAYYKTTTEETDEYIIFDNDICDPNELPHSMLHNW

SLYNSDSRLISLELLPMKPCADIDVTIFGSGFMTADDGSGFCLDSDPNQSSSGSCEVQNNDGIPIYLSAI
KEWMIEFGASMIFISIRTDMAWYRLGKPSKQYAPWYEPVLKTARLAIGIITLLKEQSRVSRLSFADVVKR
VSEFDKNHPAYISSTPAVVERYAVVHGQIMLQQFAEYPDDAIRKSSFVTSLHDNMERKHHTKWLVKKKMV
LKKEGNMNPRAAMAPLVSKRKAIPATTTKLINRIWGQYYSNYLPEDSKDADINEVKEDEDVDADGDGEQE
ENVEEDIEETDQKTIADKIEAPRASLKLSKARRSSNDIKWDGDSVGKTSCGEVLYKQAMVHGDLVAVGGC
VSVETTDTEDLPLIYFVEYMFEKSDATKMVHGRLLLRGSHTVLGNTANEREVFLTNDCLEFELGDVKQNE
VVEIRLTPWGYQHRKANALKEKIDRERAEERKSKGLPMEYYCKSLYWPDRGAFFCLPTDTMGLGNGLCQS
CKINKSEAEKEICTVNSARSAFTYKGTEYFIHDFAYIAPHYVATDEKDQGTHKSGRNIGLKAYTVCQILD
IDAPKAFKKANPESIEVKVRIFFRPEDISSEKSYCSDIREVYYSETTLMVHLAAIEGKCDVRRKHDLPPS
DGPVIFEHIFFCERLFDPDSGAIKQMPAQVKLRSSKGKLSDDAAYRKKKGKGKEGESMGDDTDKKTDAPH
GNRLATLDIFAGCGGLSEGLEKAGVSLTKWAIEYEEPAGEAFTLNHPEALMFVNNCNVILRAIMSACGDE
DDCISTTEAIELAAKLNEKEISNLPHPGEVEFINGGPPCQGFSGMNRFNHSTWSKVQCEMILAFLSFAEY
FRPKFFLLENVRNFVSFNKGQTFRLTIASLLEMGYQVRFGILEAGAFGVSQSRKRAFIWAASPEEILPEW
PEPMHVFASAELKIALSKNTHYAAARSTASGAPFRAITVRDTIGDLPAVENGASKATMEYKNEPVSWFQK
KIRGDMLVLNDHISKEMNELNLIRCQRIPKVPGSDWRDLPDEKVKLSTGQLVDLIPWCLPNTAKRHNQWK
GLFGRLDWEGNFPTSITDPQPMGKVGMCFHPDQDRILTVRECARSQGFPDGYKFAGNIIHRHRQIGNAVP
PPLAYALGKKLREAVEKKLKDKDGQ

>Geranium_maculatum_CMT-g34989.t1
MKSTAGSGSDSGPESRSNRWVLTVCEHGDGEEQPEPFDSRKSFAKRLIPSGDGELSIAAENGGEEAYLEH
AERLSKLPSLDPAGTGNADPILKKLCGNLSARSNTSDDKSVRRSPRGAEKGILEGALAQSQGSNGLQLMQ
LSDEKELHLNVEYSADKCFRRSHVCSPAKSGSKSNCSSPIVQSMSIGKSRSPDKDCLGAVSPGTCDDLKK
RSPVEFKTKILSLSWHAEADAESMTLKYNHPSFSEHLRRSSRVIYGSSNSGANVSPRLSSSLSGAQTLDL
NMSDEQPPKKIKTSVVSPGLGALDEKQYQRSASLSSNSECIKSNSSFTVIADSSDEPPYKKIKETLGKVD
PGMMDTKFPRQSPRTAASPSDNEFDDIKCDIKSAETKSPTKNMILLSSNNKQKKYKCTFFVGDPIPSEEA
QTMDKKFSRQSLGTASCPVGEFEKTKCGSLKLENSAEKQSLTITKTSISSEHKRKSRIGSFFVGDPIPSK
EAHTMDKKFSRQSPGTASCPSVGKVGKTECCSLELQKSVEKGILTKTKTSVSSENKHKYKIGSFFVGDPV
PFEEAHTMDKKFSRQSPGIAGCPAVDKLEKTKCGSVKLQKSDEKRPPTKTKTSVSSENKQKIDSFFVGDP
IPASEAQERWHWRYELKRLHSRRSKRPSMMTKDNDDEDLIISNVECHYNQAEVNKCTYSLGDCAYIKGDG
GEKHIGRIIEFFKTTEGENYFRVQWFYKVEDTVIKEANFHNRNRLFYSTVMNDNLIDCILSKVNILQISP
KIALKSNFVLPSDFYFDTEYCVEYSTFRTMLPDDSVKSCDLSPNNHMMIPAKATATYLKNKPSCGTSEGE
LALLDLYSGCGGMSTGLCLGAKNSGVDLVTRWALDSSKSACESLKLNHPETYVRNEGAEDFLELLKEWEK
LYKRYGTNDVYIRRNSKSMDLQAVSGDANSLSEEDGDSEDEFEVSRVVDICYGDPDKTGKHGLKLKVHWK
GYGPSEDTWEPIEGLSNCHERIQDFVQKGLKSKILPLPGDVDVICGGPPCQGISGYNRFRNTDSPLTDER
NRQIIVFMDIVQFLKPKFVLMENVVDILRFDNASLGRYALSRLVCMKYQARLGIMAAGCYGLPQFRLRFF
LWGAHPSEKLPQFPLPTHDVIVRYWPPVEFERNTVAYDEDQPRDHLEKALVLEDAISDLPEVSNDESREE
MSYEKLPQTEFQRYIRSSKHEMSGSRFQGKPEEVPPIYDHRAFVLNEDNYARVCQIPKRKGANFRDLPGV
VVGKDNVARRDATIEQILLPSGKPLVPDFAFTFEQGKSKRPYARLWWDETVPTVITSPNYRSQAALHPEQ
DRILTIREYARLQGFPDYYRFCGTIPERYRQIGNAVAVPVARALGYVLGMAFQKLGGDEPLMTLPPKFSY
SNNIGCTEIDIQKPSDNEPLIPLLPTISYTNNNQSTGDYGTRETEYKAITHA

>Geranium_maculatum_CMT-g36193.t1
MASRSRSNHSGSFSVRRSPRLGETSDLSSDTPKLPLSSASRSRSKHSGSFSVRRSPRLGETSDLSSDTPI
LPLSMTSRFRSKHSGPFSVRRSPRLGETSDLSSDTPIPPLSMASRSRSKHSGSFSVRRSPRLGETSDPTA
KDEPNPAKKLKKTSSKKPETAFFVGDPVTDEEARSRWPWRYQIPNQSQSQARNSRSRDSNDSDEDDVLIL
NVKCHYYQAEVGKCIFNLGDCVYVKGEGEQNYIGKILEFFKTTDDEDYFRVQWFYRAEDTVMKEEAESHD
KRRLFYSDMMNDNLLDCIVSKLKVVQISPNVNLKSNSIPRCDFYYDMKYEVDYSTFCTMTSDCSNSTTHV
SKINHLSNNKPVFEGLASNKPCKTELTLLDIYSGCGGMSTGLCLGFKLSGLDLVTKWALDINKYACESLK
LNHPETQIRNESAEDFLGLLKEWDKLCKQYVVKNDVETPKSKVLRSTEKDQHSKTDKLLPGEFEVARLLD
VCYDDPSNTGKSGLKFLVRWLGYGPSEDTWEPLECLSGCQDRIRDFVLKGNKEKILPCPGDVDVICGGPP
CQGISGSNRFRNVDNPLEDERNQQIVVFMDIVNFLKPKYVLMENVTDILKFAKGCLGRYALSRLVHMKYQ
TRLGIMAAGCYGLPQFRLRIFLWGAHPNESLPQFPLPSHDVILRYGAPVEFERNIVAYDKGQSPILEKAL
VLSDAISDLPPVTNDEICEHMSYKDPPETEFQKYIRATKHEMMCLATNGPLKKEKALLYDHKPLPLKEDD
YSRVCLIPHRKGANFRDLPGVFVGADNVVRKDPSTEIILPSGKPLVPNYAINFRDGISRRPFGRLWWDET

VATLFTRPDPHNQVILHPEQDRVLTLREAARLQGFPDYYRFCGGMEERYCQVGNAVAVPVARALGYALGA
AVLKLSGEKPLMTLPHKFSHSTTTHLLQSSPFFPQ

>Geranium_maculatum_DNMT-g36447.t1
MGSAKVLSKAASDGVENQSSEMGKKTAPKSKSTTSDANKASSNSGAGTQPKLKRNRQESEKNVSGTRKMP
RRAAACSDFKDKTIRLSEKSSMVETKRVVVVEDEVAAISLTSGINDPRPNRRLSDFIFHDANGKAQSVEM
LEVDDLFITGVVLSLEESSGKEKGKGIRCEGFGRIESWDISGYEEGSPVIWISTDVADYDCVKPASGYKK
LYDLFFEKARLCIEVYKKVSKTAGGNPDLSVDELISQVVRSVSGSKNFPHGTSVKDFIISQGEFVYKQLT
GLDDSSAKNDLIFRDLSVLAALRDESRKHTNFAHPKAMSSGGSLKIGEGQKVNLSSSPTSGSVEDEDTKL
ARLLQEEENWRSMKPKKGQRSVASSNKFYIKINEDEIADDYPLPAYYKTTDQETDEYIIFDNDISDPNEL
PRSMLHNWSLYNSDSRLISLELLPMKTCADIDVTIFGSGIMTADDGSGFYLDSDPNQSTSGSGAAQNIDG
IPIFLSAIKEWMIEFGSSMIFISIRTDMAWYRLGKPSKQYAPWYEPVLKTARLAIGIITLLKEQSRVSRL
SFADVIRRVSEFDKNHPSYISSNPAAVERYAVVHGQIMLQQFAEYPDDAIKKSAFVTGLYDNMERKHHTK
WLVKKKMVLKKEMNMNPRAAIAPVVSKRKAMPATTTKLVNRIWGQYYSNYLPEDSKEADINEVKEEEDAE
ADADGEQEENVEEDIEEIEQKTTIDEKSKTPRASSKLAKPRRFSNDTKWDGDSVGKTSCGEVLYKQALVH
GDLVAVGGFVLVETADTDEAPPIYFVEYMFEKSDSAKMVHGRLMLRGSQTVLGNTANEREVFLTNDCLEF
ELGDVKQTVVVEIRMVPWGYQYRKANALKDKIDRERAEERKCKGLPMEYYCKSLYWPERGAFFCLPTDSM
GLGNGLCQSCKINEVEIEKEICKVNSAKSGFTYQGIEYCIHDFVYIAPHYVATDGKDQGTHKSGRNIGLK
AFAVCQVLDIDVPKASKQSNPESIKVKVRRFFRPEDASSEKPYCSDIREVYYSESMLMVSVAAIEGKCDV
RRKHDLPPSDGPVIFEHIFFCERLFDPDNGAVKQLPALIKLSSSKGKLADDAAYRKKKGKSKEGDNESDD
ANRQTDAIHKSRLATLDIFAGCGGLSEGLEKAGVSLTKWAIEYEEPAGEAFILNHPEALMFVNNCNVILR
AIMSACGDEDDCISTPEAIELAAKLGEKEISSLPRPGQVEFINGGPPCQGFSGMNRFNHSTWSKVQCEMI
LAFLSFAEYFRPKFFLLENVRNFVSFNKGQTFRLTIASLLEMGYQVRFGILEAGSYGVSQSRKRAFIWAA
SPEEILPEWPEPMHVFASAELKISLPKNTHYAAVRSTATGAPFRAITVRDTIGDLPPVGNGASLTIMEYK
NEPVSWFQKKIRGDMLVLNDHISKEMNELNLIRCQKIPKQPGSDWRDLPDEKVRLSTGQLVDLIPWCLPN
TAKRHNQWKGLFGRLDWEGNFPTSITDPQPMGKVGMCFHPDQDRILTVRECARSQGFPDSYKFAGNIQHK
HRQIGNAVPPPLAYALGTKLKEAVEKKRSEGN

>Geranium_maculatum_DRM-g39211.t1
MQCVVGCELYCLDFTSFDMGEIPDSKISLDLEVERAIAPKAEPEILDFVLPYDNMWPRGVGDNVASSSGS
NLRSSLIGMGFLPSLVDKAIEEKGEDDADLLVETLFAYSDLHKSDAESSDSLDGLFDDKDANSPPEVSAV
EPKEEPDVFDGINEDKRASLLMMNFSINEVDFAIDKLGEDAPIDEIVDFVIAAQIAEKYEKKVEGTTLGI
EESNEDVVNETLYGTMDKTLCLLQMGFTENQVSAAIEKFGPDVPISELADAIFAGQIADDYLEKDKCGSS
GDGSRHRPYGSVRVKTEDPLREDFNEELNRKGKIPKQECFDDNLDASSLFRVSDFDCIRKGKRLKQDYLD
DSSSCHKTARLGEKVSRTSTRYEVPSPFKSGPCKTLDRMVAKPPYFFYGSVVNISSDSWAKVSQFLYAVV
PEFVNAQFFSALIRKEGYVHNLPTENRFHIIPKPPMTIQDAVPHTKRWWPSWDTRKQLSCITSENSGISQ
LCDRLGRMLNDSRGLLSTEQQKDILHHCRALNLVWVGRHKLGPIEPEHLECILGYPLNHTQDAGISLIER
LEALKHCFQTDTLGYHLSVLKSMYPDGLTMLSIFGGIGGAEVALHQLGIRLKGVVSVETSVTKRNILKRW
WESTSQDGELVQLEDIEKLTSSRLQSLAERFGGFDFVICQSPCSSSSLTPKVAVADTDSLPGFDFSLFYE
FVRVLQRVRSMPERRR

>Geranium_maculatum_CMT-g39809.t1
MAGKRGSESASATRSSKRASSKKRKLAEEELELEEDLEGQFDEEAVQAIEEGGAAAKKLSEEGEVKVRES
VVAARVSPRKARVHEKEEDEPESRFVGSAVPDAEARRRWPHRYAGKNQKKPDELKYLNMDNSEGILQSRR
HFKKAEVDGHIIFNLYDDAYVQGGEGEDNPICKIIEMFEAVDGALYFTAQWFYRAKDTVIKNHDTLIDPK
RVFYSEVMDDNPLDCLVEKLHIVKVSLNIDLEAKKNSIPPCDYYYDMLYLLPYSTLIKAPTDNKTSSETS
SSMSSEVVINDTSETKCGSTKVCQVGQSRKPEFTLLDLYAGCGGMSTGLCLGANLSGVNLVTKWAVDLNK
FACESLRFNHPETEVRNEAAEDFLSLIKEWEKLCISFSLVSRDDKQPYVDPYAMEEEKEDSENDEEDDEG
ETDPEVFEVEKILEACFGDPNETGKRQMYFQVRWKGYGPDHDTWEPLEGLSGCRERMKEFVTQGFKSKTL
PLPGAVDVICGGPPCQGVSGFNRFRNKEDPLADEKNKQLLVFMEIVEYLKPKFVLMENVVDLIKFAGGYL
GRYALGRLVQMNYQARMGMMAAGAYGVPQFRMRVFLWGARPTEKLPQYPLPTHDVVIRGVIPLEFEKLLV
AHDDKEKVVLEHKLCLKDAFSDLPAVENDEQRDEMDYGKDPETEFQRLIRLSKDELMGVSTQQEPSAKKL
YDHRPLQLNTDDYQRSCRIPKHKGANFRHLPGVLVGTDNKVKWDPDVPRELLASGKPLVPDYAMSFVKGS

SSKPFARLWWDEIVPTVVTRAEPHNQAILHPVQDRVLTVRENARIQGFPDYYKLCGTIKERYIQVGNAVA
VPVARALGYSLGLAFQGKIGEEPLMKLPSKFPNFIEQN

>Geranium_maculatum_DRM-g39905.t1
MDGNMSGGEDDIDWNTDDELEVASYPVSSSSKLLLRGGGPSVGFAEASCSTWPASSSNSMANDFRMMGFP
ENLIAKAIEENGEANADTILNTLLTYQAIENSPEGQEPIDPDLPEKQETVDSDHWSSDNESLLDEASDID
GSSEDEDYCPETTNIQKIKEEITDGSSETDNRLVLLMDMRYTREEASIAIQRCGADSSISELVDFIYAAQ
MSKAADFDLVDDPEVKPTPYLGTKRRKFLNNLLKRKKPRGLEKKIPNEDQETLRLPNPMIGFGIPGELCL
DRNLPIPDAAIGPPFFYYENVALASKGAWNTISRHLYEIEPEFVDSKHFCAAARKRGYIHNLPVVDRFPL
VPLPPRTIHDAMPLTRKWWPSWDKRTKLNCLQTCTASATTTDRIRKSLEVWDGDPPHHIQKYVLDECRKW
NLVWVGKHKVAPLEPDEIEMLLGFPKNHTRGMSRTNRVKSLGNSFQIDTVAYHLSVLKERFPGGINLLSL
FSGIGGAEVALHRLGIPLKNVVSVEISEASRETVRSWWTSTEQKGNLIHIEDVQTLNGDRLDEMMSSFGG
FDLVVGGSPCNNLAGSNRHSRDGLEGEHSVLFFDYVRILNYVMSSRR

>Geranium_maculatum_DRM-g3909.t1
MDGNVPSGENDIDWNTDDELEISNHPVSSSSSLSLPGGEATVGSLEASCLEGPSNTIVRDFLGMGFSQKM
ISRAIEENGEGDADSILNTLLTYQAIENSPGQKQPIDLDIPKKQQTVDSDGCSSGYGSFVGDFSDIDSFS
ENEEFHAAATDIHKIKKEIMDISSETNHNLLFLVDMEFTPEEAAIAIQRCGPDSSIDELVDFIGAARMAK
AADAHFVDLPEVKPTAYLGSKRRKHLNYDLLKRKKARGLNEEDETIRLPNPMIGFGTPGELCPAVNRPLP
EAAIGPPYFYYENVALAPKGVWNTISRFLYDVEPEFVDSKYFCAAARKRGYVHNLPVVNRYPLVPLPPRT
IHDALPMTRKWWPSWDTRTKLNCLQTCIASAKLSDRIRKALEDWDGEPPLHVQKYVMDECRKWNLVWVGR
NKVAPLEPDEVEMLLGFPKNHTRGGGISRTDRYKSLGNSFQVDTVAYHLSVLKDMFPGGINMLSLFSGIG
GAEVALHRLGIPLKNVVSVEISEVNRNIVRSWWEQTNQKGNLIDIADVQTVNGDRLEQLMNSFGGFDLVV
GGSPCNNLAGSNRHSRDGLEGKQSSLFYDYFRILDLVKCVMNRGI

>Geranium_maculatum_DNMT-g4436.t1
MPNQALVSVGTKRETKVEDEFEAIRLTHGLDEYRANRRLTDFIFHDVNEEPQPVETLEVKSLFISGCILP
LEGSSDQEKEKGVRCECFGRVESWAISGYDEGAPVIWVSTAVADYECVDPGKEYEMLYRVFFEKARACIE
IYTTLSSRRTPDLSVDELLARVVRSMSNSKSFTCEAEIKDFIVSKGEFIYNELVGLDDTPETNNLMFRDL
PVLNALRDDARRHRDSVPPEAISCGMLTLSRGVDDTSHANGAAEGVDATLARLLQEQDYRRSSRQKKGQC
RDADLSSYAGGAEEDDDAALARLLQEQEYRRSSRQKKGIAKESEGLFKFAASQQNQKRAKPKRTESPFGS
PNINMPKKRQVRVHHEVKAITMTSEEDDPRPNRRLTNFVFHDAEGNPQALETLEAKFLFISGLILPLEEI
SEKQKEMGVRCKSFGHVESWAISGYEEGSPVIWISTEAADYECVNPAKGYEKQYNVFFEKARACVEVYMK
LSRACGGNPNLSLDEMLVRVAQSMSNSENFTCEAEVKKFIGSQGEFIYNELIGLDEMSCDEKLGDLPVLI
ALKDNAKNYERFVPPILKVSDEKQINDPLSSAFGADEDEELKSARFMLEEENWRSTIQKRVDHEVEAINL
TCGQEDGPQSNRRVTNFTFHDADGKPRCLEKLEVDVLYTSGLILPLEESCEKEKQIGVRCEGFGRIESWA
ISGYEEGSPIIWISTEVADYECVSPAKGYEKLYNILFEKARACIEVYMKLSLTRGGDPDLSLDTLLARVV
QSMSTEDFTNEFDITDFVISHGEFIYNELVGLDETSSDQMFRDLPVLVSLAKEAKKHRPSVPKRARSRKF
GDGEEIRQNGSSACGREEDEDEKSCGAEELPRSMLQNWSLYNSYSKLISLELLPMKSCSDDSYTIFGSGV
MTVDDDGSGLSLDDDSNLLSPASAGAQNVDGIPIYLSTIREWMVEFKSSMIFVSIRTDTCWYRLGKPSKQ
YAPLYEQVLKTAKLGVSIILLLKEQTRVARLSFADVMRRISQFRKNHPTYISGDPAEVEMHVLAHGQMIL
RQFAAYPEDVISRCAFVTSLRDKMVEKDNLKWLAKKRTILNNNLAETEATSPLILETKAMPATKTGLVNR
IWREHYSSQFPEDSDEVDVRWDGESVGKTSSGNDLYKRAIVCGHLVTVGGYVEMEGLDDDTTIYFVEYIY
ENSDSRKMFHGRLMLRGSQTVIGNAGNEREVFLTNECQDLELCNVQEPVSVEIKTMPWGHQHRKANADKS
RIDSERAKKRKRKRLSTEYYCKSLYWPGRGAFFCLPTDTMGLGNGACRSCVMKETEKEKEVFKVDPSLSR
FVYKGTEYCIHDFVYVAPHHFATDEKVHDKLKGCKNVGLKAYVICHLLEIKVADGSEEAYPKYTCVKVRR
FFRPEDISASKAYGSDIREVYYSEQILTVPVIVIEGKCEVRKKSDLQSLDGSVAFGHTFFCEHLYDPHNG
SFEELQSHITLGSSKEKQQMDTFCRNPLATLDIFAGCGGLSEGLQRAGASLTKWAIEYEEPAGEAFSLNH
PEALIFINNCNVILRAIMSACGDADDCISTSEADELAAKLDEKVIKNLPRPGQVEFINGGPPCQGFSGLN
RFSQSRHSKVQCEMILAYLSFADYFRPRFFLLENVRNFVSFDKGKTFRLTLALLLEMGYQVRFGILDAGA
YGLPQSRRRVFIWAASPEETLPEWPEPMHVFGGRELKVTLSESSQYAAVRSTEGGAPFRSITVRDAIGDL
PVVENGASAITMQYENKPVSWFQKQMQGDEKILNDHISKSMNELNFIRCQRIPKRPGADWRDLPMEEVEL
STGLRSKLVPSWVPNTAQRHNHWKGVLGRLCWEGNFSTSLTDPQPEGIVGMCFHPDQDRILTVRECARSQ
GFPDSYKFSGNIQHKHKQVGNAVPPPLAYALGWMLKEAVGKKHSH

>Geranium_maculatum_DNMT-g6028.t1
MGSAKVVGKSASDGVDNQSSGVGKKTATKLKSTASDDKKNKTNQKRSRLESNQNIPGSRKMPKRAAACLD
FKDKTVRLSEKSSTVETKRDIVVEDEVAAISLTSGINDPRPNRRLTDFIFHDADGKAQSVEMLEVDDLFI
TGVILSLEENSDKEKGKGVRCEGFGRIESWDISGYEEGSPIIWISTEFADYDCVKPATSYKKLYDLFFEK
ACLCIEIYKKLSKTSGGNPDLSVDELISQVVRSMSGSKNFPHGTSVKDFIVSQGEFVYNQLTGLDETSGK
NDLIFRDLPVLAALKDESKKHMGFAPPKAMSSGGSLKIGEREKANQCNSPTSGVVDDEDMKLSRLLQEEE
NWRSMKQKKGQRSAALSNKFYIKINEDEIADDYPLPAYYKTTTEETDEYIIFDNDICDPNELPRSMLHNW
SLYNSDSRLISLELLPMKPCADIDVTIFGSGFMTADDGSGFCLDSDPNQSSSGSCEVQNNDGIPIYLSAI
KEWMIEFGASMIFISIRTDMAWYRLGKPSKQYAPWYEPVLKTARLAIGIITLLKEQSRVSRLSFADVIKR
VSEFDKNHPAYVSSTPAVVERYAVVHGQIMLQQFAEYPDDAIRKSSFVTSLHDNMERKHHTKWLVKKKMV
LKKEGNMNPRAAMAPLVSKRKTMPATTTKLINRIWGRYYSNYLPEDSKDADINEVKEDEDVDADGEQEEN
VEEDIEETDQKTIADKIEAPRASPKLSIARRSSNDIKWDGDSVGKTSCGEVLYKQAIVHGDLVAVGGCVS
VETTDTDDLPLIYFVEYMFEKSDATKMIHGRLLLRGSQTVLGNTANEREVFLTNDCLEFELGDVKQNEVV
EIRLTPWGYHHRKANALKEKIDRERAEERKSKGLPMEYYCKSLYWPDRGAFFCLPTDTMGLGNGLCQSCE
INKSEAEKEICSLNSARSAFTYKGTEYFIHDFAYIAPHYVATDEKDQGTHKSGRNIGLKAYTVCQLLDID
APKASKKANPESIEVKVRIFFRPEDISSEKSYCSDIREVYYSETMLMVPLAAIEGKCDVRRKHDLPPRKH
DLPPVIFEHIFFCERLFDPDSGAIKQMPAQVKLRSSKGKLSDDAAYRKKKGKGKEGENMGDDTDRKTDAP
HGNRLATLDIFAGCGGLSEGLEKAGVSLTKWAIEYEEPAGEAFTLNHPEALMFVNNCNVILRAIMSACGD
EDDCISTTEAIELAAKLNEKEISNLPRPGEVEFINGGPPCQGFSGMNRFNHSTWSKVQCEMILAFLSFAE
YFRPKFFLLENVRNFISFNKGQTFRLTIASLLEMGYQVRFGVLEAGAFGVSQSRKRAFIWAASPEEILPE
WPEPMHVFASAELKIALSKNTHYAAARSTANGAPFRAITVRDTIGDLPAVENGASKATMEYKNEPISWFQ
KKTRGDMMVLNDHISKEMNELNLIRCQRIPKVPGSDWRDLPDEKVKLSTGQLVDLIPWCLPNTAKRHNQW
KGLFGRLDWEGNFPTSITDPQPMGKVGMCFHPDQDRILTVRECARSQGFPDSYKFAGNIIHRHRQIGNAV
PPPLAYALGTKLKEAVEKKLKDKDGQ

>Geranium_maculatum_DRM-g10748.t1
MDGNVPSGENDIDWNTDDELEISNYPVSSSSSLSLPGGEATVGSLEASCLAGPSNTMVRDFLGMGFSQKM
ISSAIEENGEGDADSILNTLLTYQVIENSPGQKQPIDPDIPKKQQTVDSDGCSSGYDSFVGDFSDIDSFS
ENEEFHAAATDIHKIKKEIMDISSETNHNLLFLVDMEFTPEEAAIAIQRCGPDSSIDELVDFIGAARMAK
AADAHFVDLPEVKPTAYLGSKRRKHLNYDLLKRKKARGLNGGLNEEDETIRLPNPMIGFGTPGELCPTVN
RPLPEAAIGPPYFYYENVALAPKGVWNTISRFLYDVEPEFVDSKYFCAAARKRGYVHNLPVVNRYPLVPL
PPRTIHDALPMTRKWWPSWDTRTKLNCLQTCIASAKLSDRIRKALEDWDGEPPLHVQKYVMEECRKWNLV
WVGRNKVAPLEPDEVEMLLGFPKNHTRGGGISRTDRYKSLGNSFQVDTVAYHLSVLKDMFPGGINMLSLF
SGIGGAEVALHRLGIPLKNVVSVEISEANRNIVRSWWEQTNQRGNLIDIADVQTVNGDRLEQLMNSFGGF
DLVVGGSPCNNLAGSNRYSRDGLEGKQSSLFYDYFRILDLVKCVVNRGI

>Geranium_maculatum_DRM-g12670.t1
MQCVVGCELHCLDFTSFDMGEIPGSKISLDHEVERAIAPKAEPEILDFVLPSGNMWPRGVGDNVASSSGS
NLRSSLIGMGFLPSLVDKAIEEKGEDDADLLVETLFAYSDLHKSNAESSDSLDGLFDDKDANSPPEVSTV
EPKEEPDVFDGIIEDKRASLLMMNFSINEVDFAIDKLGEDAPINEIVDFVIAAQIAEKYEKEVEGTTLGI
EENNEDVVNETLYGTMDKTLCLLQMGFTENQVSAAIEKFGPDVPISELADAIFAGQIADNYLEKDKKCGS
SGDGSRHHPYGSVRVKTEDPLRGDFNEELNRKGKIPKQECFDDNLDASSLFRVSDFDCTRKGKRLKQDYL
DDSSSCHKTARLGEKVSRTSTRYEVPSPFKSVPCKSLDRMVAKPPYFFYGSVVNISSDSWAKVSQFLYAV
VPEFVNAQFFSALIRKEGYVHNLPTENRFHIIPKPPLTIQDAIPHTRRWWPSWDTRKQLSCITSENSGIS
QLCDRLGRMLNDSRGLLSTEQQKDILHHCRTLNLVWVGRHKLGPIEPEHLECILGYPLNHTQDAGISLIE
RLETLKHCFQTDTLGYHLSVLKSMYPDGLTMLSIFGGIGGAEVALHQLGIRLKGVVSVETSVTKRKFLKR
WWESTSQDGELVQLEDIEKLTSSRLQSLAERFGGFDFVICQSPCSSSSVTPKVAVADTDSLPGFDFSLFY
EFVRVLQRVRSMPERR

>Geranium_maculatum_CMT-g26781.t1
MAGKRGSESSAATRSSKRASSKKRKLADEELEVEEDLEGQFDEEAVQAIEDGAAVKKLSEEGEVKVRESV
VAARVSPRKARVHEKEEDEPESRFMGGAVPDAEARRRWPHRYAGKKKPDELKYLNMDNSEGILQSRRHFK
KAKVDGHIIFNLYDDAYVQGGEGEDNPICKIIEMFEAVDGALYFTAQWFYRAKDTVIKNHGTLIDPKRVF
YSEVMDDNPLDCLVEKLHIVKVSLNIDLEAKKNSIPPCDYYYDMLYLLPYSTLIKAPTDNKTSSETSSSM

SSEVVINDTSETKCGSTKVCQVGQSCKPEFTLLDLYSGCGGMSTGLCLGANLSGVNLVTKWAVDLNKFAC
ESLRFNHPETQVRNEAAEDFLSLIKEWEKLCISFSLVSRDDKQPYVDPYAMEEEKEDSENDEEEEDEGET
DPEVFEVEKILEACFGDPNETGKRQMYFLVRWKGYGPDHDTWEPLEGLSGCRERMKEFVTQGFKSKTLPL
PGAVDVICGGPPCQGVSGFNRFRNKEDPLADEKNKQLIVFMEIVEYLKPKFVLMENVVDLIKFAGGYLGR
YALGRLVQMNYQARMGMMAAGAYGVPQFRMRVFLWGARPTEKLPQYPLPTHDVVIRGGVPLEFEKLVVAH
DDKEKVVLEHKLYLKDAFSDLPAVENDEQRDEMDYGKDPETEFQCLIRLSKDELLGVSTQPEPSVKKLYD
HRPLQMNTDDYQRSCRIPKHKGANFRHLPGVLVGSDNKVKWDPDVPRELLASGKPLIPDYAMSYKKGSSS
WPFARLWWDEIVPTVVTRAQPHNRAILHPVQNRVLTVRENARIQGFPDYYKLCGSIKERYIQVGNAVAVP
VARALGYSLGLAFQGSIGEEPLMKLPSKFPNFIEQNCESSEDNV

>Geranium_maculatum_DNMT-g33211.t1
MGSAKVLSKAASDGVANQSSGMGKKTAPKSKSTTSDANKASSNSGAGTQPKLKRNRQESEKNVSGTRKMP
RRAAACSDFKDKTILLSEKSSMVETKRVVVVEDEVAAISLTSGINDPRPNRRLSDFIFHDTNGKAQSVEM
LEVDDLFITGVVLSLEESSDKEKGIRCEGFGRIESWDISGYEEGSPVIWISTDVADYDCVKPASGYKKLY
DLFFEKARLCIEVYKKLSKTAGGNPDLSVDELISQVVRSLSGSKNFPHGTSVKDFIISQGEFVYKQLTGL
DDSSTRNDLIFRDLPVLVALRDESRKHTNFAQPKAMSSGGSLKIGEGQKVNQSSSPTSGSVEDEDTKLAR
LLQEEENWRSMKPKKGQRSVASSNKFYIKINEDEIADDYPLPAYYKTTDQETDEYIIFDNDISDPNELPR
SMLHNWSLYNSDSRLISLELLPMKTCADIDVTIFGSGIMTADDGSGFYLDSDPNQSSSGSGAAQNIDGIP
IFLSAIKEWMIEFGSSMIFISIRTDMAWYRLGKPSKQYAPWYEPVLKTARLAIGIITLLKEQSRVSRLSF
SDVIRRVSEFDKNHPSYISSNLAAVERYAVVHGQIMLQQFAEYPDDAIRKSAFVTGLYDNMERKHHTKWL
VKKKMVLKKEMNMNPRAAIAPVVSKRKAMPATTTKLVNRIWGQYYSNYLPEDSKEADINEVKEEEDAEAD
ADGEQEENVEEDIEEIEQKTTIDDKSKAPRASSKLAKPRRFSNDTKWDGDSVGETSCGEVLYKQALVHGD
LVAVGGFVLVETADTDEAPPIYFVEYMFEKSDSTKMVHGRLMLRGSQTVLGNTANEREVFLTNDCLEFEL
GDVKQTLVVEIRLVPWGYQYRKANALKDKIDRERAEERKCKGLPMEYYCKSLYWPERGAFFCLPADSMGL
GNGLCQSCKINEVEIEKEICKVNSAKSGFTYQGIEYCIHDFVYIAPHYVATDGKDQGTHKSGRNIGLKAF
AVCQVLDIDLPKASKQSNPESIKVKVRRFFRPEDASSEKPYCSDIREVYYSESMVMVPVAAIEGKCDVRR
KHDLPPSDGPVIFEHIFFCERLLDPDNGAVKQLPALIKLSSSKGKLADDAAYRKKKGKSKEGDNESDDAN
RQTEHKSRLATLDIFAGCGGLSEGLEKAGVSLTKWAIEYEEPAGEAFILNHPEALMFVNNCNVILRAIMS
ACGDEDDCISTPEAIELAAKLGEKEISSLPRPGQVEFINGGPPCQGFSGMNRFNHSIWSKVQCEMILAFL
SFAEYFRPKFFLLENVRNFVSFNKGQTFRLTIASLLEMGYQVRFGILEAGSYGVSQSRKRAFIWAASPEE
ILPEWPEPMHVFASAELKISLSKNTHYAAVRSTATGAPFRAITVRDTIGDLPPVGNGASLTTMEYKNEPV
SWFQKKIRGDMLVLNDHISKEMNELNLIRCQKIPKQPGSDWRDLPDEKVRLSTGQLVDLIPWCLPNTAKR
HNQWKGLFGRLDWEGNFPTSITDPQPMGKVGMCFHPDQDRILTVRECARSQGFPDSYKFAGNIQHKHRQI
GNAVPPPLAYALGTKLKEAVEKKRSEGH

>Geranium_maculatum_CMT-g33484.t1
MTSDCSNSTTHVSKINHLSNNKPVFEGLASNKPCKTELTLLDIYSGCGGMSTGLCLGFKLSGLDLKWALD
INKYACESLKLNHPETQIRNESAEDFLDLLKEWDKLCKRYVVKNDVETPKSKVLRSTEKDQHSKTDKLLP
GEFEVVRLLDVCYGDPSNTGKLGLKFQVRWLGYGPSEDTWELLECLSGCQDRIRDFVLKGNKEKILPHPG
VDVICGGPPCQGISGFNRFRNVDNPLEDERNQQIVVFMDIVNFLKPKYVLMENVTDILKFAKGCLGRYAL
SRLVHMKYQTRLGIMAAGWYGLPQFRLRIFLWGAHPNERLPQFSLPSHDVILRYGAPVEFERNIVAYDEG
QSPILEKALVLSDAISDLPPVTNDEIREHMSYKDPPETEFQKYIRATKHEMTCLATNGPSKKEKALLYDH
KPLPLNEDDYSRVCRIPHRKGANFRDLPRVFVGADNVVRKDPSTEIILPSGKPLVPDYAINFRDGISRRP
FGRLWWDETVATRFTRPDPHNQAILHSEQDRVLTLREAARLSRLLQILWRNGRQVLSGRECGGCSGGASA
RICAGGGGSQA

Supplementary Methods S3. Representative gene module selection.

To test the association between the gene modules generated by the weighted gene co-expression network analysis (WGCNA, Langfelder and Horvath 2008) and the experimental treatments, module gene significances were calculated by transforming each module gene's FDR as $-\log_{10}$(FDR) and calculating its mean per module. Given there are four comparisons between control vs. 5-azaC treatment (all samples, juvenile roots, juvenile leaves, and adult leaves), each module showed four module gene significance values. Gene module memberships from modules with the highest gene significances were calculated as the resulting Pearson's correlation coefficient from correlations between gene expression and its relevant module eigengenes. Modules M10 and M6 showed the highest gene significance values when considering control vs. 5-azaC adjusted p-values (**Fig. 5** in the main text), being particularly associated with increased expression in samples treated with 5-azaC. Module M10, however, showed a high proportion of genes excluded by *DESeq2* in the 5-azaC comparison due to excess of zero count samples, extreme outliers, or filtering due to low mean normalized counts (which increased the error associated to gene significance estimates) and it did not show any enriched GO terms precluding us to deeply understand its functional meaning. Thus, module M6 was considered as the most informative module (see **Supplementary Fig. S7**).

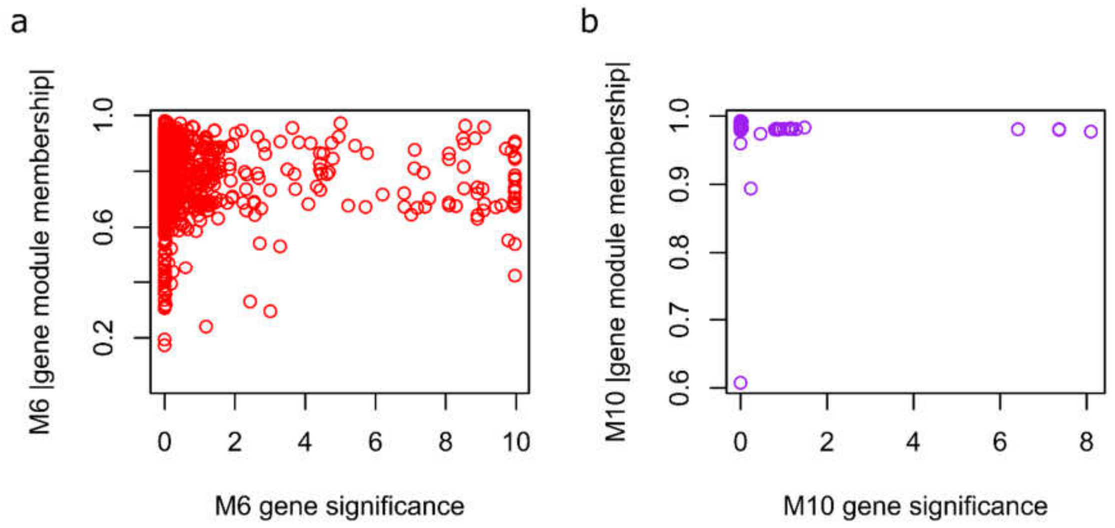

Supplementary Fig. S7. Gene significances and gene module memberships in the two 5-azacytidine associated modules M6 (**a**) and M10 (**b**). Each plot shows gene significances (negative logarithm to the base 10 of the false discovery rate) in the X-axis and the absolute value of gene module membership (Pearson's correlation coefficients between the gene's expression and the module eigengenes). The dots in the top right corner of these plots show genes whose expression is both highly significant different between control vs 5-azaC and highly correlated with the eigengene values from the module. Module M6 (red) shows a higher number of genes in the top right corner, meaning that the module's gene significance is better supported that in M10 (purple).

Supplementary Results S1. Differential gene expression between tissues and developmental stages

There were tens of thousands of DEGs in the comparisons between tissues and between developmental stages (54,424 and 37,102, respectively, **Supplementary Fig. S3, Supplementary Tables S1 and S2)**. In the juvenile leaves and juvenile roots comparison, genes up-regulated in leaves showed GO terms related to photosynthesis processes and chloroplast localization; while those upregulated in roots showed GO terms related to cell wall, ribosomal activity and excretion **(Supplementary Table S8)**. In the juvenile leaves and adult leaves comparison, genes up-regulated in the juvenile stage were enriched in GO terms related to abscisic acid signaling pathway, stomatal movement regulation and response to stress; while adult leaves showed enriched GO terms related to ribosomal activity from mitochondria, cytosol, and chloroplast **(Supplementary Table S9)**. Hypergeometric test showed that the overlap of the DEG sets from the juvenile leaves vs juvenile roots, and juvenile vs adult leaves comparisons, was not greater than expected by chance in any case (P-value > 0.05).

List of References

Andrews SR. 2010. *FastQC: a quality control tool for high throughput sequence data*. https://www.bioinformatics.babraham.ac.uk/projects/fastqc/

Consortium U. 2007. The universal protein resource (UniProt). *Nucleic Acids Res*. 36:D190-D195. doi: 10.1093/nar/gkm895.

Flynn JM, Hubley R, Goubert C, Rosen J, Clark AG, Feschotte C, Smit AF. 2020. RepeatModeler2 for automated genomic discovery of transposable element families. *Proc Natl Acad Sci USA.* 117(17): 9451-9457.

Gurevich A, Saveliev V, Vyahhi N, Tesler G. 2013. QUAST: quality assessment tool for genome assemblies. *Bioinformatics.* 29(8): 1072-1075.

Haas BJ et al. 2013. De novo transcript sequence reconstruction from RNA-seq using the Trinity platform for reference generation and analysis. *Nature Protocols.* 8(8): 1494-1512.

Hufnagel DE, Hufford MB, Seetharam AS. 2020. SequelTools: a suite of tools for working with PacBio Sequel raw sequence data. BMC *Bioinformatics.* 21(1): 429.

Jones P et al. 2014. InterProScan 5: genome-scale protein function classification. *Bioinformatics.* 30(9): 1236-1240.

Kolmogorov M, Yuan J, Lin Y, Pevzner PA. 2019. Assembly of long, error-prone reads using repeat graphs. *Nat Biotechnol.* 37(5): 540-546.

Laetsch DR, Blaxter ML. 2017. BlobTools: Interrogation of genome assemblies. *F1000Research.* 6:1287.

Langfelder P, Horvath S. 2008. WGCNA: an R package for weighted correlation network analysis. BMC *Bioinformatics.* 9(1): 559.

Manni MM, Berkeley R, Seppey M, Simão FA, Zdobnov EM. 2021. BUSCO Update: Novel and Streamlined Workflows along with Broader and Deeper Phylogenetic Coverage for Scoring of Eukaryotic, Prokaryotic, and Viral Genomes. *Molecular Biology and Evolution.* 38(10): 4647-4654.

Marcais G, Kingsford C. 2011. A fast, lock-free approach for efficient parallel counting of occurrences of k-mers. *Bioinformatics.* 27(6): 764-770.

Marcais G, Yorke JA, Zimin A. 2015. QuorUM: An Error Corrector for Illumina Reads. *PLoS One.* 10(6): e0130821.

Munoz-Merida A, Viguera E, Claros MG, Trelles O, Perez-Pulido AJ. 2014. Sma3s: a three-step modular annotator for large sequence datasets. *DNA Res.* 21(4): 341-353.

Pertea G, Pertea M. 2020. GFF Utilities: GffRead and GffCompare. *F1000Res.* 9.

Simão FA, Waterhouse RM, Ioannidis P, Kriventseva EV, Zdobnov EM. 2015. BUSCO: assessing genome assembly and annotation completeness with single-copy orthologs. *Bioinformatics.* 31(19): 3210-3212.

Smit A, Hubley R, Green P. 2021. 2013–2015. RepeatMasker Open-4.0.

Stanke M, Keller O, Gunduz I, Hayes A, Waack S, Morgenstern B. 2006. AUGUSTUS: ab initio prediction of alternative transcripts. *Nucleic Acids Res.* 34(Web Server issue): W435-439.

Vurture GW, Sedlazeck FJ, Nattestad M, Underwood CJ, Fang H, Gurtowski J, Schatz MC. 2017. GenomeScope: fast reference-free genome profiling from short reads. *Bioinformatics.* 33(14): 2202-2204.

Zimin AV, Marcais G, Puiu D, Roberts M, Salzberg SL, Yorke JA. 2013. The MaSuRCA genome assembler. *Bioinformatics.* 29(21): 2669-2677.

Zimin, AV, Salzberg SL. 2020. The genome polishing tool POLCA makes fast and accurate corrections in genome assemblies. *PLoS Comput Biol.* 16(6): e1007981.
