# Supplementary material for: Understanding epigenetic regulation in nonmodel plants: transcriptomic responses to seed demethylation in leaves and roots of the annual herb Erodium cicutarium (Geraniaceae)
Source: G3 (Bethesda). 2026 May 15;16(8):jkag129. doi: 10.1093/g3journal/jkag129 (PMC13439943; doi:10.1093/g3journal/jkag129)
Supplement: Supplementary file 1 [file jkag129_supplementary_data.zip › Supplementary_Figure_S1_G3-2026-406718.pdf]

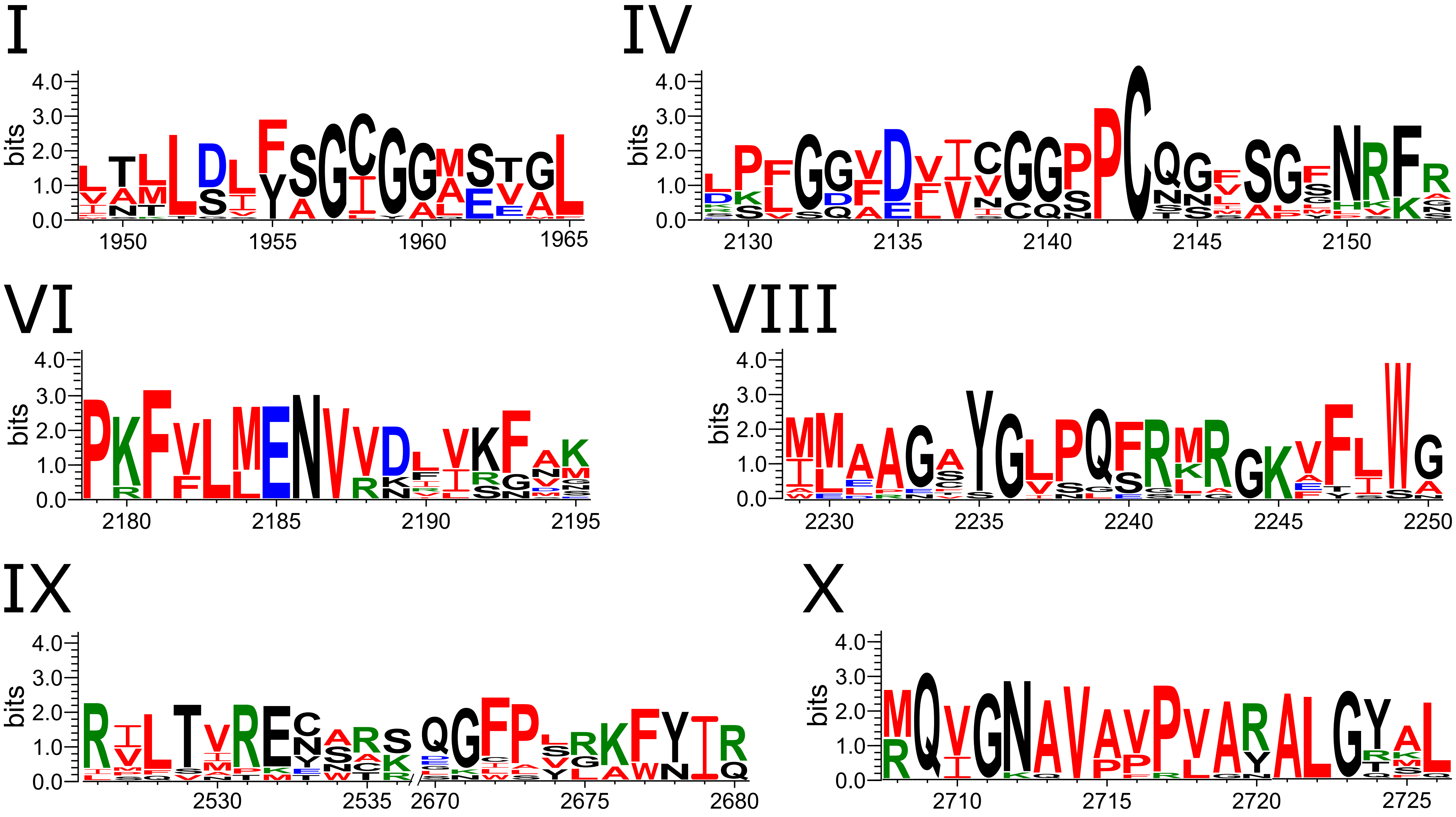
I
IV
VI
VIII
IX
X
bits
4.0
3.0
2.0
1.0
0.0
1950
1955
1960
1965
2130
2135
2140
2145
2150
2180
2185
2190
2195
2230
2235
2240
2245
2250
2530
2535
2670
2675
2680
2710
2715
2720
2725
