# Supplementary material for: Understanding epigenetic regulation in nonmodel plants: transcriptomic responses to seed demethylation in leaves and roots of the annual herb Erodium cicutarium (Geraniaceae)
Source: G3 (Bethesda). 2026 May 15;16(8):jkag129. doi: 10.1093/g3journal/jkag129 (PMC13439943; doi:10.1093/g3journal/jkag129)
Supplement: Supplementary file 1 [file jkag129_supplementary_data.zip › Supplementary_Figure_S2_G3-2026-406718.pdf]

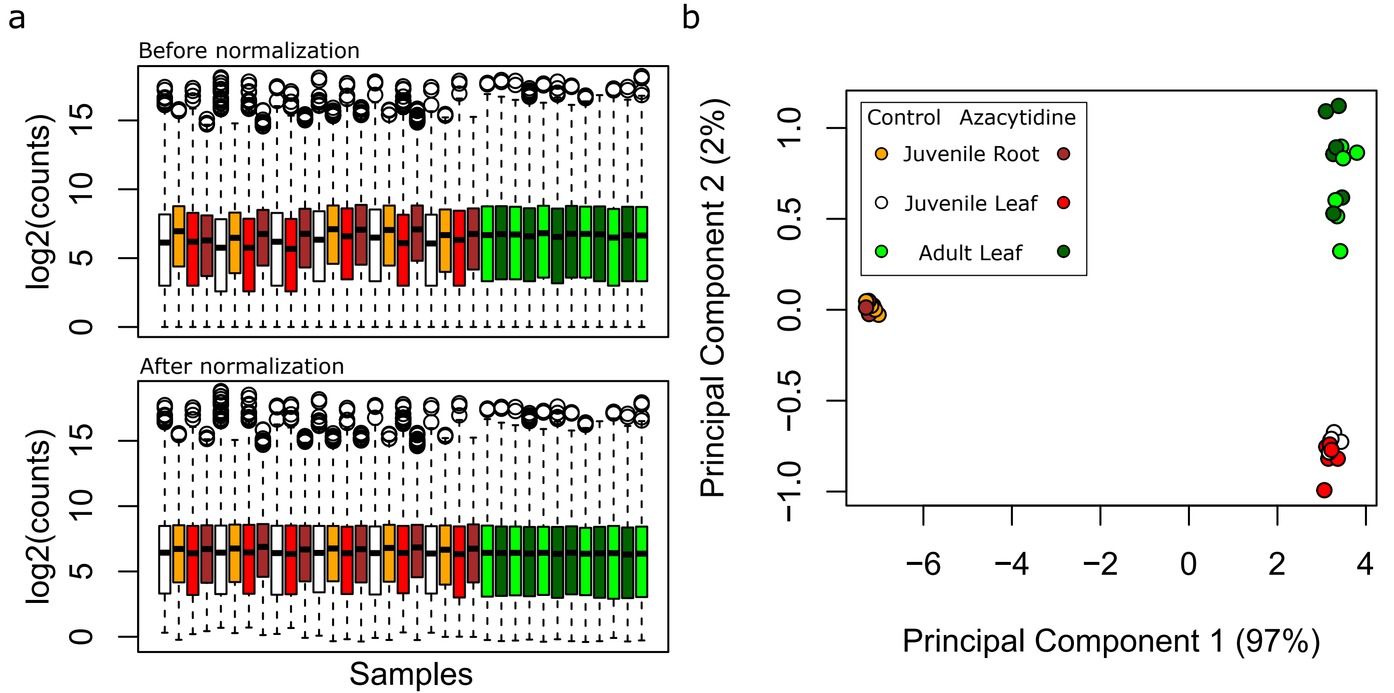

a
Before normalization
After normalization
log2(counts)
0
5
10
15
Samples
b
Principal Component 2 (2%)
Principal Component 1 (97%)
Control
Azacytidine
Juvenile Root
Juvenile Leaf
Adult Leaf
−1.0
−0.5
0.0
0.5
1.0
−6
−4
−2
0
2
4
