# Supplementary material for: Understanding epigenetic regulation in nonmodel plants: transcriptomic responses to seed demethylation in leaves and roots of the annual herb Erodium cicutarium (Geraniaceae)
Source: G3 (Bethesda). 2026 May 15;16(8):jkag129. doi: 10.1093/g3journal/jkag129 (PMC13439943; doi:10.1093/g3journal/jkag129)
Supplement: Supplementary file 1 [file jkag129_supplementary_data.zip › Supplementary_Figure_S3_G3-2026-406718.pdf]

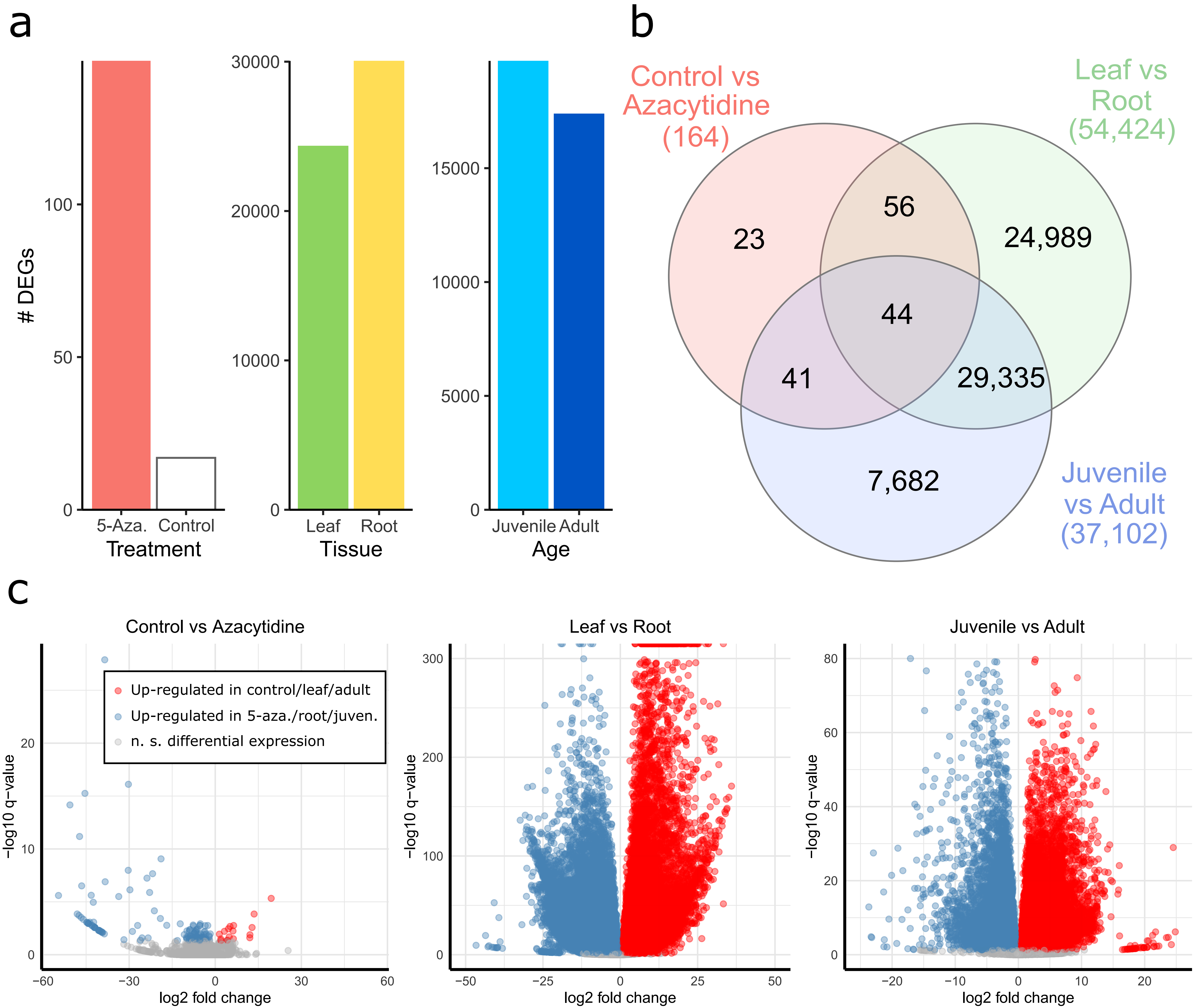
a
DEGs
100
50
0
5-Aza.
Control
Treatment
30000
20000
10000
0
Leaf
Root
Tissue
15000
10000
5000
0
Juvenile
Adult
Age
b
Control vs Azacytidine (164)
Leaf vs Root (54,424)
Juvenile vs Adult (37,102)
23
56
24,989
44
41
29,335
7,682
c
Control vs Azacytidine
Leaf vs Root
Juvenile vs Adult
Up-regulated in control/leaf/adult
Up-regulated in 5-aza./root/juven.
n. s. differential expression
−log10 q−value
log2 fold change
