# Supplementary material for: Understanding epigenetic regulation in nonmodel plants: transcriptomic responses to seed demethylation in leaves and roots of the annual herb Erodium cicutarium (Geraniaceae)
Source: G3 (Bethesda). 2026 May 15;16(8):jkag129. doi: 10.1093/g3journal/jkag129 (PMC13439943; doi:10.1093/g3journal/jkag129)
Supplement: Supplementary file 1 [file jkag129_supplementary_data.zip › Supplementary_Figure_S4_G3-2026-406718.pdf]

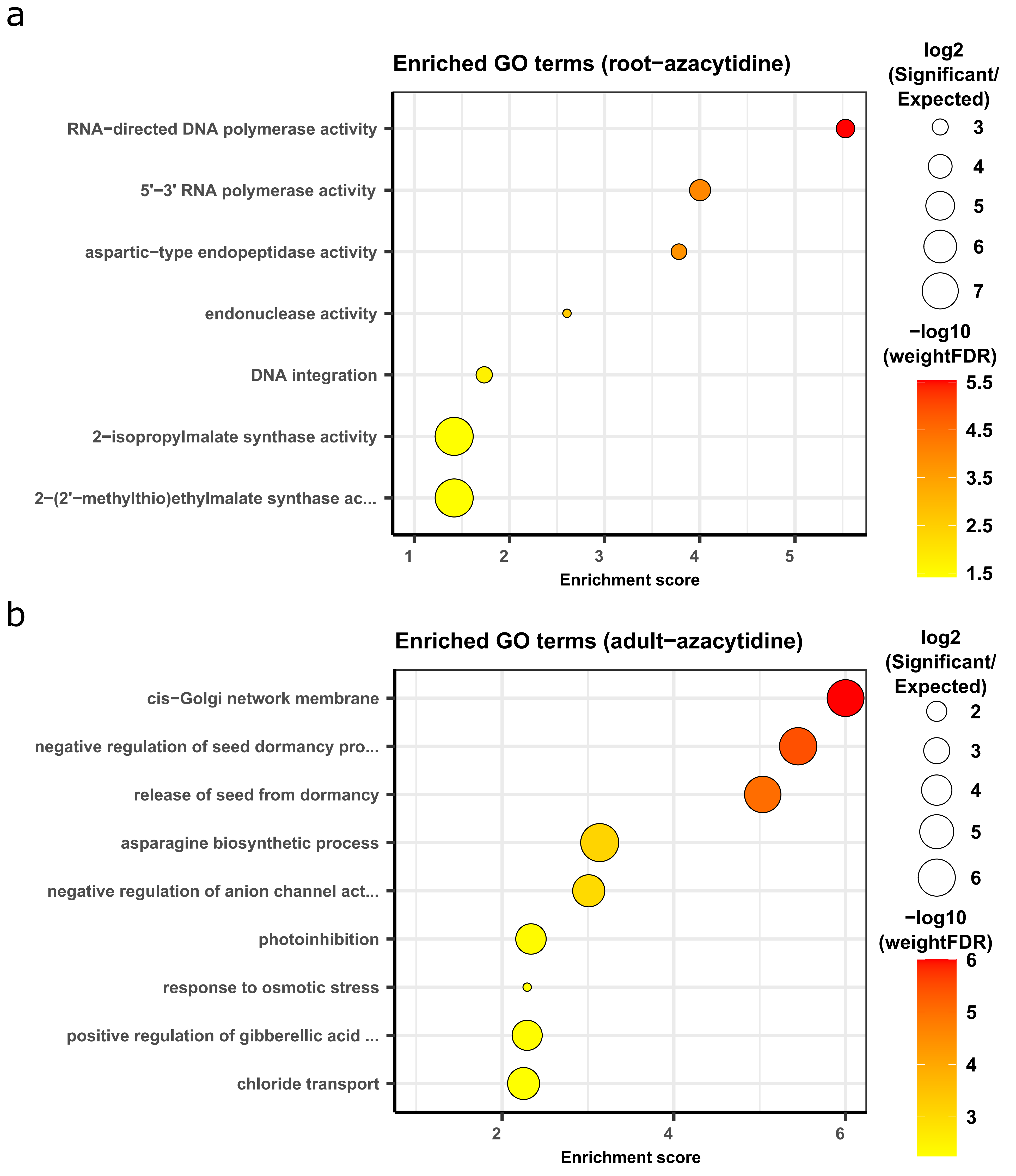
a
Enriched GO terms (root−azacytidine)
RNA−directed DNA polymerase activity
5'−3' RNA polymerase activity
aspartic−type endopeptidase activity
endonuclease activity
DNA integration
2−isopropylmalate synthase activity
2−(2'−methylthio)ethylmalate synthase ac...
1
2
3
4
5
Enrichment score
log2 (Significant/ Expected)
3
4
5
6
7
−log10 (weightFDR)
5.5
4.5
3.5
2.5
1.5
b
Enriched GO terms (adult−azacytidine)
cis−Golgi network membrane
negative regulation of seed dormancy pro...
release of seed from dormancy
asparagine biosynthetic process
negative regulation of anion channel act...
photoinhibition
response to osmotic stress
positive regulation of gibberellic acid ...
chloride transport
2
4
6
Enrichment score
log2 (Significant/ Expected)
2
3
4
5
6
−log10 (weightFDR)
6
5
4
3
