# Supplementary material for: Understanding epigenetic regulation in nonmodel plants: transcriptomic responses to seed demethylation in leaves and roots of the annual herb Erodium cicutarium (Geraniaceae)
Source: G3 (Bethesda). 2026 May 15;16(8):jkag129. doi: 10.1093/g3journal/jkag129 (PMC13439943; doi:10.1093/g3journal/jkag129)
Supplement: Supplementary file 1 [file jkag129_supplementary_data.zip › Supplementary_Figure_S5_G3-2026-406718.pdf]

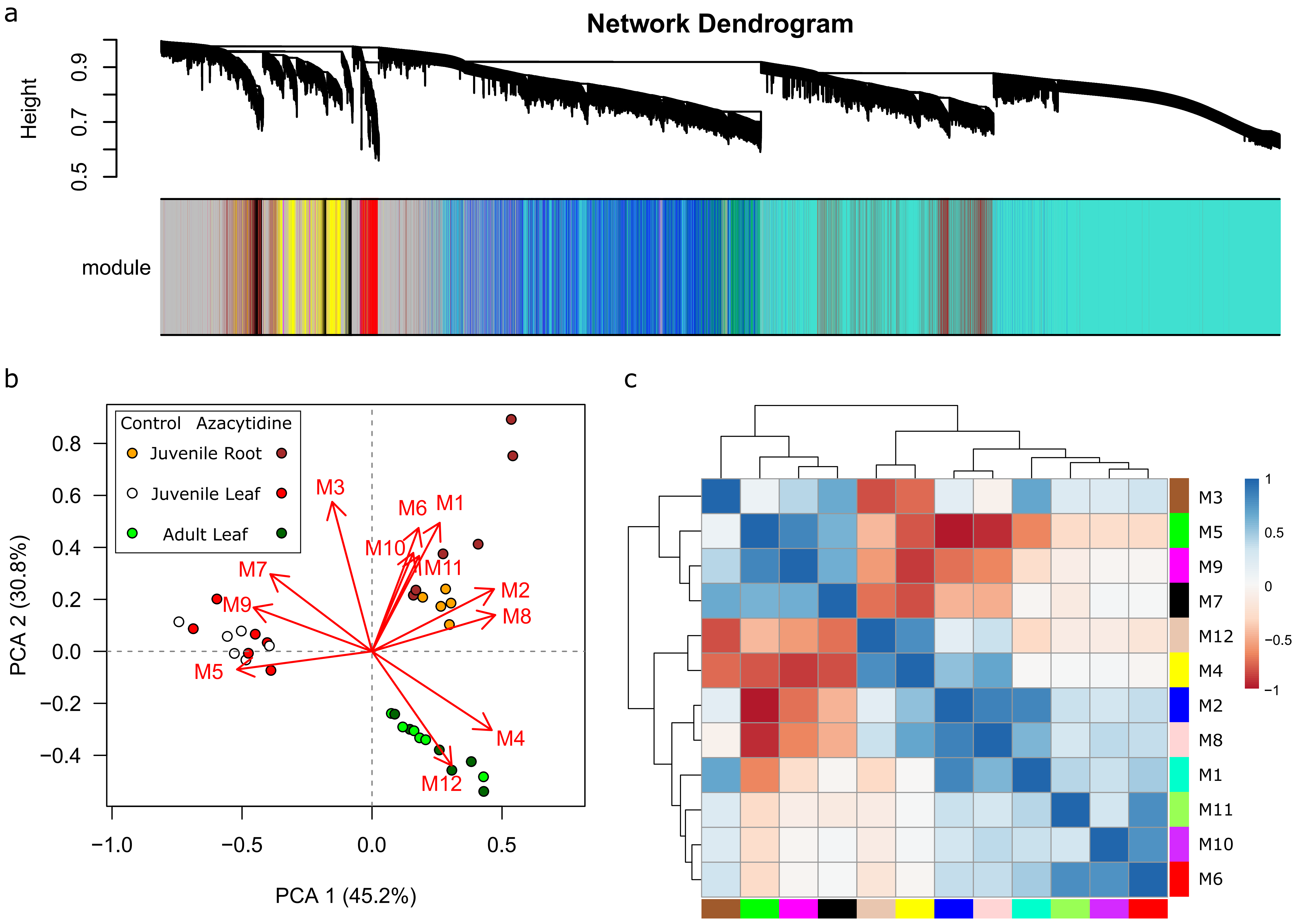
a
Network Dendrogram
Height
0.5
0.7
0.9
module
b
Control
Azacytidine
Juvenile Root
Juvenile Leaf
Adult Leaf
M1
M2
M3
M4
M5
M6
M7
M8
M9
M10
M11
M12
PCA 2 (30.8%)
PCA 1 (45.2%)
0.8
0.6
0.4
0.2
0.0
−0.2
−0.4
−1.0
−0.5
0.0
0.5
c
M3
M5
M9
M7
M12
M4
M2
M8
M1
M11
M10
M6
1
0.5
0
−0.5
−1
