# Supplementary material for: Understanding epigenetic regulation in nonmodel plants: transcriptomic responses to seed demethylation in leaves and roots of the annual herb Erodium cicutarium (Geraniaceae)
Source: G3 (Bethesda). 2026 May 15;16(8):jkag129. doi: 10.1093/g3journal/jkag129 (PMC13439943; doi:10.1093/g3journal/jkag129)
Supplement: Supplementary file 1 [file jkag129_supplementary_data.zip › Supplementary_Figure_S6_G3-2026-406718.pdf]

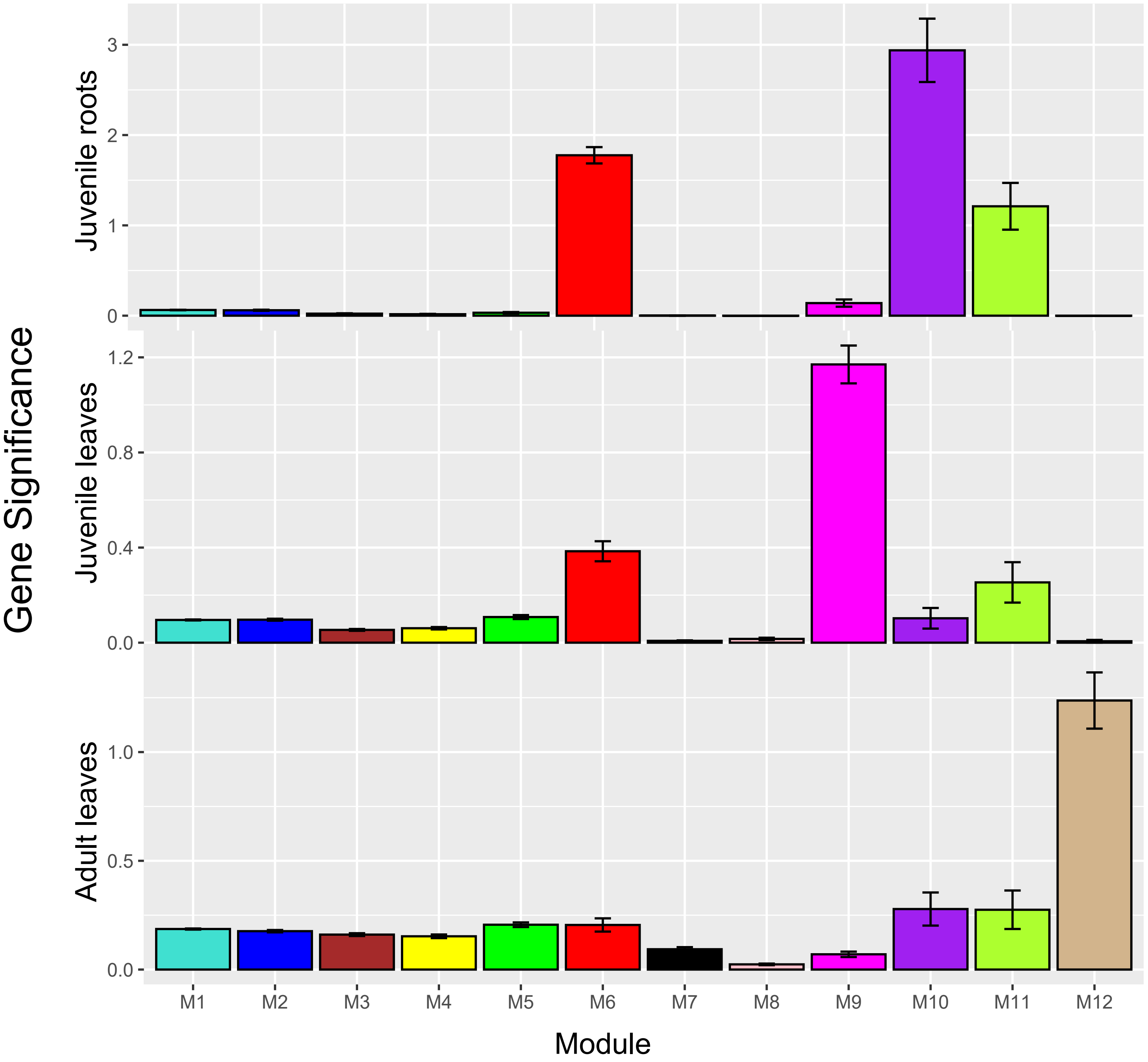
Gene Significance
Juvenile roots
Juvenile leaves
Adult leaves
0
1
2
3
0.0
0.4
0.8
1.2
0.0
0.5
1.0
M1
M2
M3
M4
M5
M6
M7
M8
M9
M10
M11
M12
Module
