# Supplementary material for: Understanding epigenetic regulation in nonmodel plants: transcriptomic responses to seed demethylation in leaves and roots of the annual herb Erodium cicutarium (Geraniaceae)
Source: G3 (Bethesda). 2026 May 15;16(8):jkag129. doi: 10.1093/g3journal/jkag129 (PMC13439943; doi:10.1093/g3journal/jkag129)
Supplement: Supplementary file 1 [file jkag129_supplementary_data.zip › Supplementary_Figure_S7_G3-2026-406718.pdf]

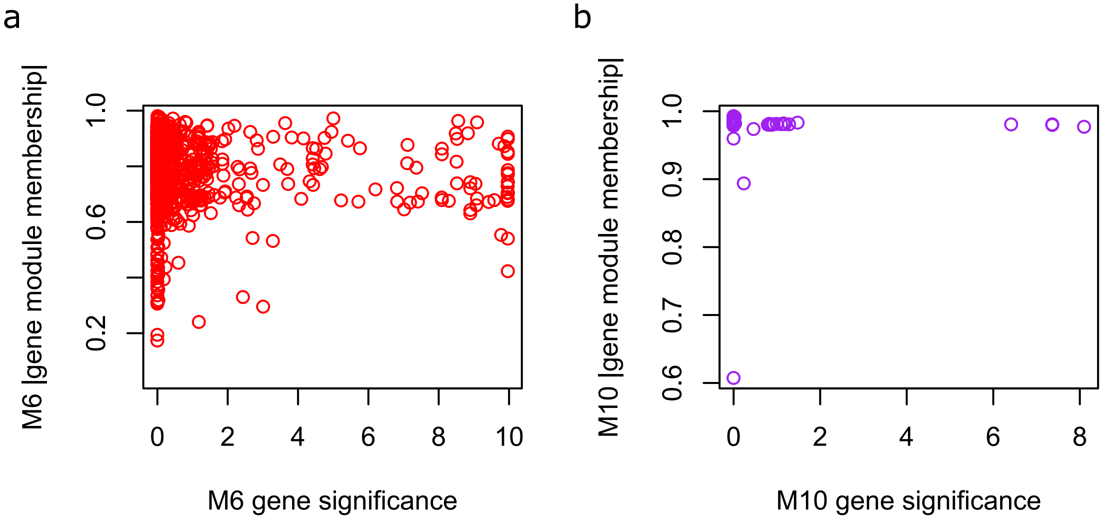
a
M6 |gene module membership|
1.0
0.6
0.2
0
2
4
6
8
10
M6 gene significance
b
M10 |gene module membership|
1.0
0.9
0.8
0.7
0.6
0
2
4
6
8
M10 gene significance
